# Modification of Deoxyribonucleic Acid with Indole-Linked Nucleotides Induces BZ- and Z‑Conformation and Alters Its Sensitivity to Enzymatic Cleavage

**DOI:** 10.1021/acsomega.5c03997

**Published:** 2025-09-23

**Authors:** Suresh Lingala, Anastasiia Fisiuk, Michelle Stephen, Raja Mohanrao, Judah Klingsberg, Simon Vecchioni, Ealonah S Volvovitz, Sergei Rozhkov, Prabodhika Mallikaratchy

**Affiliations:** 1 Department of Molecular, Cellular and Biomedical Sciences, 465154 City University of New York School of Medicine, 160 Convent Avenue, New York, New York 10031, United States; 2 PhD Programs in Chemistry and Biochemistry, Graduate Center, City University of New York, 111 Fifth Avenue, New York, New York 10065, United States; 3 PhD Program in Biology, Graduate Center, City University of New York, 365 Fifth Avenue, New York, New York 10065, United States; 4 Department of Biology, City College of New York, 160 Convent Avenue, New York, New York 10031, United States; 5 Department of Chemistry, 5894 New York University, New York, New York 10003, United States

## Abstract

We report the synthesis of C-5 indole-tagged pyrimidine and C-8 indole-tagged purine nucleoside phosphoramidites and their incorporation into a 15-base antiparallel DNA duplex. The resulting modified duplexes adopt noncanonical conformations, including modified B-DNA conformations, BZ junctions, and left-handed Z-DNA, under physiological conditions, bypassing the specific sequence requirements and high salt concentrations typically required for BZ or Z-DNA formation. Using a panel of twenty-three duplexes containing one to five indole-modified bases linked via either propyl or propargyl linkers, we demonstrate that overall duplex conformation is strongly influenced by propyl-linked indole modifications at dA/dU positions. Among the two linker types tested, the flexible propyl linker promoted conformational plasticity, enabling transitions to BZ or Z-like structures under physiological conditions. In contrast, duplexes containing the more rigid propargyl linkers retained canonical B-form conformations. Modifications placed within or near restriction enzyme recognition sites highlighted the importance of linker flexibility in modulating enzymatic recognition and cleavage. Duplexes with a high density of modifications, particularly those modified on both strands with propyl-linked indole, exhibited marked resistance to digestion by DNase I, *Eco*RI, *Sma*I, and *Xma*I. Termed “Z-inducing chimeras” (ZImeras), these duplexes represent a versatile platform for investigating the biological roles of noncanonical DNA structures, expanding the toolkit for exploring and controlling non-B-DNA conformations in both basic research and therapeutic applications.

## Graphical abstract

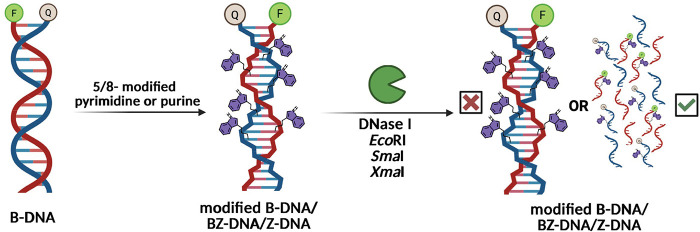

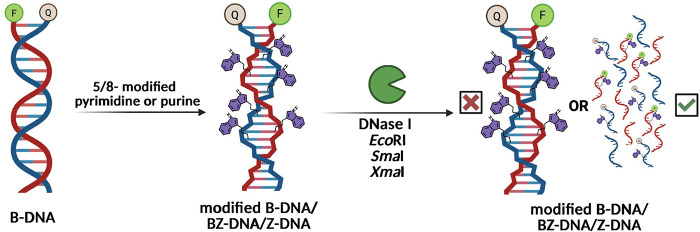

## Introduction

Growing evidence suggests that the noncanonical conformation (NCC) of nucleic acids plays a major role in regulating cellular activities.^1,2^ The important, yet not fully understood, role of NCC is further validated by data obtained from sequencing the human genome, which shows that more than 50% of it is composed of repeat DNA, out of which 13% of total DNA is composed of simple sequence repeats that can form NCC.^3^ Owing to the poor understanding of NCC in regulating cellular function, the occurrence of NCC was initially considered to have no biological significance. However, recent discoveries related to NCC suggest otherwise. Repetitive sequences with a high propensity to form NCC have been identified in promoter regions and replication origins, suggesting their pivotal role in biological processes.^1^ While the occurrence of NCC is transient, driven by unfavorable folding, these conformations still seem to play a decisive role in the regulation of DNA replication and gene expression, altering DNA–protein interactions, transcription and translation, and processing of pre-mRNA.^4–9^ The most common NCC include G-quadruplex formation, i-motifs, R-loops, triplex and cruciform structures, supercoiling, bubbles, H-DNA, A-DNA, Z-DNA, and BZ junction structures.^3,10–12^

The left-handed form of DNA, termed Z-DNA, was initially discovered by Rich and co-workers.^13^ It is found in viruses, bacteria, yeast, flies, and mammals, including humans.^14–17^ Z-DNA formation is predominantly driven by alternating *syn*- and *anti*-base conformation of GC dinucleotide repeat sequences.^13^ The existence of Z-DNA conformation in the promoter sites is implicated in cancer, autoimmune diseases, and neurological diseases, such as Alzheimer’s disease.^8,18–20^ Z-DNA binding proteins, the class of proteins that recognize the left-handed Z-form of DNA and RNA, include important enzymes. For example, adenosine deaminase acting on RNA 1 (ADAR1) is involved in RNA editing by converting adenosine to inosine, and it plays a key role in cancer and autoimmune disorders.^21–24^ Intriguingly, ADAR has a characteristic affinity toward Z-DNA.^25^ Despite repeated observations of the presence of Z-DNA structures and their implications for altering cellular processes, the investigation of Z-DNA in both cellular and animal models has been challenging. This can be attributed to the instability of Z-DNA structures and the transient appearance of Z-DNA in cellular events, thus limiting the fundamental understanding of the Z-DNA structure and function.

To generate Z-DNA conformers, Gannett and others incorporated C-8 aryl guanine in short oligos and successfully demonstrated the stable formation of sequence-dependent Z-DNA at physiological conditions.^26^ Another way of inducing stability of Z-conformation is by introducing high concentrations of cations or spermine, spermidine, hexamine cobalt, and ruthenium complexes to neutralize the negative charge of the zigzag phosphate backbone.^27,28^ Sugiyama and co-workers demonstrated that synthetic sequence-specific methylated guanosine adducts still require high salt concentrations to maintain a stable sequence-dependent Z-DNA conformer *in vitro*.^29^ Recently, Gonzalez and his colleagues showed that C2′-fluorinated nucleic acids could adapt Z-conformations of nucleobases in a sequence-specific manner at physiological conditions.^30^

Herein, we aimed to investigate whether the formation of Z-DNA or BZ-DNA could be induced independently of the sequence context by introducing complementary strands modified with C-5-propyl/propargyl indole-substituted pyrimidines (dU, dC) and C-8-propyl/propargyl indole-substituted purines (dA, dG) into a 15-nucleotide-long DNA duplex. Inspiration to use indole was drawn from the privileged molecular interactions mediated by the indole moiety in amino acids and a large number of currently available pharmaceuticals.^31^ Indole’s unique chemical characteristics arise from its ability to form amphipathic and dipole interactions via its imino hydrogen and hydrophobic interactions via the aryl group, owing to its bicyclic nature. Indole derivatives, such as tetracyclic indoloquinolines, have been shown to bind to double-stranded, triple-stranded, and quadruplex DNA structures, establishing indole-based compounds as promising DNA intercalating agents.^32^ Additionally, indole has been readily used in understanding NCC, such as triplex structures that arise by the ability of indole to act as both hydrogen bond donors and acceptors with nitro- or formyl indole-modified nucleosides.^33,34^ To investigate how indole modifications affect the biochemical properties of DNA duplexes, we designed the sequences for use with restriction enzymes, namely, *Eco*RI, *Xma*l, and *Sma*l sites. Then, we strategically incorporated modified nucleotides directly on the *Eco*RI restriction site, which is near the *Xma*I/*Sma*I site, to learn how conformational adaptation of a duplex would, as a result of these modifications, lead to NCC and, moreover, how the resulting NCC would impact the promiscuity of the duplex toward restriction enzymes. Structural characterization of the duplexes using CD spectroscopy revealed that two out of twenty-three tested duplexes adopted a Z-DNA conformation in physiological conditions with no specific sequence requirement, while several others exhibited features consistent with BZ junctions and modified B to canonical B conformations. Following melting point analysis, we examined the DNase I susceptibility of the duplexes. We observed that the modified duplexes showed variable sensitivities to DNase I. Restriction digestion experiments revealed that the addition of indole-modified bases within the *Eco*RI restriction site significantly influenced the restriction of *Eco*RI’s activity. On the other hand, the recognition and activity of *Xma*I and *Sma*I varied based on the position of the modification.

## Materials & Methods

All chemicals and solvents were purchased from Sigma-Aldrich, Fisher Scientific, Oakwood Chemicals, and Ambeed Chemicals and used without further purification. Reactions were monitored by thin-layer chromatography (TLC) using Merck precoated silica plates (Silica Gel 60 F254, 0.25 mm). TLC plates were visualized under ultraviolet light at 254 nm and by charring using ceric ammonium molybdate and p-anisaldehyde solutions. Chromatography was performed on a Teledyne ISCO CombiFlash Rf 200i instrument using disposable silica cartridges. ^1^H, ^13^C and ^31^P NMR spectra were acquired on an OXFORD NMR AS500 (^1^H at 500.0 MHz, ^13^C at 126.0 MHz, and ^31^P at 202.0 MHz). Residual solvent peaks were used as internal references and expressed in parts per million (ppm). Spin multiplicities were represented as s (singlet), d (doublet), t (triplet), q (quartet), dd (doublet of doublet), ddd (doublet of doublet of doublet), dt (doublet of triplet), m (multiplet), and brs (broad singlet), while coupling constants (*J*) are given in Hertz. Mass spectra were recorded by either the Proteomics Facility at the Advanced Science Research Centre, CUNY, or Novatia, LLC.

### Synthesis of Indole Coupled Base Modified Nucleoside Phosphoramidites

All phosphoramidites were synthesized using established synthetic methods; please see the Supporting Information for detailed synthetic methodologies and characterization of all the intermediates and final phosphoramidites.

### Solid-Phase Synthesis of DNA Sequences and Purification

All DNA reagents required for DNA synthesis were purchased from Glen Research. Wild-type (WT) and complementary wild-type (cWT) DNA sequences were purchased from Integrated DNA Technologies. All modified DNA sequences were synthesized by following standard solid-phase DNA synthesis protocols on an ABI394 DNA synthesizer (Biolytics) on a 0.2 μmol scale. Synthesis was performed in standard mode using 2-cyanoethyl-*N*,*N*-diisopropylphosphoramidites. A 0.1 M solution of each phosphoramidite and 0.25 M 5-ethylthio-1-*H*-tetrazole, as an activator, was used for DNA synthesis. Iodine solution (0.02 M Iodine in THF/Py/Water) was used for oxidizing the phosphoramidites. The reaction volume and duration for coupling natural phosphoramidites were 220 μL and 90 s, respectively, while for modified phosphoramidites, the reaction volume was kept at 220 μL, and the coupling time was extended to 600 s. The DNA deprotection was carried out by incubating DNA on CPG with 30% aq. NH_4_OH (37 °C, 24 h). DNA sequences were purified using a high-performance liquid chromatograph (Agilent) equipped with a C-18 reverse-phase column (Phenomenex). We used 0.1 M triethylammonium acetate (TEAA) and acetonitrile as mobile phase, and a linear gradient from 5% to 100% acetonitrile over 65 min was used to elute oligonucleotides. A UV–vis spectrophotometer (Agilent) was employed to quantify the purified DNA to obtain the stock concentration.

#### CD Spectral Analysis

Circular dichroism (CD) measurements were conducted by using a Jasco-1500 circular dichroism spectrometer. The data were collected using a quartz cuvette with a 10 mm path length, in the wavelength range of 220–330 nm, with a data pitch of 2 nm, an integration time of 8 s, a spectral bandwidth of 1 nm, and a scanning speed of 20 nm/min. Three accumulations were averaged for each spectrum. A blank measurement was first taken with 400 μL of PBS buffer, and 200 pmol of the DNA samples in 400 μL of PBS buffer were analyzed. Blank spectra were subtracted from the sample spectra and were subsequently smoothed and zeroed at 320 nm.

#### Melting Point Measurements

To analyze the thermal stability of the duplexes, 50 nM of each duplex was prepared by incubating 10 μmol of fluorescently labeled template oligonucleotide sequence with 25 μmol of dabcyl labeled complementary oligonucleotide sequence in 200 μL of PBS (Gibco 1× PBS, pH = 7.4). Each duplex sample was placed into a reduced-volume quartz cuvette (Horiba, path length = 3 mm), and fluorescence intensity was measured using the FluoroMax Plus spectrofluorometer (Horiba) with a TC1 temperature controller (Quantum Northwest) and an EXT-440 liquid cooling system (Koolance). The emission wavelength utilized in the thermal stability assays correlated with fluoresceine (λ_em_ = 514 nm), with the excitation wavelength at λ_ex_ = 488 nm, and slit width of 2.5 nm. Measurements were taken at 1 °C intervals (tolerance = ± 0.5 °C) across a temperature range of 5 to 70 °C.

#### Enzymatic Experiments

We investigated the impact of indole-modified bases on oligonucleotide sequences and their susceptibility to digestion by endonucleases on the duplexes. For each assay, the 10 μmol duplex samples were prepared by incubating 10 μmol of fluorophore-labeled oligonucleotide sequence with 25 μmol of quencher-labeled complementary oligonucleotide sequence (sample volume: 10 μL). After the addition of the respective endonuclease, the reaction samples were incubated for the specified amount of time in a TC 9639 thermal cycler (Benchmark). For DNase I digestion assays, the duplex samples were treated with 1.4 units of DNase I (Thermo Scientific, REF EN0521, 0.56 u/μL). The reactions were incubated for 15 min at 37 °C. For *Eco*RI digestion assays, the duplex samples were treated with 70 units of *Eco*RI-HF (NEB, R3101M, 28 u/μL). The reactions were incubated overnight in a thermocycler at 37 °C. For *Xma*I digestion assays, the duplex samples were treated with 7 units of *Xma*I (NEB, R0180S, 2.8 u/μL). The reactions were incubated overnight at 37 °C. For *Sma*I digestion assays, the duplex samples were treated with 7 units of *Sma*I (Thermo Scientific, REF# ER0665, 2.8 u/μL) at 25 °C. To assess enzymatic digestions in all endonuclease digestion assays, the treated duplex samples were diluted with 185 μL of 1X PBS (Duplex Cf = 50 nM) postincubation, and fluorescence measurement was performed at 20 °C. The emission wavelength range utilized for measuring treated duplex samples was λ_em_ = 500 to 600 nm, and the excitation wavelength was λ_ex_ = 488 nm, with a 2.5 nm slit width. An unmodified, wild-type duplex was used as the control and was measured alongside each modified duplex to ensure consistency in conditions between each experiment.

#### Data Analysis for Bar Diagrams

FRET data analysis for endonuclease assays included baseline correction and normalization. During the baseline correction step, the background fluorescence intensity at 514 nm from the untreated sample of each duplex was subtracted from that of the corresponding enzyme-treated sample. The corrected fluorescence values were then normalized to the wild-type (WT) duplex using the following equation:

$$\mathrm{Normalized}\;\mathrm{Fluorescence}\;\mathrm{Intensity}=\frac{\left(\mathrm{Fluorescence}\;\mathrm{Intensity}\;\mathrm{of}\;\mathrm{modified}\;\mathrm{duplex}\;\mathrm{with}\;\mathrm{enzyme})-(\mathrm{Fluorescence}\;\mathrm{Intensity}\;\mathrm{of}\;\mathrm{mod}\mathrm{ified}\;\mathrm{duplex}\;\mathrm{without}\;\mathrm{enzyme}\right)}{\left(\mathrm{Fluorescence}\;\mathrm{Intensity}\;\mathrm{of}\;\mathrm{wild}\;\mathrm{type}\;\mathrm{duplex}\;\mathrm{without}\;\mathrm{enzyme})-(\mathrm{Fluorescence}\;\mathrm{Intensity}\;\mathrm{of}\;\mathrm{wild}\;\mathrm{type}\;\mathrm{duplex}\;\mathrm{with}\;\mathrm{enzyme}\right)}\times 100$$

λ_max_ = 514 nm and measured temperature 20 °C.

The average of the normalized values from three replicates was calculated for each duplex and used to generate bar diagrams, providing a visual representation of each duplex that showed relative susceptibility to endonuclease activity compared to that of WT.

## Results

### Synthesis of ZImera Duplexes

We synthesized the corresponding nucleoside phosphoramidites (**1a**–**4a**) *via* classical Sonogashira cross-coupling with 3-prop-2-ynyl-indole, followed by hydrogenation, protection of the hydroxyl and amine groups, and subsequent conversion to phosphoramidites. Compounds **1b**–**4b** were synthesized using the same approach without hydrogenation (Scheme 1).

**Scheme 1:**
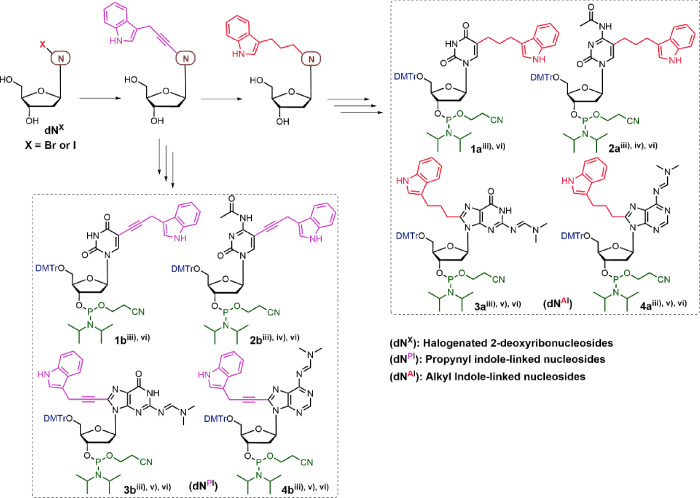
Design and Synthesis of Propyl and Propargyl-Linked Indole-Modified Nucleoside Phosphoramidites^a^

a Reagents and conditions: (i) 3-Prop-2-ynyl-indole, Pd­(PPh_3_)_4_, CuI, Et_3_N, DMF, 55–60 °C, 2–3 h; (ii) H_2_, 10% Pd/C, MeOH, 50 °C, 12 h; (iii) DMTrCl, DMAP, pyridine, rt, 12 h; (iv) Ac_2_O, DMF, 0 °C to rt, 8–10 h; (v) DMF-DMA, DMF, rt, 1 h; (vi) 2-Cyanoethyl-*N*,*N*-diisopropylchlorophosphoramidite, DIPEA, DCM, 0 °C to rt, 1.0–1.5 h. Detailed synthetic procedures and corresponding spectroscopic characterizations of intermediates and final phosphoramidites are provided in the Supporting Information.

We used standard solid-state chemistry on an ABI394 DNA synthesizer to synthesize the short oligos listed in Table 1, using coupling times extended to 600 s to couple **1a/b**, **2a/b**, **3a/b**, and **4a/b**. We did not observe any reduction in coupling efficiency for propyl-linked indole-modified nucleoside phosphoramidites **1a**, **2a**, **3a**, and **4a**. Based on DMT detection, the overall yield of Z1-Z6 and cZ1-cZ5 oligos was approximately 80%–85%, which was comparable to the yield of wild-type (WT) sequences, suggesting that modification at C-5 and C-8 using flexible propyl (Pr) linkage does not impact coupling efficiency. However, we observed a steep decline in the coupling efficiency of propargyl-linked indole-modified nucleoside phosphoramidites (**1b**, **2b**, **3b**, and **4b**), reducing the overall yield to 15%–20% for Z11-Z55 oligonucleotides, respectively. Hocek and others observed similar coupling efficiencies for nucleoside phosphoramidite derivatives in the synthesis of hypermodified DNA oligos containing 5-phenylethynyluracil, 5-(pentyn-1-yl)­cytosine, 7-(indol-3-yl)­ethynyl-7-deazaadenine, and 7-isopropylethynyl-7-deazaguanine, suggesting that rigidity of the linker might play a role in orienting the activated 3′ amidite moiety during DNA synthesis.^35^

**Table 1:**
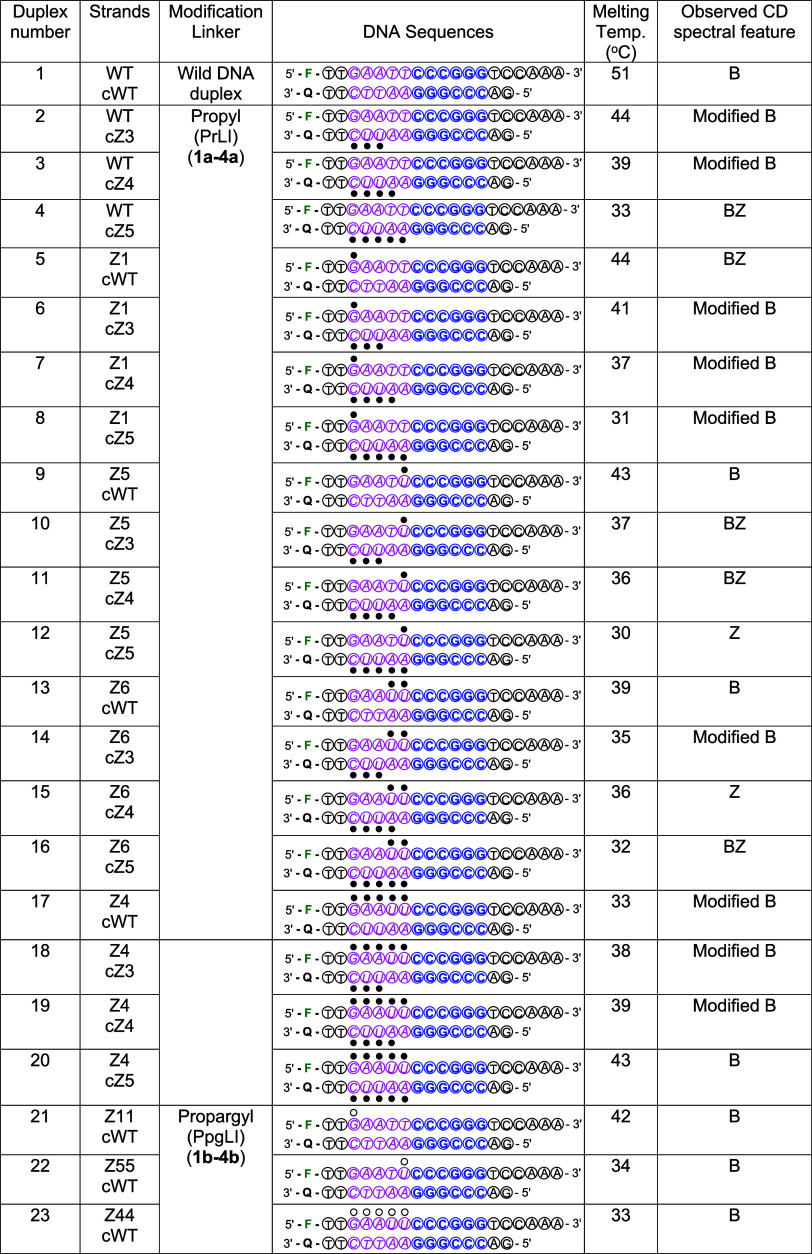
List of Oligonucleotide Duplexes^a^

a Solid circles: 3-propyl-indole-linked nucleotide; open circles: 3-(prop-2-yn-1-yl)-indole-linked nucleotide; F: 5′-(6-FAM)-labeled; Q: 3′-(Dabcyl)-labeled. Italics pink *Eco*RI restriction site; bold: blue *Xma*l, *Sma*l restriction site.

### Thermal Stability and CD Spectral Features of ZImera

Since modifications at the C-5 and C-8 positions of nucleobases are known to destabilize duplexes, we first compared the annealing behavior and the corresponding melting temperatures of duplexes 1 to 23 to those of the wild-type duplex (Table 1, Figures S1 and S2). The wild-type duplex exhibited a melting temperature of 51 °C, while all duplexes with one to three deoxy cytidine and/or deoxy uridine modifications with propyl-linked indole (PrLI) show a drop in melting by 6 °C. For instance, duplex 2 demonstrated a decrease in melting temperature by 7 °C compared to that of the wild-type duplex. The melting temperature decreased by approximately 5 °C with each additional PrLI-modified dA in duplexes 3 and 4. Confirmation of the DNA structure by CD spectroscopy is well established. For example, left-handed Z-DNA, an unusual form of DNA structure, was first identified using CD spectroscopy, demonstrating the accuracy of this technique in clearly depicting structural deviations in DNA.^17,36,37^ Therefore, we used CD spectroscopy to assess the structures of duplexes 1–23. Notably, modified B-DNA was observed following modification with r-7,t-8-dihydroxy-t-9,10-oxy-7,8,9,10-tetrahydrobenzo­[a]­pyrene (BPDE). Increasing levels of BPDE modification result in a blue shift and increased molar ellipticity, consistent with structural perturbations caused by the covalent attachment of BPDE, suggestive of modified B-DNA.^38^ The introduction of bulky substituents, such as cholesterol, has also been shown to distort DNA duplexes comprising ten base pairs.^39^ Furthermore, Vorlíčková et al. reported a conformational shift in poly­(dA-dT) sequences at high CsF concentrations, suggesting that repeated poly­(dA-dT) duplexes can undergo gradual transitions from canonical B-form to unusual B-form, BZ-form, and Z-form conformations as a function of increasing ionic strength, similar to that observed in alternating GC repeats.^36,37^ The transitions observed for poly­(dA-dT)•poly­(dA-dT) in the presence of CsF produced a reduction in the positive CD band at 280 nm, indicative of a noncooperative winding transition within a B-form DNA duplex.^37^ In our study, we observed similar unusual spectral transitions across all duplexes with increasing levels of PrLI modification, characterized by varying degrees of decreased or increased molar ellipticity near 280/245 nm, which suggests the formation of unusual B-DNA conformations potentially comprising modified B-DNA forms.^40,41^ For example, as the number of PrLI modifications increased, the conformation of duplexes 2–4 progressively shifted from the B-form to a BZ-like conformation (Table 1, Figure 1B–D). This trend continued in duplexes 5–8, where we observed that duplex 5 adopted a BZ conformation, while duplexes 6–8 adopted modified B-form and progressively lower melting temperatures, correlating with the number of PrLI modifications on the complementary strand (Table 1, Figure 1F–H). Next, duplexes 9–12 showed a structural transition from B-form in the least PrLI-modified duplex 9 to BZ in duplexes 10–11 and ultimately to Z-form in duplex 12, indicating that the introduced uridine and adenine PrLI modifications on the complementary strand had influenced both duplex stability and conformation (Table 1, Figure 1I–L). Duplexes 13–16 followed a similar consistent trend, wherein the minimally modified duplex adopted a canonical B-form and exhibited the highest thermal stability (Table 1, Figure 1M–P). However, progressive incorporation of PrLI modifications on the complementary strand induced a conformational shift from modified B-form to BZ and ultimately Z-DNA, correlating with a stepwise reduction in the melting temperature. These findings further validate that an increased PrLI modification density drives conformational transitions and destabilizes duplex thermal stability. Additionally, two PrLI-modified dU that are adjacent in duplexes 13–16, and those duplexes both exhibit the lowest melting temperatures and adopt more distorted conformations. Given that the duplexes contain a palindromic d­(AAUU) sequence in the *Eco*RI site, the PrLI modifications introduced at the dU and dA positions may have derived conformational inversion even in the absence of high salt, likely owing to the steric effects of the bulky indole moiety forcing this conformational adaptation. Duplexes 17–20, which contained the highest density of PrLI-modified bases on the template strand, exhibited an inverse trend whereby the least-modified duplex (17) showed the lowest melting temperature, whereas the most extensively PrLI-modified duplex (20) displayed the least destabilizing effect. With the increasing number of PrLI modifications introduced on the complementary strand, duplex conformation progressively shifted from a modified B-form to a canonical B-form, indicating increased structural stabilization with greater modification density on both strands (Table 1 and Figure 1Q–T). The stacking effect through π–π interactions involving the indole moiety within the adeninium ring has been previously reported.^42^ This suggests that the successive incorporation of PrLI-modified dAs may have contributed to the enhanced duplex stability.^42^ Additional PrLI dG modifications did not lead to substantial reductions in the melting temperature. These findings may indicate that PrLI dU and PrLI dA have a greater impact on conformational transitions. Overall, the melting temperature is most strongly impacted when the 3′-d­(CUUAA)-5′ sequence is modified with PrLI (Table 1, Figures S1and S2). Duplexes 21–23, containing propargyl-linked indole (PpgLI) modifications, adopted a canonical B-form (Table 1 and Figure 1U–W). Consistent with previous observations, an increasing number of PpgLI modifications on the template strand resulted in a progressive decrease in melting temperature (Table 1 and Figure 1U–W).

**Figure 1:**
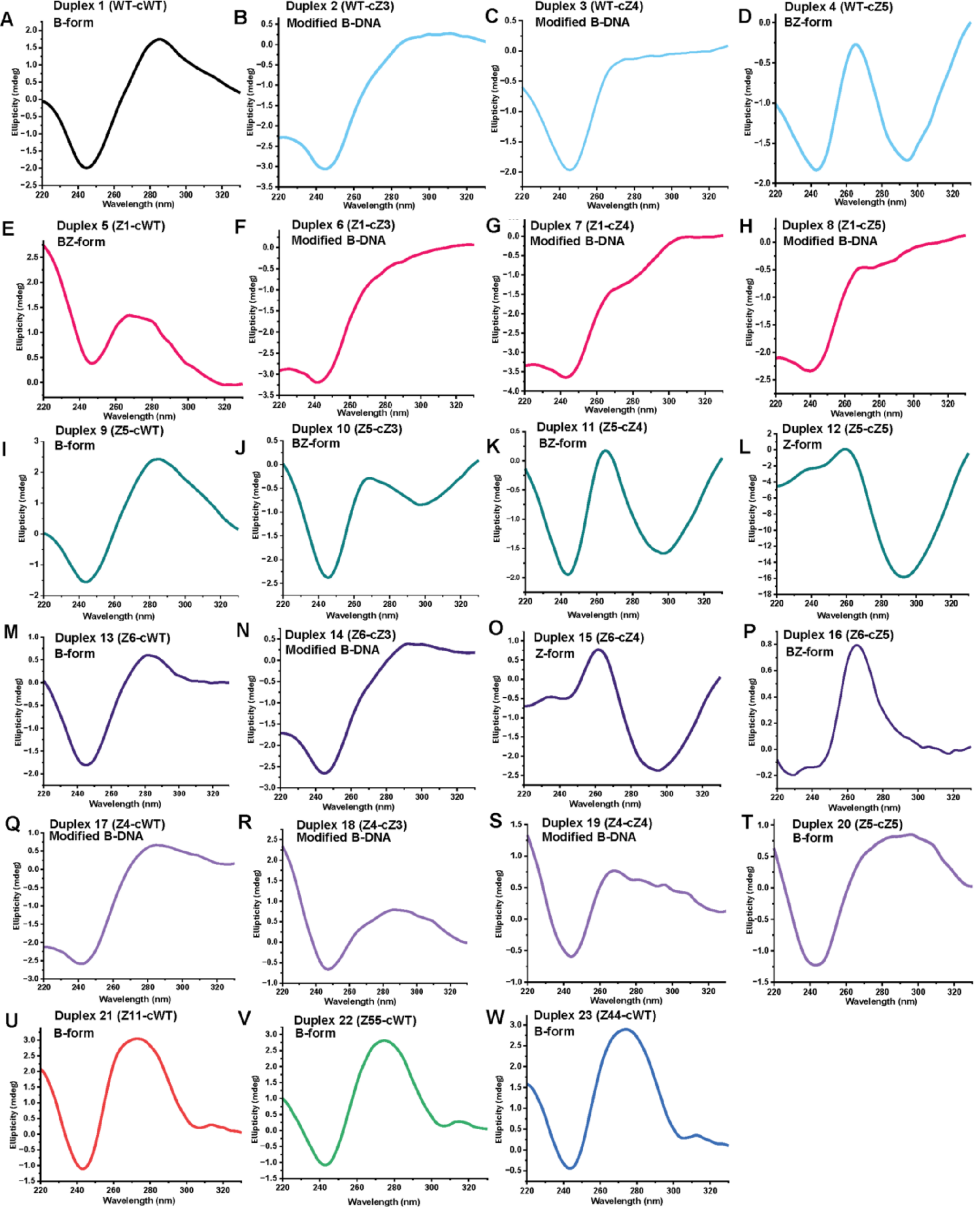
CD analysis of ZImera. CD spectra of ZImera were recorded at 25 °C using a 10 mm path length quartz cuvette in the range of 220–330 nm with a scanning speed of 20 nm/min, 1 nm bandwidth, 8 s response time, and three accumulations. Modified B-DNA: B-DNA structure exhibits deviations in molar ellipticity at 280/245 nm, as detected by CD spectroscopy, suggesting alterations to the canonical B-form while retaining overall B-DNA characteristics.

### DNase I and Restriction Enzyme Digestion of ZImera

Since most NCC of nucleic acids and modified nucleic acids are resistant to DNase I, we next assessed the sensitivity of the duplexes toward DNase I cleavage (Figure 2A,B, Figure S3). Despite the known high promiscuity of DNA duplexes toward nuclease activity mediated by DNase I, we observed that the sensitivity of the modified duplexes toward DNase I varied based on the position of the PrLI modification. For example, the number of PrLI modifications on the complementary strand of duplexes 1–4 was strategically increased one base at a time. Results showed a progressive reduction in DNase I sensitivity with an increasing PrLI modification density. Among all duplexes, except duplex 17, the least PrLI-modified duplex exhibited the highest susceptibility to DNase I, but enzymatic sensitivity significantly decreased as additional PrLI modifications were introduced, on either the template strand or the complementary strand. DNase I is an endonuclease that nonspecifically, but preferentially, cleaves double-stranded B-DNA at 3′ hydroxyl and 5′ phosphoryl nucleotides using a single-strand nicking mechanism.^43^ Significant nuclease resistance was observed in duplexes 4, 8, 12, 16, and 17–20 (Figure 2A). Among duplexes adopting modified B-form conformations, DNase I sensitivity varied depending on the position and number of PrLI modifications. For instance, duplexes 6–8, with stepwise introduction of PrLI modifications on the complementary strand, demonstrated progressively increased resistance to DNase I. Notably, when the template strand was modified with PrLI, all corresponding duplexes exhibited substantial resistance to enzymatic cleavage, irrespective of modification pattern. The duplexes containing Z6, except for duplex 13 (Figure 2A), significantly deviated in melting points with greater deviations from the B-DNA structure, and showed a lower promiscuity toward DNase I. While DNase I exhibits peak endonuclease activity at 37 °C, it is also capable of cleaving DNA at a lower rate, maintaining its activity at room temperature, albeit with reduced efficiency.^44^ Since the melting temperatures of duplexes 15 and 12 are below 37 °C, we investigated whether their resistance to DNase I cleavage resulted from partial melting into single-stranded structures or the adoption of Z-conformation. When DNase I activity was assessed at 25 °C, we observed no change in DNase I resistance for duplexes 12 and 15 compared to that of the wild-type duplex under the same conditions (Figure S4). Although Z-DNA structures are known to exhibit resistance to nuclease digestion, it remains unclear whether this resistance, such as shown in duplexes 12 and 15, is primarily driven by the structural conformation itself or by the chemical modifications that induce such conformations.

**Figure 2:**
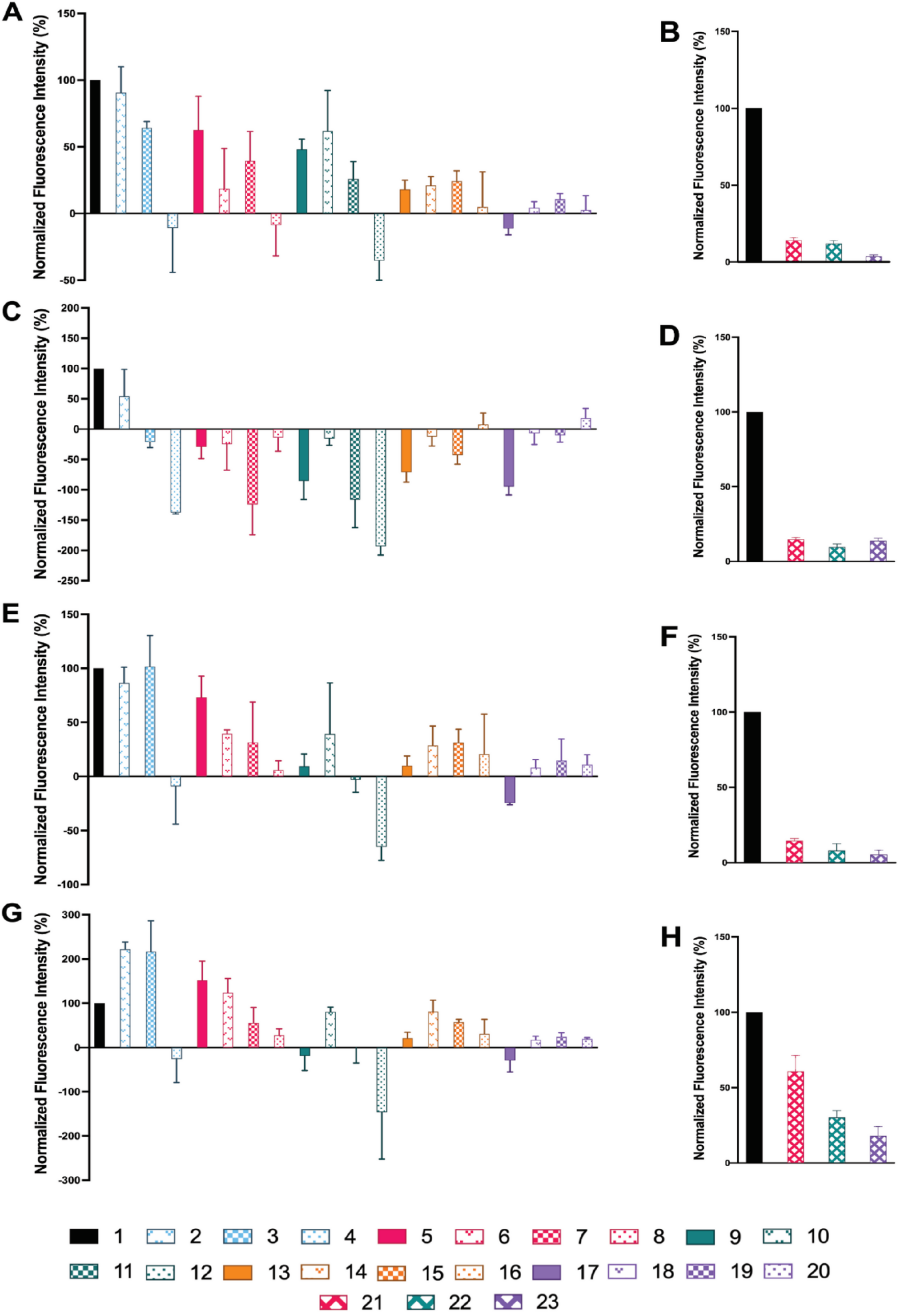
Comparative analysis of the promiscuity of duplexes 1–23 toward DNase I (A, B), *Eco*RI (C, D), *Xma*I (E, F), and *Sma*I (G, H). Enzymatic assays were performed using FRET assays with a fluorophore-labeled template strand and a quencher-labeled complementary strand. Each duplex was incubated at 37 °C with the corresponding enzyme, and enzymatic activity was analyzed using FRET at 20 °C using a fluorescence spectrometer. Fluorescence intensity measured was normalized against that of the control wild-type duplex 1.

We next assessed promiscuity of the duplex toward restriction-modification (R-M) enzymes (Figure 2C−H, Figures S5–S9). R-M enzymes show both nuclease and methylation activities that resemble primitive immune systems that destroy foreign DNA in bacteria.^45^ R-M enzymes are programmed to protect host chromosomes by recognizing and cutting foreign DNA from invading viruses. *Eco*RI is known to first scan DNA duplexes in one dimension to find its cleavage site and then cleave between G and A in a d­(GAATTC) palindrome.^45^ A 2D NMR analysis of dodecamer structure with an *Eco*RI site showed that structural anomalies, such as kinks or increased flexibility of a duplex, facilitate scission.^45^ Modified bases were strategically introduced at the *Eco*RI recognition site, ranging from one to five modifications on both the template and complementary strands. Similar to DNase I, when PrLI modifications were introduced on the complementary strand, at the *Eco*RI recognition site, duplexes 1–4 exhibited a progressive decrease in *Eco*RI sensitivity with increasing modification density (Figure 2C, Figure S5). *Eco*RI did not, however, digest all duplexes containing PrLI-modified restriction site (Figure 2C, Figure S5) except for duplex 2. This resistance is expected as the modifications were introduced directly at the restriction site. Similarly, the resistivity of the duplex was increased when the template strand was modified with **1b**–**4b** (Figure 2D, Figure S5). This line of evidence suggests that the PrLI modification induced steric effects by the indole moiety may abolish the interaction between the duplex and *Eco*RI. To assess whether the reduced melting temperatures contributed to *Eco*RI resistance in duplexes 12 and 15, we evaluated the cleavage efficiency at 25 °C by examining site-specific digestion. No significant differences in cleavage patterns were observed (Figure S6). Similar observations have been reported for propargyl-modified restriction sites wherein the incorporation of bulky aromatic substitutions, such as 7-substituted phenyl or 3-nitrophenyl groups on 7-deazaadenine, was incorporated directly within the restriction enzyme recognition sequence, particularly at critical contact positions, and disruption of base-specific activity was reported.^46^

Finally, we introduced a modification to DNA in close proximity to a specific DNA sequence recognized by a restriction enzyme and evaluated the effect on scission (Figure 2E−H, Figures S7 and S9). The susceptibility of the duplexes to *Sma*I and *Xma*I cleavage also appears to be influenced by the position of PrLI modifications, even when they are not directly located within the recognition or cleavage sites (Figure 2E−G, Figures S7 and S9). For example, duplexes 17–20, which contain the highest density of PrLI modifications on both the template and complementary strands, exhibited the lowest susceptibility to *Sma*I and *Xma*I. In contrast, all other duplexes displayed varying degrees of enzyme tolerance, depending on the number and position of the introduced modifications. For instance, duplex 12 exhibited the lowest susceptibility to *Xma*I/*Sma*I cleavage (Figure 2E–G). To evaluate whether this resistance was temperature-dependent, we assessed enzymatic activity at 25 °C and observed no significant differences in the cleavage pattern (Figure S8). Smal and Xmal are isoschizomers, i.e., restriction enzymes that recognize the same DNA sequence, such as CCCGGG, but may not cut at the same location.^47^ *Xma*l cleaves between the external cytosines, while *Sma*l cleaves between CG in the CCCGGG to introduce blunt-end scission.^47^ It has been shown that both endonucleases form a stable, specific complex with DNA. However, the landing of endonuclease on the duplex leads to the bending of DNA such that *Sma*l bends the DNA toward the major groove, similar to *Eco*RI, while *Xma*l bends the DNA toward the minor groove.^47^ This resistance occurs despite the modifications being positioned adjacent to the restriction site, suggesting that downstream modifications may influence the cleavage of upstream restriction sites. A profound loss of sensitivity toward *Xma*I and *Sma*I was observed for duplexes modified with **1b**, **2b**, **3b**, and **4b**, showing that resistivity gradually increased with the increasing number of PpgLI modifications (Figure 2F–2H). These results suggest that the rigidity of the propargyl linker impacted the enzyme recognition site more strongly, even though the modification was not positioned directly on the restriction site. All duplexes 21, 22, and 23, containing propargyl-linked indole, were resistant to DNase I, *Eco*RI, and *Xma*l, despite the degree or site of modification. However, the promiscuity of these duplexes toward *Sma*I slightly deviates from this trend. For example, duplex 23 shows lower resistance to *Sma*I compared to that of duplexes 21 and 22. This observation departs from previous reports showing that the tested restriction enzymes tolerate the presence of 8- or 7-aza-modified purines, even when these modifications are near an R-M recognition site, resulting in efficient scission of the corresponding modified duplexes.^46^

## Discussion

Efforts to broaden the chemical diversity, stability, microenvironment sensitivity, and structural robustness of nucleic acids while preserving their inherent programmability have focused on introducing chemical modifications to natural nucleotides.^48,49^ For example, modifications to the sugar moiety have significantly improved nuclease resistance, while base modifications have expanded the chemical space of nucleic acid ligands, such as those found in SOMAmers and nucleic acid aptamers.^50,51^ These strategies have proven effective in improving ligand specificity, affinity, and chemical stability.^48^ In this study, we synthesized eight PrLI/PpgLI-modified nucleoside phosphoramidites and successfully incorporated them into DNA duplexes. Systematic biochemical evaluation revealed that strategic placement of indole-linked nucleotides connected via a flexible propyl linker could fine-tune the susceptibility of the duplexes to nucleases and restriction enzymes, offering a new approach to modulating sequence recognition and stability. Specifically, we investigated two modifications: one at the C-5 position of pyrimidine and another at the C-8 position of purine in native DNA. Our findings reveal that the rigidity of the linker tethering the bulky substituent at these positions plays a critical role in the conformation and promiscuity of the duplex toward endonucleases. We found that introducing modifications directly at or near a restriction site impacts nuclease cleavage. All duplexes resisted cleavage by DNase I, *Eco*RI, *Sma*I, and *Xma*I when the sense strand was modified with C-5 propargyl indole-linked pyrimidines d­(U, C) and C-8 propargyl indole-linked purines d­(A, G). However, sequence promiscuity varied when the complementary strand was modified with C-5 propyl indole-linked pyrimidines and C-8 propyl indole-linked purines, depending on the position and extent of the modification.

Our understanding of NCC has grown along with a pressing need for molecular tools to expand this knowledge and its applications. While more detailed structural insights into conformational adaptation of the duplexes, coupled with the use of NMR currently underway, our study provides a preliminary molecular framework for inducing noncanonical structures, such as Z-DNA and BZ-DNA, in native DNA strands, bypassing specific sequence composition or high salt concentrations. Several studies have examined the structural features that promote the formation of Z-DNA and BZ junctions. For example, through a series of CD spectral analyses, Vorlickova et al. elegantly reported that poly­(dA-dT) repeats exposed to varying concentrations of CsF undergo a gradual conformational transition from the B-form to a B′ heteronomous form and eventually to BZ and Z-DNA conformations.^36,37,52^ Similarly, a 16-mer duplex containing alternating GC repeats was shown to form BZ junctions under high salt conditions.^17^ These observations are primarily based on unique alternating purine-pyrimidine sequences such as poly­(dG-dC) or poly­(dA-dT), which are known to facilitate such structural transitions. The chimeras (ZImera), one composed of C-5-PrLI pyrimidines and one composed of C-8-PrLI purines, exhibit comparable biochemical behavior characterized by conformational adaptation driven by indole modifications; however, this response appears to be independent of both sequence context and salt concentration. In conclusion, this study demonstrates, for the first time, that complementary strands modified with indole-linked pyrimidines and purines via flexible propyl linkers could be used to target native nucleic acid sequences to induce modified B, Z, or BZ conformations with no sequence dependency and under physiological conditions. Such conformational adaptation controls sequence promiscuity toward digestion enzymes, offering a novel strategy for developing molecular tools to study NCC and create innovative nucleic acid–based therapeutics.

## Supplementary Material



## Notes

Detailed synthetic procedures and corresponding spectroscopic characterizations of intermediates and final phosphoramidites are provided in the Supporting Information (PDF)
