# Supplementary material for: Modification of Deoxyribonucleic Acid with Indole-Linked Nucleotides Induces BZ- and Z‑Conformation and Alters Its Sensitivity to Enzymatic Cleavage
Source: ACS Omega. 2025 Sep 23;10(39):45113–23. doi: 10.1021/acsomega.5c03997 (PMC12509113; doi:10.1021/acsomega.5c03997)
Supplement: Supplementary file 1 [file ao5c03997_si_001.pdf]

Supporting Information

Modification of Deoxyribonucleic Acid with Indole-Linked Nucleotides Induces BZ- and Z-Conformation and Alters Its Sensitivity to Enzymatic Cleavage

Suresh Lingala,[1] Anastasiia Fisiuk,[2] Michelle Stephen,[4] Raja Mohanrao,[1] Judah Klingsberg,[5] Simon Vecchioni,[5] Ealonah S Volvovitz,[4] Sergei Rozhkov,[3] and Prabodhika Mallikaratchy[1,2, 3*]

[1]Department of Molecular, Cellular and Biomedical Sciences, City University of New York School of Medicine, 160 Convent Avenue, New York, NY 10031, USA.

[2]PhD Programs in Chemistry and Biochemistry, Graduate Center, City University of New York, 111 Fifth Avenue, New York, NY 10065, USA.

[3]PhD Program in Biology, Graduate Center, City University of New York, 365 Fifth Avenue, New York, NY 10065, USA.

[4]Department of Biology, City College of New York, 160 Convent Avenue, New York, NY 10031, USA.

[5]Department of Chemistry, New York University, New York, NY 10003, USA.

*Corresponding author: pmallikaratchy@med.cuny.edu

Table content for supporting information

Scheme S1-S4 Synthesis of indole coupled base modified nucleoside phosphoramidites............................S4
Table S1 List of base modified DNA sequences, their calculated and measured masses (Da)......................S24
Figure S1 Melting points and structures of Zimera duplexes... S25
Figure S2 Thermal analysis of ZImera..S26
Figure S3 Analysis of DNase I activity of ZImera..S27
Figure S4 DNase I Assay of Duplexes 12 and 15 at 25°C...S27
Figure S5 Analysis of EcoRI activity of ZImera...S28
Figure S6 EcoRI Assay of Duplexes 12 and 15 at 25°C...S28
Figure S7 Analysis of XmaI activity of ZImera..S29
Figure S8 XmaI Assay of Duplexes 12 and 15 at 25°C..S29
Figure S9 Analysis of SmaI activity of ZImera...S30
Figure S10 ^{1}H NMR spectrum of 3-(prop-2-yn-1-yl)-1*H*-indole (DMSO-*d6*)..S31
Figure S11 ^{13}C NMR spectrum of 3-(prop-2-yn-1-yl)-1*H*-indole (DMSO-*d6*)...S31
Figure S12 ^{1}H NMR spectrum of compound **6** (DMSO-*d6*)..S32
Figure S13 ^{13}C NMR spectrum of compound **6** (DMSO-*d6*)..S32
Figure S14 ^{1}H NMR spectrum of compound **7** (DMSO-*d6*)..S33
Figure S15 ^{13}C NMR spectrum of compound **7** (DMSO-*d6*)..S33
Figure S16 ^{1}H NMR spectrum of compound **8** (DMSO-*d6*)..S34
Figure S17 ^{13}C NMR spectrum of compound **8** (DMSO-*d6*)..S34
Figure S18 ^{1}H NMR spectrum of compound **9** (DMSO-*d6*)..S35
Figure S19 ^{13}C NMR spectrum of compound **9** (DMSO-*d6*)..S35
Figure S20 ^{1}H NMR spectrum of compound **11** (DMSO-*d6*)..S36
Figure S21 ^{13}C NMR spectrum of compound **11** (DMSO-*d6*)..S36
Figure S22 ^{1}H NMR spectrum of compound **12** (DMSO-*d6*)..S37
Figure S23 ^{13}C NMR spectrum of compound **12** (DMSO-*d6*)..S37
Figure S24 ^{1}H NMR spectrum of compound **13** (DMSO-*d6*)..S38
Figure S25 ^{13}C NMR spectrum of compound **13** (DMSO-*d6*)..S38
Figure S26 ^{1}H NMR spectrum of compound **14** (DMSO-*d6*)..S39
Figure S27 ^{13}C NMR spectrum of compound **14** (DMSO-*d6*)..S39
Figure S28 ^{1}H NMR spectrum of compound **15** (DMSO-*d6*)..S40
Figure S29 ^{13}C NMR spectrum of compound **15** (DMSO-*d6*)..S40
Figure S30 ^{1}H NMR spectrum of compound **16** (DMSO-*d6*)..S41
Figure S31 ^{13}C NMR spectrum of compound **16** (DMSO-*d6*)..S41
Figure S32 ^{1}H NMR spectrum of compound **18** (DMSO-*d6*)..S42
Figure S33 ^{13}C NMR spectrum of compound **18** (DMSO-*d6*)..S42
Figure S34 ^{1}H NMR spectrum of compound **19** (DMSO-*d6*)..S43
Figure S35 ^{13}C NMR spectrum of compound **19** (DMSO-*d6*)..S43
Figure S36 ^{1}H NMR spectrum of compound **20** (DMSO-*d6*)..S44
Figure S37 ^{13}C NMR spectrum of compound **20** (DMSO-*d6*)..S44
Figure S38 ^{1}H NMR spectrum of compound **21** (DMSO-*d6*)..S45
Figure S39 ^{13}C NMR spectrum of compound **21** (DMSO-*d6*)..S45
Figure S40 ^{1}H NMR spectrum of compound **22** (DMSO-*d6*)..S46
Figure S41 ^{13}C NMR spectrum of compound **22** (DMSO-*d6*)..S46
Figure S42 ^{1}H NMR spectrum of compound **23** (DMSO-*d6*)..S47
Figure S43 ^{13}C NMR spectrum of compound **23** (DMSO-*d6*)..S47
Figure S44 ^{1}H NMR spectrum of compound **25** (DMSO-*d6*)..S48
Figure S45 ^{13}C NMR spectrum of compound **25** (DMSO-*d6*)..S48

Figure S46 ^{1}H NMR spectrum of compound **26** (DMSO-*d6*)....S49
Figure S47 ^{13}C NMR spectrum of compound **26** (DMSO-*d6*)....S49
Figure S48 ^{1}H NMR spectrum of compound **27** (DMSO-*d6*)....S50
Figure S49 ^{13}C NMR spectrum of compound **27** (DMSO-*d6*)....S50
Figure S50 ^{1}H NMR spectrum of compound **28** ($CDCl_3$)....S51
Figure S51 ^{13}C NMR spectrum of compound **28** ($CDCl_3$)....S51
Figure S52 ^{1}H NMR spectrum of compound **29** (DMSO-*d6*)....S52
Figure S53 ^{13}C NMR spectrum of compound **29** (DMSO-*d6*)....S52
Figure S54 ^{1}H NMR spectrum of compound **30** (DMSO-*d6*)....S53
Figure S55 ^{13}C NMR spectrum of compound **30** (DMSO-*d6*)....S53
Figure S56 ^{1}H NMR spectrum of compound **1a** (CD_3CN)....S54
Figure S57 ^{13}C NMR spectrum of compound **1a** (CD_3CN)....S54
Figure S58 ^{31}P NMR spectrum of compound **1a** (CD_3CN)....S55
Figure S59 ^{1}H NMR spectrum of compound **1b** (CD_3CN)....S55
Figure S60 ^{13}C NMR spectrum of compound **1b** (CD_3CN)....S56
Figure S61 ^{31}P NMR spectrum of compound **1b** (CD_3CN)....S56
Figure S62 ^{1}H NMR spectrum of compound **2a** (CD_3CN)....S57
Figure S63 ^{13}C NMR spectrum of compound **2a** (CD_3CN)....S57
Figure S64 ^{31}P NMR spectrum of compound **2a** (CD_3CN)....S58
Figure S65 ^{1}H NMR spectrum of compound **2b** (CD_3CN)....S58
Figure S66 ^{13}C NMR spectrum of compound **2b** (CD_3CN)....S59
Figure S67 ^{31}P NMR spectrum of compound **2b** (CD_3CN)....S59
Figure S68 ^{1}H NMR spectrum of compound **3a** (CD_3CN)....S60
Figure S69 ^{13}C NMR spectrum of compound **3a** (CD_3CN)....S60
Figure S70 ^{31}P NMR spectrum of compound **3a** (CD_3CN)....S61
Figure S71 ^{1}H NMR spectrum of compound **3b** (CD_3CN)....S61
Figure S72 ^{13}C NMR spectrum of compound **3b** (CD_3CN)....S62
Figure S73 ^{31}P NMR spectrum of compound **3b** (CD_3CN)....S62
Figure S74 ^{1}H NMR spectrum of compound **4a** (CD_3CN)....S63
Figure S75 ^{13}C NMR spectrum of compound **4a** (CD_3CN)....S63
Figure S76 ^{31}P NMR spectrum of compound **4a** (CD_3CN)....S64
Figure S77 ^{1}H NMR spectrum of compound **4b** (CD_3CN)....S64
Figure S78 ^{13}C NMR spectrum of compound **4b** (CD_3CN)....S65
Figure S79 ^{31}P NMR spectrum of compound **4b** (CD_3CN)....S65
Figure S80 Deconvoluted MS spectrum of **Z1**....S66
Figure S81 Deconvoluted MS spectrum of **Z4**....S66
Figure S82 Deconvoluted MS spectrum of **Z5**....S67
Figure S83 Deconvoluted MS spectrum of **Z6**....S67
Figure S84 Deconvoluted MS spectrum of **Z11**....S68
Figure S85 Deconvoluted MS spectrum of **Z44**....S68
Figure S86 Deconvoluted MS spectrum of **Z55**....S69
Figure S87 Deconvoluted MS spectrum of **cZ3**....S69
Figure S88 Deconvoluted MS spectrum of **cZ4**....S70
Figure S89 Deconvoluted MS spectrum of **cZ5**....S70
References....S70

1. Synthesis of indole coupled base modified nucleoside phosphoramidites:

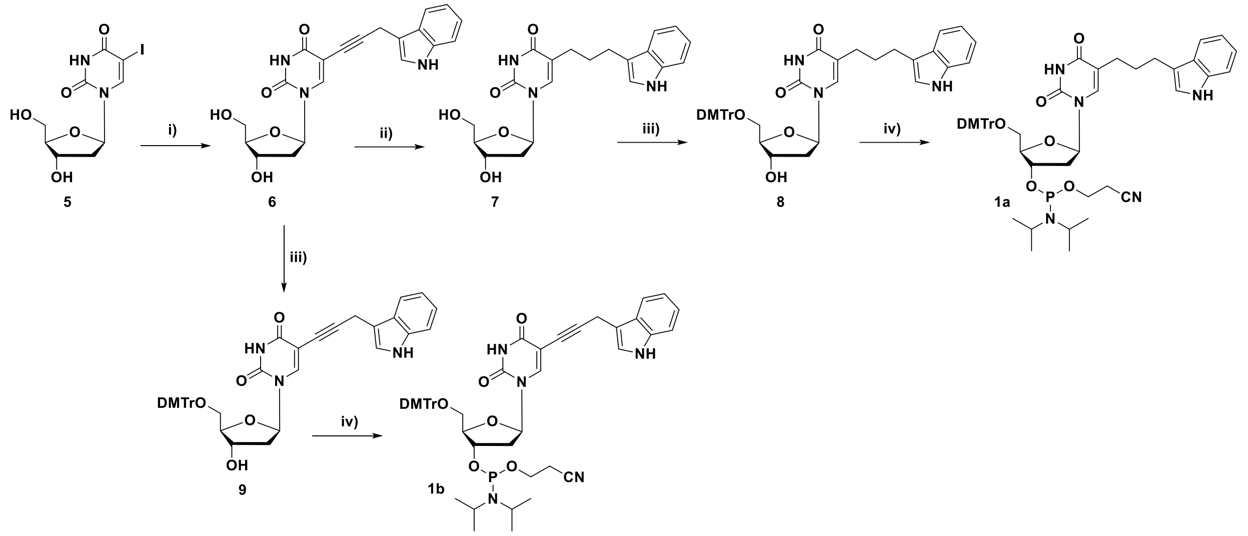

Scheme S1: (i) $Pd(PPh_3)_4$, CuI, Et_3N, 1*H*-Indole,3-(2-propynyl), DMF, 55 °C 2 h, 71% (6) (ii) H_2, 10% Pd/C, MeOH, 50 °C, 12 h, 74% (7) (iii) DMTrCl, DMAP, pyridine, rt, 12 h, 50% (8), 53% (9) (iv) 2-cyanoethyl-*N*,*N*-diisopropylchlorophosphoramidite, DIPEA, DCM, 0 °C to rt, 1.0 h, 68% (1a), 58% (1b).

Synthesis of 5-(3-(1*H*-indol-3-yl)prop-1-yn-1-yl)-1-((2R,4S,5R)-4-hydroxy-5 (hydroxymethyl)tetrahydrofuran-2-yl)pyrimidine-2,4(1*H*,3*H*)-dione (6):

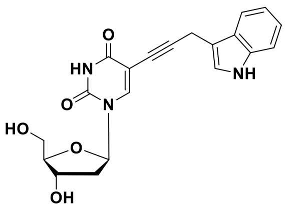

To a solution of 5-iododeoxyuridine (**5**) (3.0 g, 8.50 mmol) and 3-(prop-2-yn-1-yl)-1*H*-indole[1] (1.7 g, 11.0 mmol) were added to a mixture of $Pd(PPh_3)4$ (0.98 g, 0.85 mmol) and CuI (0.48 g, 2.50 mmol) in anhydrous DMF (30 mL) under a Ar atmosphere in a flask equipped with a gas inlet tube and a magnetic stirrer. Then pump/purge cycles were applied with the addition of Ar gas Et_3N (5.90 mL, 42.0 mmol) was added and then the mixture was heated at 55 °C for 2 h. The progress of the reaction was followed using TLC. After completion of the reaction, evaporated under reduced pressure and the crude product was purified by flash chromatography (0–10% MeOH in CH_2Cl_2). The desired compound **6** (2.3 g, 6.0 mmol, 71%) was obtained as a white solid.

$\mathbf{R_f}$ (DCM/Methanol 9:1, v/v) 0.45.

^{1}H NMR (500 MHz, DMSO-*d$_6$*) δ_H 11.58 (s, 1H), 10.90 (s, 1H), 8.16 (s, 1H), 7.60 (d, *J* = 7.8 Hz, 1H), 7.36 (d, *J* = 8.1 Hz, 1H), 7.29 (d, *J* = 1.1 Hz, 1H), 7.09 (dd, *J* = 8.0, 7.1 Hz, 1H), 7.00 (t, *J* = 7.4 Hz, 1H), 6.12 (t, *J* = 6.7 Hz, 1H), 5.25 (d, *J* = 4.3 Hz, 1H), 5.11 (t, *J* = 5.0 Hz, 1H), 4.26 – 4.21 (m, 1H), 3.85 (s, 2H), 3.80 (app q, J_{app} ~ *J* = 3.0 Hz, 1H), 3.66 – 3.53 (m, 2H), 2.18 – 2.06 (m, 2H).

^{13}C NMR (126 MHz, DMSO-*d$_6$*) δ_C 162.3, 149.9, 143.7, 136.8, 126.9, 123.5, 121.6, 118.9, 118.9 111.9, 109.8, 99.5, 92.1, 88.1, 85.1, 73.6, 70.7, 61.5, 40.5, 16.2.

HRMS ESI/Q-TOF $[M+Na]^+$ calcd. for $C_{20}H_{19}N_3NaO_5$ 404.1217, found 404.1220.

Synthesis of 5-(3-(1*H*-indol-3-yl)propyl)-1-((2R,4S,5R)-4-hydroxy-5-(hydroxymethyl)tetrahydrofuran-2-yl)pyrimidine-2,4(1*H*,3*H*)-dione (7):

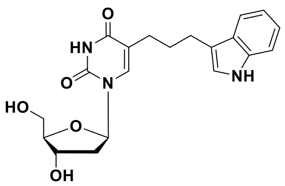

To a solution of compound **6** (2.0 g, 5.2 mmol) in methanol (40 mL), 10% Pd/C (0.56 g, 0.52 mmol) was added under stirring. The reaction mixture was then stirred under hydrogen atmosphere at 50 °C for 12 h. After completion of the reaction, the reaction mixture was filtered through celite to remove the catalyst. The filtrate was evaporated under reduced pressure to yield the pure compound **7** (1.5 g, 3.9 mmol, 74 %) as a white solid.

R_f (DCM/Methanol 9:1,v/v) 0.4.

^{1}H NMR (500 MHz, DMSO-d_6) δ_H 11.26 (s, 1H), 10.73 (s, 1H), 7.72 (s, 1H), 7.48 (d, *J* = 7.8 Hz, 1H), 7.32 (d, *J* = 8.1 Hz, 1H), 7.12 (s, 1H), 7.05 (t, *J* = 7.4 Hz, 1H), 6.96 (t, *J* = 7.3 Hz, 1H), 6.18 (t, *J* = 6.8 Hz, 1H), 5.23 (d, *J* = 3.8 Hz, 1H), 5.04 (t, *J* = 4.8 Hz, 1H), 4.25 (bs, 1H), 3.78 (app q, J_{app} ~ 2.6 Hz, 1H), 3.63 – 3.51 (m, 2H), 2.68 (t, *J* = 7.2 Hz, 2H), 2.34 – 2.24 (m, 2H), 2.16 – 2.03 (m, 2H), 1.88 – 1.76 (m, 2H).

^{13}C NMR (126 MHz, DMSO-d_6) δ_C 163.9, 150.8, 136.7, 136.6, 127.6, 122.6, 121.2, 118.7, 118.5, 114.6, 114.0, 111.8, 87.8, 84.4, 70.9, 61.8, 46.1, 28.9, 26.8, 24.8.

HRMS (ESI/Q-TOF) $[M+Na]^+$ calcd. for $C_{20}H_{23}N_3NaO_5$ 408.1530, found 408.1544.

Synthesis of 5-(3-(1*H*-indol-3-yl)propyl)-1-((2R,4S,5R)-5-((bis(4-methoxyphenyl)(phenyl)methoxy)methyl)-4-hydroxytetrahydrofuran-2-yl)pyrimidine-2,4(1*H*,3*H*)-dione (8):

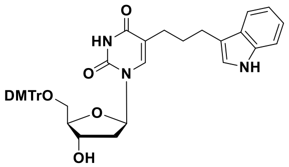

To a solution of compound **7** (1.0 g, 2.6 mmol) in dry pyridine (15 mL), DMAP (32 mg, 0.26 mmol) was added. To this mixture, a solution of 4,4’-dimethoxytrityl chloride (1.1 g, 3.1 mmol) in 5 mL of anhydrous pyridine was added in four equal portions over the time of 1 h. The reaction mixture was stirred at room temperature for 12 h. After completion of the reaction, the solvent was evaporated under reduced pressure. The resulting crude mixture was purified by flash chromatography (0–2% MeOH in CH_2Cl_2 with 0.2% of Et_3N) yielding the desired compound **8** (0.90 g, 1.31 mmol, 50%) as a yellow foam.

R_f (DCM/Methanol 9:1, v/v) 0.5.

^{1}H NMR (500 MHz, DMSO-d_6) δ_H 11.32 (s, 1H), 10.71 (s, 1H), 7.44 (s, 1H), 7.39 (d, *J* = 7.9 Hz, 2H), 7.34 (d, *J* = 7.9 Hz, 1H), 7.30 (m 3H), 7.25 (m 4H), 7.21 (t, *J* = 7.3 Hz, 1H), 7.04 (t, *J* = 7.6 Hz, 1H), 6.98 (s, 1H), 6.91 (dd, *J* = 17.6, 9.9 Hz, 1H), 6.85 (m, 4H), 6.21 (t, *J* = 6.8 Hz, 1H), 5.34 (d, *J* = 4.5 Hz, 1H), 4.32 (app q, J_{app} ~ 3.1 Hz, 1H), 3.88 (app q, J_{app} ~ 6.8 Hz, 1H), 3.68 (d, *J* = 3.0 Hz, 6H), 3.18 (m, 2H), 2.44 (t, *J* = 7.6 Hz, 2H), 2.26 (dt, *J* = 11.5, 6.9 Hz, 1H), 2.21 – 2.12 (m, 1H), 2.07 – 1.97 (m, 1H), 1.90 (dt, *J* = 11.1, 7.5 Hz, 1H), 1.68 – 1.53 (m, 2H).

^{13}C NMR (126 MHz, DMSO-d_6) δ_C 163.7, 158.6, 150.7, 150.0, 145.1, 136.7, 136.1, 135.9, 135.7, 130.2, 130.1, 128.3, 128.6, 127.6, 127.3, 122.4, 121.2, 118.6, 118.5, 114.6, 114.5, 113.7, 111.7, 86.3, 85.9, 84.3, 71.1, 64.2, 55.4, 49.1, 29.5, 27.1, 24.9.

HRMS (ESI/Q-TOF) $[M+Na]^+$ calcd. for $C_{41}H_{41}N_3NaO_7$ 710.2837, found 710.2840.

Synthesis of (2R,3S,5R)-5-(5-(3-(1*H*-indol-3-yl)propyl)-2,4-dioxo-3,4-dihydropyrimidin-1(2*H*)-yl)-2-((bis(4-methoxyphenyl)(phenyl)methoxy)methyl)tetrahydrofuran-3-yl (2-cyanoethyl) diisopropylphosphoramidite (1a):

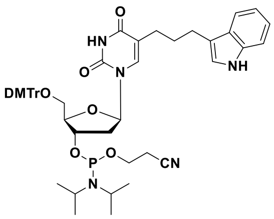

To a solution of compound **8** (0.6 g, 0.87 mmol) in anhydrous CH_2Cl_2, *N*,*N*-diisopropylethylamine (0.75 mL, 4.36 mmol) was added under stirring. The reaction mixture was cooled to 0 °C, and 2-cyanoethyl-*N*,*N*-diisopropylchlorophosphoramidite (0.3 mL, 1.40 mmol) was added under an argon atmosphere. The mixture was allowed to warm to room temperature and stirred for 1.0 h. The reaction mixture was then diluted with anhydrous CH_2Cl_2 (30 mL) and washed successively with 5% $NaHCO_3$ (30 mL) and brine solution (30 mL). The organic phase was dried over anhydrous Na_2SO_4 and concentrated under reduced pressure. The crude product was purified by column chromatography using (10-60% EtOAc in hexane containing 0.5% Et_3N) as the eluent. The desired compound **1a** was obtained as a 1:1.3 mixture of two diastereomers in the form of a white foam (0.52 g, 0.6 mmol, 68% yield).

$\mathbf{R_f}$ (EtOAc/Hexane 8:2, v/v) 0.50, 0.58.

^{1}**H NMR** (500 MHz, CD_3CN) δ_H 8.53 (s, 1H), 7.85 (s, 1H), 7.52 (m, 1H), 7.44 (m, 3H), 7.31 (m, 7H), 7.24 (m, 1H), 7.15 (t, *J* = 7.6 Hz, 1H), 7.05 (t, *J* = 7.4 Hz, 1H), 6.82 (dd, *J* = 8.0, 5.8 Hz, 4H), 6.75 (d, *J* = 3.0 Hz, 1H), 6.40 (q, *J* = 7.4 Hz, 1H), 4.70 – 4.59 (m, 1H), 4.23 – 4.11 (m, 3H), 3.90 (dd, *J* = 11.0, 5.8 Hz, 1H), 3.72 (d, *J* = 6.1 Hz, 6H), 3.67 – 3.46 (m, 4H), 3.31 (td, *J* = 10.1, 2.5 Hz, 1H), 2.62 (q, *J* = 6.4 Hz, 1H), 2.57 – 2.47 (m, 2H), 2.43 (dd, *J* = 12.1, 8.8 Hz, 1H), 2.28 (dt, *J* = 12.2, 7.0 Hz, 1H), 2.18 – 2.10 (m, 1H), 1.98 – 1.86 (m, 1H), 1.76 (ddd, *J* = 9.6, 8.9, 4.0 Hz, 1H), 1.2-1.7 (m, 9H), 1.07 (d, *J* = 10 Hz, 3H).

^{13}**C NMR** (126 MHz, CD_3CN) δ_C δ 163.49, 158.79, 150.46, 144.89, 136.55, 135.83, 135.79, 135.78, 135.75, 135.62, 135.57, 130.18, 130.15, 130.13, 130.10, 128.16, 128.10, 127.98, 127.51, 127.05, 127.03, 121.83, 121.30, 118.59, 118.52, 118.42, 115.16, 114.87, 114.82, 113.19, 111.19, 86.46, 86.44, 85.06, 85.02, 84.81, 84.76, 84.40, 84.38, 73.41, 73.27, 72.99, 72.86, 63.27, 63.11, 60.03, 58.58, 58.43, 57.20, 54.94, 54.92, 43.13, 43.03, 39.09, 29.12, 29.09, 26.85, 26.83, 24.48, 23.99, 23.98, 23.93, 23.87, 21.03, 20.22, 20.11, 20.05, 20.03, 19.97, 13.59.

^{31}**P NMR** (202 MHz, CD_3CN) δ_P 148.58, 148.55.

HRMS (ESI/Q-TOF) $[M+Na]^+$ calcd. for $C_{50}H_{58}N_5NaO_8P$ 910.3915, found 910.3795.

Synthesis of 5-(3-(1*H*-indol-3-yl)prop-1-yn-1-yl)-1-((2R,4S,5R)-5-((bis(4-methoxyphenyl)(phenyl)methoxy)methyl)-4-hydroxytetrahydrofuran-2-yl)pyrimidine-2,4(1*H*,3*H*)-dione (9):

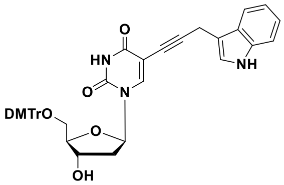

To a solution of compound **6** (1.0 g, 2.6 mmol) in dry pyridine (15 mL), DMAP (32 mg, 0.26 mmol) was added under stirring. To this mixture, a solution of 4,4'-dimethoxytrityl chloride (1.1 g, 3.1 mmol) in 5 mL of anhydrous pyridine was added in four equal portions over the time of 1 h. The reaction mixture was stirred at room temperature for 12 h. After completion of the reaction, the solvent was evaporated under reduced pressure. The resulting crude mixture was purified by flash

chromatography (0–2% MeOH in CH_2Cl_2, with 0.2% of Et_3N) yielding the desired compound **9** (0.95 g, 1.39 mmol, 53%) as a yellowish foam.

$\mathbf{R_f}$ (DCM/Methanol 9:1, v/v) 0.5.

^{1}H NMR (500 MHz, DMSO-*d$_6$*) δ_H 11.64 (s, 1H), 10.88 (s, 1H), 7.90 (s, 1H), 7.53 (d, *J* = 7.9 Hz, 1H), 7.42 (d, *J* = 7.7 Hz, 2H), 7.36 (d, *J* = 8.1 Hz, 1H), 7.32 – 7.28 (m, 6H), 7.20 (d, *J* = 4.7 Hz, 2H), 7.09 (t, *J* = 7.5 Hz, 1H), 6.96 (t, *J* = 7.4 Hz, 1H), 6.86 (dd, *J* = 8.7, 4.0 Hz, 4H), 6.13 (t, *J* = 6.6 Hz, 1H), 5.35 (d, *J* = 4.4 Hz, 1H), 4.29 (app q, J_{app} ~ 4.4 Hz, 1H), 3.93 (app q, J_{app} ~ 5.0 Hz, 1H), 3.69 (s, 6H), 3.66 (s, 2H), 3.27 – 3.24 (m, 1H), 3.09 (dd, *J* = 5.0, 1.9 Hz, 1H), 2.28 (dd, *J* = 12.4, 6.7 Hz, 1H), 2.23 – 2.13 (m, 1H).

^{13}C NMR (126 MHz, DMSO-*d$_6$*) δ_C 162.3, 158.5, 149.8, 145.3, 142.6, 136.9,136.1, 135.8, 130.2, 130.1, 128.4, 127.1, 123.4, 121.6, 118.9, 118.8, 113.7, 113.6, 112.0, 109.6, 99.7, 92.2, 86.3, 86.2, 85.4, 73.1, 71.0, 64.2, 55.5, 16.1.

HRMS (ESI/Q-TOF) $[M+Na]^+$ calcd. for $C_{41}H_{37}N_3NaO_7$ 706.2529, found 707.2556.

Synthesis of (2R,3S,5R)-5-(5-(3-(1*H*-indol-3-yl)prop-1-yn-1-yl)-2,4-dioxo-3,4-dihydropyrimidin-1(2*H*)-yl)-2-((bis(4-methoxyphenyl)(phenyl)methoxy)methyl)tetrahydrofuran-3-yl (2-cyanoethyl) diisopropylphosphoramidite (1b):

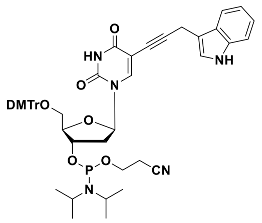

To a solution of compound **9** (0.5 g, 0.73 mmol) in anhydrous CH_2Cl_2, *N*,*N*-diisopropylethylamine (0.63 mL, 3.66 mmol) was added under stirring. The reaction mixture was cooled to 0 °C, and 2-cyanoethyl-*N*,*N*-diisopropylchlorophosphoramidite (0.25 mL, 1.2 mmol) was added under an argon atmosphere. The mixture was allowed to warm to room temperature and stirred for 1.0 h. The reaction mixture was then diluted with anhydrous CH_2Cl_2 (30 mL) and washed successively with 5% $NaHCO_3$ (30 mL) and brine solution (30 mL). The organic phase was dried over anhydrous Na_2SO_4 and concentrated under reduced pressure. The crude product was purified by column chromatography using (10-50% EtOAc in hexane containing 0.5% Et_3N) as the eluent. The desired compound **1b** was obtained as a 1:1.2 mixture of two diastereomers in the form of a white foam (0.37 g, 0.43 mmol, 58% yield).

$\mathbf{R_f}$ (EtOAc/Hexane 8:2, v/v) 0.45, 0.50.

^{1}H NMR (500 MHz, CD_3CN) δ_H 9.16 (s, 1H), 9.09 (s, 1H), 7.95 (d, *J* = 13.5 Hz, 1H), 7.56 (d, *J* = 7.9 Hz, 1H), 7.54 – 7.50 (m, 1H), 7.46 – 7.38 (m, 5H), 7.34 (td, *J* = 7.8, 2.5 Hz, 2H), 7.28 – 7.22 (m, 1H), 7.21 – 7.15 (m, 1H), 7.13 – 7.09 (m, 1H), 7.09 – 7.04 (m, 1H), 6.87 (m, 4H), 6.20 (m, 1H), 4.68 (m, 1H), 4.24 – 4.13 (m, 1H), 3.82 (m, 1H), 3.75 (s, 3H), 3.74 (s, 3H), 3.72 (dd, *J* = 7.9, 5.7 Hz, 1H), 3.68 – 3.58 (m, 4H), 3.40 – 3.27 (m, 2H), 2.69 (t, *J* = 6.0 Hz, 1H), 2.58 (td, *J* = 6.0, 2.3 Hz, 3H), 2.48 – 2.37 (m, 1H), 1.23-1.20 (m, 9H), 1.12 (d, *J* = 5 Hz, 3H).

^{13}C NMR (126 MHz, CD_3CN) δ_C 161.85, 161.82, 158.75, 149.44, 145.01, 144.99, 142.06, 142.03, 136.65, 135.86, 135.82, 135.57, 135.52, 130.18, 130.15, 130.10, 130.07, 128.01, 127.96, 127.92, 126.91, 126.65, 122.75, 121.69, 118.95, 118.42, 113.19, 113.17, 111.40, 110.07, 99.92, 99.83, 91.96, 91.93, 86.66, 86.64, 85.56, 85.53, 85.44, 85.36, 85.31, 85.27, 73.32, 72.99, 72.86, 72.42, 72.40, 63.23, 62.97, 60.01, 58.62, 58.47, 58.25, 58.21, 54.92, 54.91, 45.07, 45.02, 43.13, 43.03, 39.84, 39.69, 23.98, 23.95, 23.92, 23.90, 23.86, 22.23, 22.21, 22.16, 22.15, 20.20, 20.10, 20.05, 19.99, 19.67, 19.61, 15.63, 13.56.

^{31}P NMR (202 MHz, CD_3CN) δ_P 148.12, 148.02.

HRMS (ESI/Q-TOF) $[M+H]^+$ calcd. for $C_{50}H_{55}N_5O_8P$ 884.3783, found 884.3750.

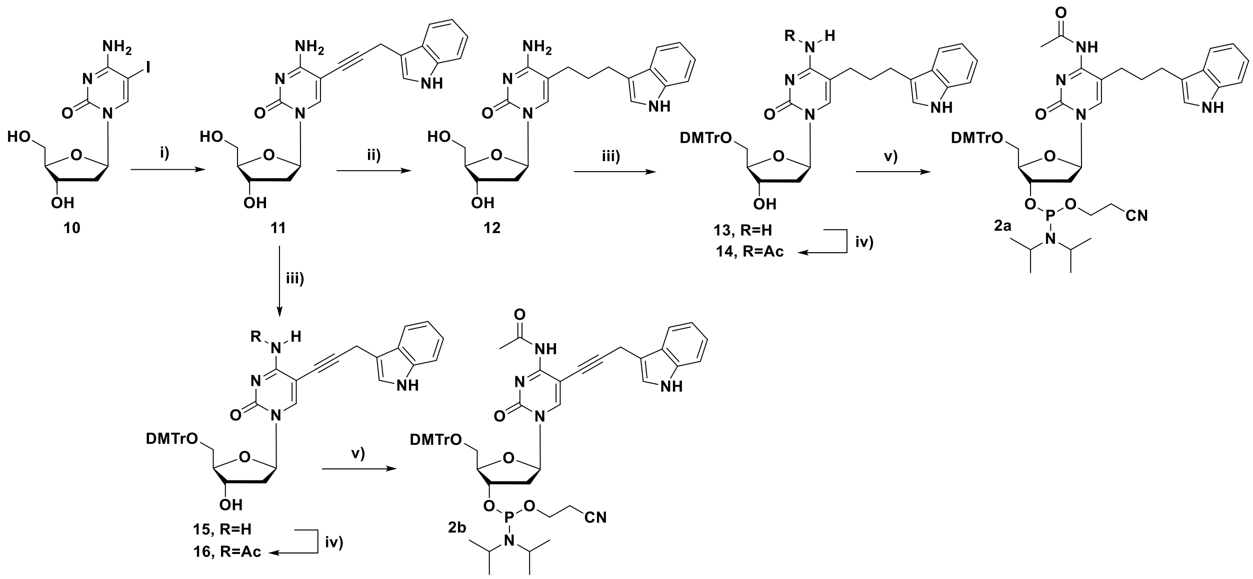

Scheme S2: (i) $Pd(PPh_3)_4$, CuI, Et_3N, 1*H*-Indole,3-(2-propynyl), DMF, 55 °C 1.5 h, 70% (11) (ii) H_2, 10% Pd/C, MeOH, 50 °C, 12 h, 84% (12) (iii) DMTrCl, DMAP, pyridine, rt, 12 h, 54% (13), 56% (15) (iv) Ac_2O, DMF, rt, 8-10 h, 61% (14), 57% (16) (v) 2-cyanoethyl-*N*,*N*-diisopropylchlorophosphoramidite, DIPEA, DCM, 0 °C to rt, 1.5 h, 56% (2a), 64% (2b).

Synthesis of 5-(3-(1*H*-indol-3-yl)prop-1-yn-1-yl)-4-amino-1-((2R,4S,5R)-4-hydroxy-5-(hydroxymethyl)tetrahydrofuran-2-yl)pyrimidin-2(1*H*)-one (11):

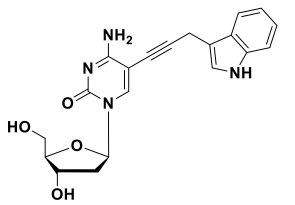

To a solution of 5-iodo-2'-deoxycytidine (10) (2.0 g, 5.66 mmol) and 3-(prop-2-yn-1-yl)-1*H*-indole[1] (1.14 g, 7.36 mmol) were added to a mixture of Pd(PPh3)4 (0.66 g, 0.57 mmol) and CuI (0.32 g, 1.70 mmol) in anhydrous DMF (30 mL) under a Ar atmosphere in a flask equipped with a gas inlet tube and a magnetic stirrer. Then pump/purge cycles were applied with the addition of Ar gas Et_3N (3.95 mL, 28.3 mmol) was added and then the mixture was heated at 55 °C for 1.5 h. The progress of the reaction was followed using TLC. After completion of the reaction, evaporated under reduced pressure and the crude product was purified by flash chromatography (0–10% MeOH in CH_2Cl_2). The desired compound 11 (1.5 g, 3.9 mmol, 70%) was obtained as a white solid.

R_f (DCM/Methanol 9:1, v/v) 0.4.

^{1}H NMR (500 MHz, DMSO-d_6) δ_H 10.09 (s, 1H), 7.29 (s, 1H), 6.90 (br.s, 2H), 6.83 (d, *J* = 7.8 Hz, 1H), 6.55 (d, *J* = 8.1 Hz, 1H), 6.46 (s, 1H), 6.28 (t, *J* = 7.4 Hz, 1H), 6.19 (t, *J* = 7.4 Hz, 1H), 5.97 (s, 1H), 5.31 (t, *J* = 6.5 Hz, 1H), 4.38 (d, *J* = 4.0 Hz, 1H), 4.23 (t, *J* = 4.9 Hz, 1H), 3.35 (br, s, 1H) 3.05 (s, 2H), 2.93 (app q, J_{app} ~ 2.9 Hz, 1H), 2.84 – 2.69 (m, 2H), 1.69 (s, 1H), 1.36 – 1.27 (m, 1H), 1.17 (dt, *J* = 12.1, 6.5 Hz, 1H).

^{13}C NMR (126 MHz, DMSO-*d$_6$*) δ_C 167.1, 156.2, 146.3, 139.0, 129.1, 125.7, 123.9, 121.1, 121.1 114.2, 112.0, 97.0, 93.0, 90.1, 87.9, 74.8, 72.8, 63.7, 43.4, 18.5.

HRMS (ESI/Q-TOF) $[M+H]^+$ calcd. for $C_{20}H_{21}N_4O_4$ 381.1563, found 381.1542.

Synthesis of 5-(3-(1*H*-indol-3-yl)propyl)-4-amino-1-((2R,4S,5R)-4-hydroxy-5 (hydroxymethyl)tetrahydrofuran-2-yl)pyrimidin-2(1*H*)-one (12):

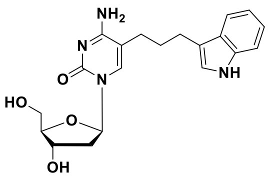

To a solution of compound **11** (1.3 g, 3.4 mmol) in methanol (40 mL), 10% Pd/C (0.36 g, 0.34 mmol) was added under stirring. The reaction mixture was then stirred under a hydrogen atmosphere at 50 °C for 12 h. The progress of the reaction was followed using TLC. After completion of the reaction, the reaction mixture was filtered through celite to remove the catalyst. The filtrate was evaporated under reduced pressure to yield the pure compound **12** (1.1 g, 2.9 mmol, 84 %) as a white solid.

R$_f$ (DCM/Methanol 8:2, v/v) 0.4.

^{1}H NMR (500 MHz, DMSO-*d$_6$*) δ_H 10.74 (s, 1H), 7.68 (s, 1H), 7.50 (d, *J* = 7.8 Hz, 1H), 7.32 (d, *J* = 8.1 Hz, 1H), 7.29 (s, 1H), 7.11 (d, *J* = 1.7 Hz, 1H), 7.05 (t, *J* = 7.5 Hz, 1H), 6.96 (t, *J* = 7.4 Hz, 1H), 6.93 (s, 1H), 6.18 (t, *J* = 6.7 Hz, 1H), 5.18 (d, *J* = 4.2 Hz, 1H), 5.01 (t, *J* = 5.2 Hz, 1H), 4.22 (dt, *J* = 6.7, 3.4 Hz, 1H), 3.77 (app q, J_{app} ~ 3.5 Hz, 1H), 3.64 – 3.50 (m, 2H), 2.72 (t, *J* = 5.0 Hz, 2H), 2.41 – 2.30 (m, 2H), 2.09 (ddd, *J* = 12.0, 5.9, 3.3 Hz, 1H), 1.99 (dt, *J* = 12.2, 6.6 Hz, 1H), 1.85 – 1.76 (m, 2H).

^{13}C NMR (126 MHz, DMSO-*d$_6$*) δ_C 165.0, 155.2, 138.8, 136.8, 127.6, 122.6, 121.3, 118.8, 118.5, 114.7, 111.8, 105.9, 87.7, 85.4, 70.9, 61.8, 40.8, 29.0, 27.2, 24.7.

HRMS (ESI/Q-TOF) $[M+H]^+$ calcd. for $C_{20}H_{25}N_4O_4$ 385.1876, found 385.1864.

Synthesis of 5-(3-(1*H*-indol-3-yl)propyl)-4-amino-1-((2R,4S,5R)-5-((bis(4 methoxyphenyl)(phenyl)methoxy)methyl)-4-hydroxytetrahydrofuran-2-yl)pyrimidin-2(1*H*)-one (13):

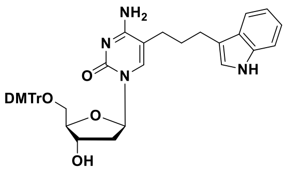

To a solution of compound **12** (1.0 g, 2.6 mmol) in dry pyridine (15 mL), DMAP (32 mg, 0.26 mmol) was added. To this mixture, a solution of 4,4'-dimethoxytrityl chloride (1.1 g, 3.1 mmol) in 5 mL of anhydrous pyridine was added in four equal portions over the time of 1 h. The reaction mixture was stirred at room temperature for 12 h. After completion of the reaction, the solvent was evaporated under reduced pressure. The resulting crude mixture was purified by flash chromatography using (0–2% MeOH in CH_2Cl_2, with 0.2% of Et_3N) yielding the desired compound **13** (0.97 g, 1.41 mmol, 54%) as a yellowish foam.

R$_f$ (DCM/Methanol 9:1, v/v) 0.5.

^{1}H NMR (500 MHz, DMSO-*d$_6$*) δ_H 10.70 (s, 1H), 7.42 (s, 1H), 7.39 (d, *J* = 7.5 Hz, 3H), 7.33 – 7.22 (m, 8H), 7.19 (t, *J* = 7.1 Hz, 1H), 7.04 (t, *J* = 7.4 Hz, 1H), 6.95 (s, 1H), 6.92 (t, *J* = 7.5 Hz, 1H), 6.83 (d, *J* = 7.0 Hz, 4H), 6.24 (t, *J* = 6.6 Hz, 1H), 5.27 (d, *J* = 4.3 Hz, 1H), 4.28 (bs, 1H), 3.88 (app q, J_{app} ~ 3.4 Hz, 1H), 3.67 (s, 3H), 3.66 (s, 3H), 3.17 (s, 2H), 2.47 – 2.39 (m, 2H), 2.20 – 2.05 (m, 3H), 2.02 – 1.92 (m, 1H), 1.70 – 1.49 (m, 2H).

^{13}C NMR (126 MHz, DMSO-*d$_6$*) δ_C 165.2, 158.6, 158.6, 155.3, 155.3, 145.2, 138.0, 136.7, 135.97, 135.7, 130.2, 130.2, 128.3, 128.1, 127.5, 127.2, 122.3, 121.2, 118.7, 118.5, 114.8, 113.7, 111.7, 106.4, 86.2, 85.7, 84.9, 71.1, 64.2, 55.4, 40.9, 29.7, 27.5, 24.8.

HRMS (ESI/Q-TOF) $[M+H]^+$ calcd. for $C_{41}H_{43}N_4O_6$ 687.3183, found 687.3166.

Synthesis of N-(5-(3-(1*H*-indol-3-yl)propyl)-1-((2R,4S,5R)-5-((bis(4-methoxyphenyl)(phenyl)methoxy)methyl)-4-hydroxytetrahydrofuran-2-yl)-2-oxo-1,2-dihydropyrimidin-4-yl)acetamide (14):

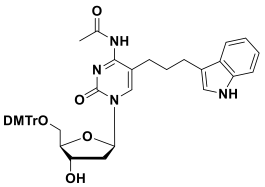

To a solution of compound **13** (0.85g, 1.24 mmol) in dry DMF (12 mL), acetic anhydride (0.13 mL, 1.36 mmol) was added dropwise over 15 minutes at 0 °C under an argon atmosphere. The reaction mixture was then stirred at room temperature for 8 h. Progress of the reaction was monitored by TLC. After completion of the reaction, the reaction mixture was concentrated under reduced pressure. The crude product was purified by flash chromatography using (0–2% MeOH in CH_2Cl_2, with 0.2% of Et_3N) yielding the desired compound **14** (0.55 g, 0.76 mmol, 61%) as a pale-yellow solid.

R$_f$ (DCM/Methanol 9:1, v/v) 0.55.

^{1}H NMR (500 MHz, DMSO-*d$_6$*) δ_H 10.72 (s, 1H), 9.84 (s, 1H), 7.84 (s, 1H), 7.37 (t, *J* = 9.2 Hz, 3H), 7.30 (dd, *J* = 12.2, 7.9 Hz, 3H), 7.22 (m, 4H), 7.20 (d, *J* = 7.3 Hz, 1H), 7.04 (t, *J* = 7.4 Hz, 1H), 6.96 – 6.93 (m, 1H), 6.91 (d, *J* = 7.3 Hz, 1H), 6.84 (d, *J* = 8.5 Hz, 4H), 6.15 (t, *J* = 6.2 Hz, 1H), 5.34 (d, *J* = 4.6 Hz, 1H), 4.30 – 4.24 (m, 1H), 3.97 (dt, *J* = 7.6, 3.9 Hz, 1H), 3.68 (s, 3H), 3.66 (s, 3H) 3.26 – 3.17 (m, 2H), 2.45 – 2.41 (m, 2H), 2.36 – 2.30 (m, 2H), 2.18 (s, 3H), 2.16 – 2.08 (m, 2H), 1.64 – 1.51 (m, 2H).

^{13}C NMR (126 MHz, DMSO-*d$_6$*) δ_C 171.1, 162.3, 158.6, 154.3, 145.1, 142.2, 142.1, 136.7, 136.0, 135.7, 130.2, 130.2, 128.4, 128.2, 127.5, 127.3, 122.4, 121.3, 118.6, 118.5, 114.5, 113.7, 111.8, 110.6, 86.4, 86.3, 70.6, 63.8, 55.5, 41.2, 29.8, 27.6, 24.8.

HRMS (ESI/Q-TOF) $[M+H]^+$ calcd. for $C_{43}H_{45}N_4O_7$ 729.3288, found 729.3376.

Synthesis of (2R,3S,5R)-5-(5-(3-(1*H*-indol-3-yl)propyl)-4-acetamido-2-oxopyrimidin-1(2*H*)-yl)-2-(hydroxymethyl)tetrahydrofuran-3-yl (2-cyanoethyl) diisopropylphosphoramidite (2a):

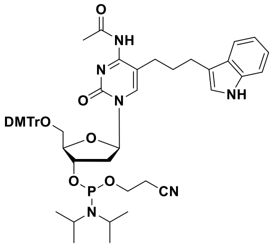

To a solution of compound **14** (0.4 g, 0.55 mmol) in anhydrous CH_2Cl_2, *N,N*-diisopropylethylamine (0.47 mL, 2.74 mmol) was added under stirring. The reaction mixture was cooled to 0 °C, and 2-cyanoethyl-*N,N*-diisopropylchlorophosphoramidite (0.19 mL, 0.88 mmol) was added under an argon atmosphere. The mixture was allowed to warm to room temperature and stirred for 1.5 h. The reaction mixture was then diluted with anhydrous CH_2Cl_2 (20 mL) and washed successively with 5% $NaHCO_3$ (20 mL) and brine solution (20 mL). The organic phase was dried over anhydrous Na_2SO_4 and concentrated under reduced pressure. The crude product was purified by column chromatography using (10-70% EtOAc in hexane containing 0.5% Et_3N) as the eluent. The desired compound **2a** was obtained as a 1:1.4 mixture of two diastereomers in the form of a white foam (0.28 g, 0.31 mmol, 56% yield).

R$_f$ (EtOAc/Hexane 9:1, v/v) 0.5, 0.55.

^{1}H NMR (500 MHz, CD_3CN) δ 8.97 (s, 1H), 7.92 – 7.56 (m, 1H), 7.44 (t, *J* = 11.5 Hz, 3H), 7.35 (d, *J* = 8.2 Hz, 1H), 7.31 (t, *J* = 10.0 Hz, 6H), 7.23 (t, *J* = 7.3 Hz, 1H), 7.09 (t, *J* = 7.5 Hz, 1H), 6.98 (t, *J* = 7.4 Hz, 1H), 6.92 (s, 1H), 6.83 (d, *J* = 8.2 Hz, 5H), 6.25 – 6.13 (m, 1H), 4.73 – 4.50 (m, 1H), 4.16 (s, 1H), 3.71 (s, 6H), 3.66 – 3.54 (m, 4H), 3.40 (d, *J* = 9.8 Hz, 1H), 3.30 (dd, *J* = 10.8, 3.9 Hz, 1H), 2.52 (dt, *J* = 12.3, 6.1 Hz, 6H), 2.44 – 2.34 (m, 1H), 2.27 (s, 2H), 2.23 – 2.17 (m, 1H), 2.11 (s, 1H), 1.85 – 1.55 (m, 2H), 1.13-1.17 (m, 12H).

^{13}C NMR (126 MHz, CD_3CN) δ 173.4, 161.5, 147.6, 139.3, 138.5, 138.3, 132.9, 132.8, 130.9, 130.7, 130.2, 130.2, 129.8, 124.6, 124.6, 124.1, 121.3, 121.2, 121.1, 115.9, 113.9, 89.2, 88.1, 88.1, 75.3, 75.2, 75.2, 65.6, 65.6, 65.5, 62.7, 61.3, 61.2, 59.9, 57.7, 45.8, 45.7, 29.8, 26.7, 26.6, 23.7, 22.9, 22.7, 22.7, 16.3.

^{31}P NMR (202 MHz, CD_3CN) δ_P 148.1, 148.0.

HRMS (ESI/Q-TOF) $[M+H]^+$ calcd. for $C_{52}H_{62}N_6O_8P$ 929.4361, found 929.4310.

Synthesis of 5-(3-(1*H*-indol-3-yl)prop-1-yn-1-yl)-4-amino-1-((2R,4S,5R)-5-((bis(4-methoxyphenyl)(phenyl)methoxy)methyl)-4-hydroxytetrahydrofuran-2-yl)pyrimidin-2(1*H*)-one (15):

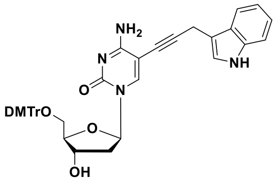

To a solution of compound **11** (1.1 g, 2.9 mmol) in dry pyridine (10 mL), DMAP (35 mg, 0.29 mmol) was added under stirring. To this mixture, a solution of 4,4'-dimethoxytrityl chloride (1.2 g, 3.5 mmol) in 5 mL of anhydrous pyridine was added in four equal portions over the time of 1 h. The reaction mixture was stirred at room temperature for 12 h. After completion of the reaction, the solvent was evaporated under reduced pressure. The resulting crude mixture was purified by flash chromatography using (0–2% MeOH in CH_2Cl_2, with 0.2% of Et_3N) as the eluent, yielding the desired compound **15** (1.1 g, 1.6 mmol, 56%) as a white solid.

R_f DCM/Methanol 9:1, v/v) 0.45.

^{1}H NMR (500 MHz, DMSO-*d*$_6$) δ_H 10.87 (s, 1H), 7.86 (s, 1H), 7.75 (s, 1H), 7.57 (d, *J* = 7.9 Hz, 1H), 7.38 (dd, *J* = 11.2, 8.2 Hz, 3H), 7.27 (t, *J* = 6.7 Hz, 6H), 7.19 – 7.12 (m, 2H), 7.08 (t, *J* = 7.6 Hz, 1H), 6.96 (t, *J* = 7.4 Hz, 1H), 6.83 (dd, *J* = 8.6, 6.0 Hz, 4H), 6.77 (s, 1H), 6.12 (t, *J* = 6.6 Hz, 1H), 5.29 (d, *J* = 4.4 Hz, 1H), 4.24 (bs, 1H), 3.93 (dt, *J* = 2.0, 6.1 Hz, 1H), 3.75 (s, 2H), 3.68 (s, 6H), 3.23 (dd, *J* = 10.4, 5.2 Hz, 1H), 3.08 (dd, *J* = 10.4, 2.4 Hz, 1H), 2.22 (ddd, *J* = 12.8, 5.8, 3.3 Hz, 1H), 2.11 (dt, *J* = 11.4, 6.7 Hz, 1H).

^{13}C NMR (126 MHz, DMSO-*d*$_6$) δ_C 165.0, 158.5, 158.5, 153.9, 145.2, 143.4, 136.8, 136.1, 135.7, 130.3, 130.1, 128.3, 128.0, 127.1, 126.9, 123.4, 121.6, 118.9, 118.7, 113.7, 113.6, 111.9, 109.7, 94.7, 91.1, 86.3, 86.2, 85.9, 72.2, 71.1, 64.2, 55.4, 41.2, 16.3.

HRMS (ESI/Q-TOF) $[M+H]^+$ calcd. for $C_{41}H_{39}N_4O_6$ 682.2791, found 682.2862.

Synthesis of N-(5-(3-(1*H*-indol-3-yl)prop-1-yn-1-yl)-1-((2R,4S,5R)-5-((bis(4-methoxyphenyl)(phenyl)methoxy)methyl)-4-hydroxytetrahydrofuran-2-yl)-2-oxo-1,2-dihydropyrimidin-4-yl)acetamide (16):

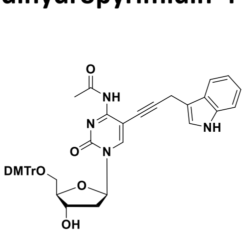

To a solution of compound **15** (1.0 g, 1.5 mmol) in dry DMF (15 mL), acetic anhydride (0.15 mL, 1.6 mmol) was added dropwise over 15 minutes at 0 °C under an argon atmosphere. The reaction mixture was then stirred at room temperature for 10 h. Progress of the reaction was monitored by TLC. After completion of the reaction, the reaction mixture was concentrated under reduced pressure. The

crude product was purified by flash chromatography (0–2% MeOH in CH_2Cl_2, with 0.2% of Et_3N) as the eluent, yielding the desired compound **16** (0.6 g, 0.83 mmol, 57%) as a pale-yellow solid.

R_f (DCM/Methanol 9:1, v/v) 0.55.

^{1}H NMR (500 MHz, DMSO-*d$_6$*) δ_H 10.91 (s, 1H), 9.18 (s, 1H), 8.22 (s, 1H), 7.55 (d, *J* = 7.8 Hz, 1H), 7.40 (d, *J* = 7.7 Hz, 2H), 7.36 (d, *J* = 8.1 Hz, 1H), 7.29 (t, *J* = 6.9 Hz, 6H), 7.22 – 7.16 (m, 2H), 7.09 (t, *J* = 7.5 Hz, 1H), 6.97 (t, *J* = 7.4 Hz, 1H), 6.85 (dd, *J* = 8.4, 5.8 Hz, 4H), 6.06 (t, *J* = 6.2 Hz, 1H), 5.36 (d, *J* = 4.4 Hz, 1H), 4.28 (bs, 1H), 4.03 (bs, 1H), 3.72 (s, 2H), 3.68 (s, 6H), 3.24 (dd, *J* = 10.5, 5.3 Hz, 1H), 3.14 (dd, *J* = 8.5, 4.1 Hz, 1H), 2.43 – 2.34 (m, 1H), 2.28 (s, 3H), 2.25 – 2.15 (m, 1H).

^{13}C NMR (126 MHz, DMSO-*d$_6$*) δ_C 170.5, 161.3, 158.5, 158.5, 152.9, 146.3, 145.2, 136.8, 136.0, 135.7, 130.2, 130.1, 128.4, 128.0, 127.1, 126.8, 123.4, 121.7, 119.0, 118.6, 113.7, 113.7, 112.0, 109.3, 96.1, 94.0, 87.4, 86.8, 86.4, 71.5, 70.7, 63.9, 55.4, 41.4, 25.6, 16.2.

HRMS (ESI/Q-TOF) $[M+H]^+$ calcd. for $C_{43}H_{41}N_4O_7$ 725.2975, found 725.2970.

Synthesis of (2R,3S,5R)-5-(5-(3-(1*H*-indol-3-yl)prop-1-yn-1-yl)-4-acetamido-2-oxopyrimidin-1(2*H*)-yl)-2-((bis(4-methoxyphenyl)(phenyl)methoxy)methyl)tetrahydrofuran-3-yl (2-cyanoethyl) diisopropylphosphoramidite (2b):

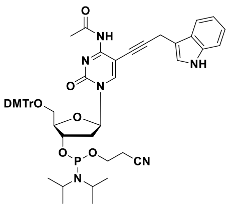

To a solution of compound **16** (0.3 g, 0.41 mmol) in anhydrous CH_2Cl_2, *N,N*-diisopropylethylamine (0.36 mL, 2.1 mmol) was added under stirring. The reaction mixture was cooled to 0 °C, and 2-cyanoethyl-*N,N*-diisopropylchlorophosphoramidite (0.14 mL, 0.66 mmol) was added under an argon atmosphere. The mixture was allowed to warm to room temperature and stirred for 1.5 h. The reaction mixture was then diluted with anhydrous CH_2Cl_2 (20 mL) and washed successively with 5% $NaHCO_3$ (20 mL) and brine solution (20 mL). The organic phase was dried over anhydrous Na_2SO_4 and concentrated under reduced pressure. The crude product was purified by column chromatography using (10-60% EtOAc in hexane containing 0.5% Et_3N) as the eluent. The desired compound **2b** was obtained as a 1:1.1 mixture of two diastereomers in the form of a white foam (0.24 g, 0.26 mmol, 64% yield).

R_f (EtOAc/Hexane 7:3, v/v) 0.5, 0.55.

^{1}H NMR (500 MHz, CD_3CN) δ_H 9.16 (s, 1H), 8.31 (s, 1H), 8.12 (bs, 1H), 7.60 (d, *J* = 7.9 Hz, 1H), 7.51 (d, *J* = 7.4 Hz, 2H), 7.45 (d, *J* = 8.2 Hz, 1H), 7.42–7.38 (m, 4H), 7.33 (t, *J* = 7.6 Hz, 2H), 7.25 (t, *J* = 7.3 Hz, 1H), 7.19 (t, *J* = 7.6 Hz, 1H), 7.11–7.05 (m, 2H), 6.86 (dd, *J* = 8.8, 5.3 Hz, 4H), 6.10 (t, *J* = 6.1 Hz, 1H), 4.71 (td, *J* = 10.3, 4.3 Hz, 1H), 4.26 (d, *J* = 3.1 Hz, 1H), 3.75 (t, *J* = 4.9 Hz, 8H), 3.71–3.61 (m, 3H), 3.34-3.29 (m, 3H), 2.70 – 2.64 (m, 1H), 2.49 – 2.42 (m, 1H), 2.40 (s, 3H), 1.39-1.29 (s, 2H), 1.22 (t, *J* = 6.7 Hz, 12H).

^{13}C NMR (126 MHz, CD_3CN) δ_C 160.3, 158.8, 158.7, 145.3, 144.9, 136.6, 135.9, 135.5, 130.2, 130.1, 128.0, 128.0, 126.9, 126.6, 122.7, 121.8, 119.2, 118.4, 118.2, 113.2, 113.2, 111.5, 109.7, 96.8, 87.5, 86.7, 86.0, 85.9, 72.5, 72.3, 70.5, 62.5, 60.0, 58.6, 58.5, 57.2, 54.9, 43.1, 43.0, 40.5, 25.2, 23.9, 23.9, 23.9, 23.9, 21.0, 20.2, 20.1, 20.0, 15.7, 13.6.

^{31}P NMR (202 MHz, CD_3CN) δ_P 148.1. 148.1.

HRMS (ESI/Q-TOF) $[M+H]^+$ calcd. for $C_{52}H_{57}N_6O_8P$ 925.4048, found 925.4040.

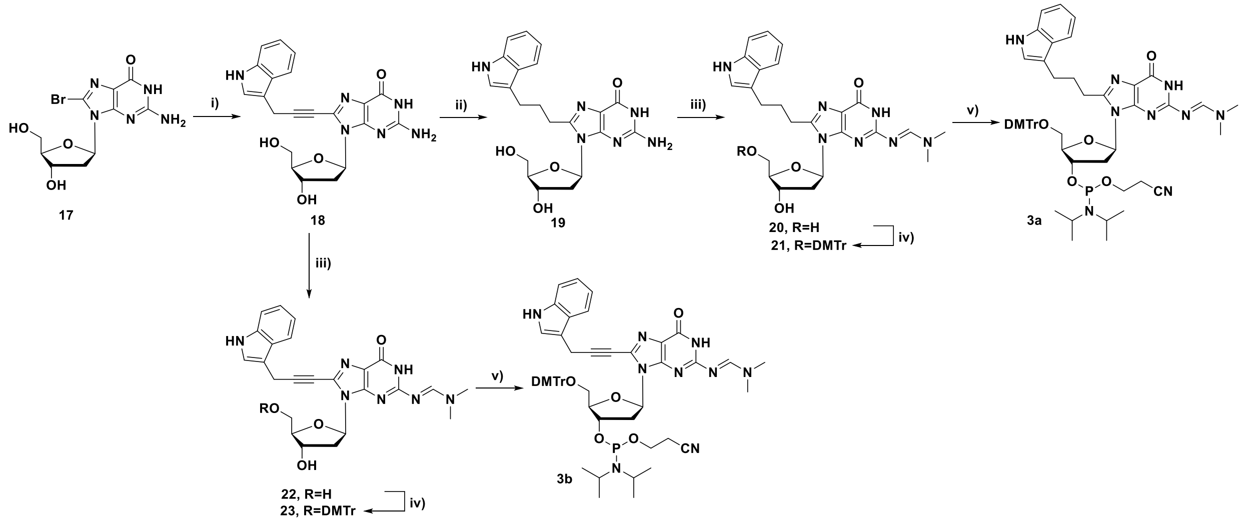

Scheme S3: (i) $Pd(PPh_3)_4$, CuI, Et_3N, 1*H*-Indole,3-(2-propynyl), DMF, 60 °C 3 h, 58% (18) (ii) H_2, 10% Pd/C, MeOH, 50 °C, 12 h, 76% (19) (iii) DMF-DMA, DMF, rt, 1 h, 73% (20), 66% (22) (iv) DMTrCl, DMAP, pyridine, rt, 12 h, 53% (21), 58% (23) (v) 2-cyanoethyl-*N*,*N*-diisopropylchlorophosphoramidite, DIPEA, DCM, 0 °C to rt, 1.5 h, 55% (3a), 69% (3b).

Synthesis of 8-(3-(1H-indol-3-yl)prop-1-yn-1-yl)-2-amino-9-((2R,4S,5R)-4-hydroxy-5-hydroxymethyl)tetrahydrofuran-2-yl)-1,9-dihydro-6H-purin-6-one (18):

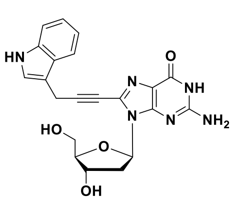

To a solution of 8-Bromo-2'-deoxyguanosine (**17**) (2.0 g, 5.78 mmol) and 3-(prop-2-yn-1-yl)-1*H*-indole[1] (1.17 g, 7.51 mmol) were added to a mixture of $Pd(PPh_3)4$ (0.67 g, 0.58 mmol) and CuI (0.33 g, 1.73 mmol) in anhydrous DMF (40 mL) under a Ar atmosphere in a flask equipped with a gas inlet tube and a magnetic stirrer. Then pump/purge cycles were applied with the addition of Ar gas Et_3N (4.03 mL, 28.9 mmol) was added and then the mixture was heated at 60 °C for 3 h. The progress of the reaction was followed using TLC. After completion of the reaction, evaporated under reduced pressure and the crude product was purified by flash chromatography (0–15% MeOH in CH_2Cl_2). The desired compound **18** (1.4 g, 3.3 mmol, 58%) was obtained as a brown solid.

R_f (DCM/Methanol 8:2, v/v) 0.5.

^{1}H NMR (500 MHz, DMSO-*d_6*) δ_H 10.99 (s, 1H), 10.76 (s, 1H), 7.63 (d, *J* = 7.8 Hz, 1H), 7.39 (d, *J* = 8.1 Hz, 1H), 7.31 (s, 1H), 7.12 (t, *J* = 7.5 Hz, 1H), 7.04 (t, *J* = 7.3 Hz, 1H), 6.49 (s, 2H), 6.29 (t, *J* = 7.3 Hz, 1H), 5.22 (d, *J* = 4.1 Hz, 1H), 4.87 (t, *J* = 5.8 Hz, 1H), 4.35 (app q, J_{app} ~ 2.6 Hz, 1H), 4.03 (s, 2H), 3.80 (dt, *J* = 6.8, 2.9 Hz, 1H), 3.64 – 3.56 (m, 1H), 3.48 (dd, *J* = 11.8, 6.0 Hz, 1H), 3.12 – 3.02 (m, 1H), 2.11 – 2.03 (m, 1H).

^{13}C NMR (126 MHz, DMSO-*d_6*) δ_C 156.5, 154.2, 151.0, 136.8, 130.3, 126.8, 123.5, 121.8, 119.1, 118.8, 117.4, 112.0, 108.5, 94.3, 88.2, 84.3, 71.6, 71.5, 62.6, 37.4, 16.0.

HRMS (ESI/Q-TOF) $[M+H]^+$ calcd. for $C_{21}H_{21}N_6O_4$ 421.1624, found 421.1617.

Synthesis of 8-(3-(1*H*-indol-3-yl)propyl)-2-amino-9-((2R,4S,5R)-4-hydroxy-5-(hydroxymethyl)tetrahydrofuran-2-yl)-1,9-dihydro-6*H*-purin-6-one (19):

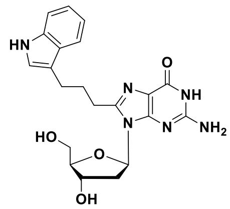

To a solution of compound **18** (1.3 g, 3.1 mmol) in methanol (60 mL), 10% Pd/C (0.33 g, 0.31 mmol) was added under stirring. The reaction mixture was then stirred under a hydrogen atmosphere at 50 °C for 12 h. The progress of the reaction was followed using TLC. After completion of the reaction, the reaction mixture was filtered through celite to remove the catalyst. The filtrate was evaporated under reduced pressure to yield the pure compound **19** (1.0 g, 2.4 mmol, 76 %) as a pale-yellow solid.

R$_f$ (DCM:Methanol 8:2, v/v) 0.5.

^{1}H NMR (500 MHz, DMSO-*d$_6$*) δ_H 10.77 (s, 1H), 10.61 (s, 1H), 7.53 (d, *J* = 7.9 Hz, 1H), 7.34 (d, *J* = 8.0 Hz, 1H), 7.14 (s, 1H), 7.06 (t, *J* = 7.5 Hz, 1H), 6.96 (t, *J* = 7.4 Hz, 1H), 6.32 (s, 2H), 6.17 – 6.10 (m, 1H), 5.22 (d, *J* = 1.4 Hz, 1H), 4.99 (s, 1H), 4.34 (dt, *J* = 6.8, 2.0 Hz, 1H), 3.82 – 3.76 (m, 1H), 3.60 (dd, *J* = 11.4, 4.3 Hz, 1H), 3.49 (dd, *J* = 10.6, 4.9 Hz, 1H), 2.93 – 2.81 (m, 2H), 2.81 – 2.74 (m, 3H), 2.11 – 2.00 (m, 3H).

^{13}C NMR (126 MHz, DMSO-*d$_6$*) δ_C 156.8, 153.2, 152.0, 148.6, 136.8, 127.6, 122.7, 121.3, 118.8, 118.6, 116.0, 114.6, 111.8, 88.0, 83.7, 71.5, 62.5, 49.1, 28.2, 27.7, 24.7.

HRMS (ESI/Q-TOF) [M+H]$^+$ calcd. for $C_{21}H_{25}N_6O_4$ 425.1932, found 425. 1932.

Synthesis of (E)-N'-(8-(3-(1*H*-indol-3-yl)propyl)-9-((2R,4S,5R)-4-hydroxy-5-(hydroxymethyl)tetrahydrofuran-2-yl)-6-oxo-6,9-dihydro-1*H*-purin-2-yl)-N,N-dimethylformimidamide (20):

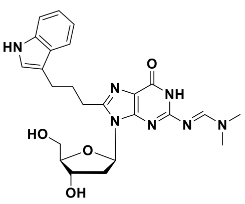

To a solution of compound **19** (0.80 g, 1.88 mmol) in anhydrous DMF (10 mL) under argon atmosphere, dimethylformamide dimethylacetal (5.0 mL, 37.7 mmol) was added under stirring. The reaction mixture was continued to stir at room temperature for 1 h. The progress of the reaction was followed using TLC. After the reaction was complete, the solvent was removed under reduced pressure. The crude product was purified by flash chromatography using (0–10% MeOH in DCM) as eluent. The pure product **20** (0.66 g, 1.4 mmol, 73%) was obtained as a white solid.

R$_f$ (DCM/Methanol 9:1, v/v) 0.4.

^{1}H NMR (500 MHz, DMSO-*d$_6$*) δ_H 11.30 (s, 1H), 10.77 (s, 1H), 8.46 (s, 1H), 7.53 (d, *J* = 7.8 Hz, 1H), 7.34 (d, *J* = 8.1 Hz, 1H), 7.15 (d, *J* = 2.0 Hz, 1H), 7.06 (dd, *J* = 11.1, 4.0 Hz, 1H), 6.97 (t, *J* = 7.4 Hz, 1H), 6.18 (t, *J* = 7.3 Hz, 1H), 5.29 (d, *J* = 4.4 Hz, 1H), 4.90 (dd, *J* = 6.7, 5.0 Hz, 1H), 4.42 (dt, *J* = 7.2, 3.6 Hz, 1H), 3.80 (dt, *J* = 7.9, 4.4 Hz, 1H), 3.63 (dd, *J* = 11.5, 4.6 Hz, 1H), 3.51 (dd, *J* = 11.7, 5.0 Hz, 1H), 3.13 (s, 3H), 3.02 (s, 3H), 2.97 (dt, *J* = 12.2, 7.0 Hz, 1H), 2.82 (m, 4H), 2.09 (m, 3H).

^{13}C NMR (126 MHz, DMSO-*d$_6$*) δ_C 158.4, 157.7, 156.9, 150.7, 149.7, 136.8, 127.8, 122.8, 121.3, 119.2, 118.8, 118.6, 114.5, 111.8, 87.9, 83.8, 71.3, 62.4, 41.2, 38.4, 35.0, 28.2, 27.7, 24.7.

HRMS (ESI/Q-TOF) [M+H]$^+$ calcd. for $C_{24}H_{30}N_7O_4$ 480.2353, found 480.2348.

Synthesis of (E)-N'-(8-(3-(1*H*-indol-3-yl)propyl)-9-((2R,4S,5R)-5-((bis(4-methoxyphenyl)(phenyl)methoxy)methyl)-4-hydroxytetrahydrofuran-2-yl)-6-oxo-6,9-dihydro-1*H*-purin-2-yl)-N,N-dimethylformimidamide (21):

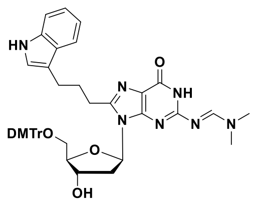

To a solution of compound **20** (0.5 g, 1.04 mmol) in dry pyridine (10 mL), DMAP (13 mg, 0.10 mmol) was added. To this mixture, a solution of 4,4′-dimethoxytrityl chloride (0.42 g, 1.25 mmol) in 3 mL of anhydrous pyridine was added in four equal portions over the time of 1 h. The reaction mixture was stirred at room temperature for 12 h. After completion of the reaction, the solvent was evaporated under reduced pressure. The resulting crude mixture was purified by flash chromatography (0–5% MeOH in CH_2Cl_2, with 0.2% of Et_3N) as the eluent, yielding the desired compound **21** (0.43 g, 0.55 mmol, 53%) as a pale-yellow solid.

$\mathbf{R_f}$ (DCM/Methanol 9:1, v/v) 0.45.

^{1}H NMR (500 MHz, DMSO-*d*$_6$) δ_H 11.28 (s, 1H), 10.76 (s, 1H), 8.32 (s, 1H), 7.50 (d, *J* = 7.9 Hz, 1H), 7.33 (d, *J* = 8.1 Hz, 1H), 7.29 (d, *J* = 7.0 Hz, 2H), 7.17 (m 7H), 7.10 (d, *J* = 1.7 Hz, 1H), 7.05 (t, *J* = 7.6 Hz, 1H), 6.94 (t, *J* = 7.3 Hz, 1H), 6.77 (d, *J* = 8.8 Hz, 2H), 6.73 (d, *J* = 8.8 Hz, 2H), 6.28 – 6.22 (m, 1H), 5.32 (d, *J* = 4.9 Hz, 1H), 4.55 (dt, *J* = 11.7, 5.7 Hz, 1H), 3.87 (dt, *J* = 6.4, 3.7 Hz, 1H), 3.70 (s, 3H), 3.69 (s, 3H), 3.19 (dd, *J* = 11.9, 6.0 Hz, 1H), 3.12 (dd, *J* = 10.1, 2.7 Hz, 1H), 3.07 (dd, *J* = 12.7, 6.7 Hz, 1H), 3.01 (s, 6H), 2.88 (t, *J* = 7.4 Hz, 2H), 2.77 (t, *J* = 7.5 Hz, 2H), 2.23 – 2.07 (m, 3H).

^{13}C NMR (126 MHz, DMSO-*d*$_6$) δ_C 158.4, 158.3, 157.9, 157.7, 156.5, 150.5, 149.9, 145.4, 136.8, 136.2, 136.0, 130.1, 129.9, 128.1, 127.6, 127.0, 122.6, 121.3, 119.2, 118.8, 118.6, 114.6, 113.4, 113.4, 111.8, 85.7, 83.2, 70.9, 64.3, 55.4, 55.4, 41.2, 38.3, 35.0, 28.1, 27.7, 24.8.

HRMS (ESI/Q-TOF) $[M+H]^+$ calcd. for $C_{45}H_{48}N_7O_6$ 782.3661, found 782.3659.

Synthesis of (2R,3S,5R)-5-(8-(3-(1*H*-indol-3-yl)propyl)-2-(((E)-(dimethylamino)methylene)amino)-6-oxo-1,6-dihydro-9*H*-purin-9-yl)-2-((bis(4-methoxyphenyl)(phenyl)methoxy)methyl)tetrahydrofuran-3-yl (2-cyanoethyl) diisopropylphosphoramidite (3a):

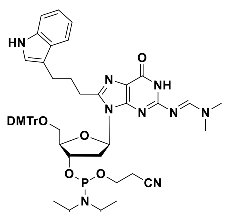

To a solution of compound **21** (0.35 g, 0.45 mmol) in anhydrous CH_2Cl_2, *N,N*-diisopropylethylamine (0.39 mL, 2.24 mmol) was added under stirring. The reaction mixture was cooled to 0 °C, and 2-cyanoethyl-*N,N*-diisopropylchlorophosphoramidite (0.15 mL, 0.72 mmol) was added under an argon atmosphere. The mixture was allowed to warm to room temperature and stirred for 1.5 h. The reaction mixture was then diluted with anhydrous CH_2Cl_2 (20 mL) and washed successively with 5% $NaHCO_3$ (20 mL) and brine solution (20 mL). The organic phase was dried over anhydrous Na_2SO_4 and concentrated under reduced pressure. The resulting crude mixture was purified by flash chromatography (10–60% acetone in hexane containing 0.5% Et_3N) as the eluent. The desired compound **3a** was obtained as a 1:1.1 mixture of two diastereomers in the form of a pale-yellow foam (0.24 g, 0.22 mmol, 55% yield).

$\mathbf{R_f}$ (Acetone/Hexane 1:1, v/v) 0.45, 0.5.

^{1}H NMR (500 MHz, CD_3CN) δ_H 9.52 (s, 1H), 9.13 (s, 1H), 8.41 (s, 1H), 7.60 (dd, *J* = 7.7, 4.6 Hz, 1H), 7.42 (d, *J* = 8.1 Hz, 1H), 7.36 (ddd, *J* = 12.0, 8.1, 1.4 Hz, 2H), 7.28 – 7.17 (m, 7H), 7.18 – 7.13 (m, 1H), 7.11 (d, *J* = 2.0 Hz, 1H), 7.07 – 7.01 (m, 1H), 6.81 – 6.71 (m, 4H), 6.21 (dt, *J* = 8.2, 4.2 Hz, 1H), 5.09 – 4.94 (m, 1H), 4.13 – 4.03 (m, 1H), 3.75 (s, 6H), 3.65 – 3.55 (m, 3H), 3.34 – 3.22 (m, 3H), 3.06 (s, 6H), 2.97 – 2.86 (m, 4H), 2.65 –

2.60 (m, 2H), 2.48 (t, *J* = 6.0 Hz, 1H), 2.37 (ddd, *J* = 11.2, 3.8, 7.0 Hz, 1H), 2.31 – 2.25 (m, 2H), 1.22 – 1.13 (m, 12H).

^{13}C NMR (126 MHz, CD_3CN) δ_C 158.6, 158.5, 158.5, 157.6, 157.4, 156.1, 150.6, 150.3, 145.2, 145.1, 136.6, 136.1, 136.0, 135.8, 135.8, 129.9, 129.8, 129.7, 127.9, 127.9, 127.7, 127.7, 127.5, 126.8, 126.7, 122.2, 121.4, 119.3, 118.7, 118.6, 114.9, 112.9, 111.3, 85.8, 85.8, 84.6, 84.5, 83.4, 83.3, 74.2, 74.0, 73.4, 73.2, 63.8, 63.5, 58.7, 58.5, 58.4, 58.2, 54.9, 54.9, 54.9, 43.2, 43.2, 43.1, 43.1, 40.7, 37.1, 36.9, 34.3, 29.9, 27.8, 27.8, 27.4, 24.3, 23.9, 23.9, 23.9, 23.9, 23.9, 23.8, 23.8, 20.1, 20.0, 19.9.

^{31}P NMR (202 MHz, CD_3CN) δ_P 149.8, 149.6.

HRMS (ESI/Q-TOF) $[M+H]^+$ calcd. for $C_{54}H_{65}N_9O_7P$ 982.4739, found 982.4736.

Synthesis of (E)-N'-(8-(3-(1*H*-indol-3-yl)prop-1-yn-1-yl)-9-((2R,4S,5R)-4-hydroxy-5-(hydroxymethyl)tetrahydrofuran-2-yl)-6-oxo-6,9-dihydro-1*H*-purin-2-yl)-N,N-dimethylformimidamide (22):

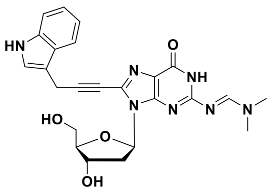

To a solution of compound **18** (0.7 g, 1.66 mmol) in anhydrous DMF (8 mL) under argon atmosphere, dimethylformamide dimethylacetal (4.4 mL, 33.3 mmol) was added under stirring. The reaction mixture was stirred at room temperature for 1 h. The progress of the reaction was followed using TLC. After the reaction was complete, the solvent was removed under reduced pressure. The crude product was purified by flash chromatography using (0–10% MeOH in DCM as the eluent. The pure compound **22** (0.52 g, 1.1 mmol, 66%) was obtained as a pale-yellow solid.

R_f (DCM/Methanol, 9:1, v/v) 0.45.

^{1}H NMR (500 MHz, DMSO-*d$_6$*) δ_H 11.49 (s, 1H), 10.99 (s, 1H), 8.50 (s, 1H), 7.64 (d, *J* = 7.8 Hz, 1H), 7.39 (d, *J* = 8.1 Hz, 1H), 7.32 (s, 1H), 7.12 (t, *J* = 7.5 Hz, 1H), 7.05 (t, *J* = 7.4 Hz, 1H), 6.38 (t, *J* = 7.2 Hz, 1H), 5.31 (d, *J* = 4.1 Hz, 1H), 4.84 (t, *J* = 5.9 Hz, 1H), 4.43 (dt, *J* = 6.6, 3.5 Hz, 1H), 4.05 (s, 2H), 3.82 (dt, *J* = 8.2, 4.8 Hz, 1H), 3.68 – 3.59 (m, 1H), 3.57 – 3.47 (m, 1H), 3.15 (s, 3H), 3.10 – 3.06 (m, 1H), 3.04 (s, 3H), 2.13 (ddd, *J* = 12.8, 6.5, 3.0 Hz, 1H).

^{13}C NMR (126 MHz, DMSO-*d$_6$*) δ_C 158.7, 158.0, 157.4, 149.7, 136.8, 131.5, 126.8, 123.6, 121.8, 120.5, 119.12, 118.8, 112.1, 108.4, 95.0, 88.2, 84.6, 71.3, 71.2, 62.5, 41.3, 38.0, 35.1, 16.0.

HRMS (ESI/Q-TOF) $[M+H]^+$ calcd. for $C_{24}H_{26}N_7O_4$ 476.2046, found 476.2037.

Synthesis of (E)-N'-(8-(3-(1*H*-indol-3-yl)prop-1-yn-1-yl)-9-((2R,4S,5R)-5-((bis(4-methoxyphenyl)(phenyl)methoxy)methyl)-4-hydroxytetrahydrofuran-2-yl)-6-oxo-6,9-dihydro-1*H*-purin-2-yl)-N,N-dimethylformimidamide (23):

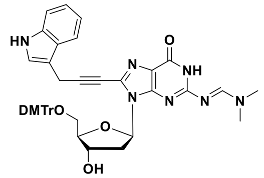

To a solution of compound **22** (0.4 g, 0.84 mmol) in dry pyridine (8 mL), DMAP (10 mg, 0.1 mmol) was added under stirring. To this mixture, a solution of 4,4'-dimethoxytrityl chloride (0.34 g, 1.01 mmol) in 4 mL of anhydrous pyridine was added in four equal portions over the time of 1 h. The reaction mixture was stirred at room temperature for 12 h. After completion of the reaction, the solvent was evaporated under reduced pressure. The resulting crude residue was purified by flash chromatography

(0–5% MeOH in CH_2Cl_2, with 0.2% of Et_3N) as a eluent, yielding the desired compound **23** (0.38 g, 0.49 mmol, 58%) as a pale-yellow form.

R$_f$ (DCM/Methanol, 9:1, v/v) 0.55.

^{1}H NMR (500 MHz, DMSO-*d$_6$*) δ_H 11.46 (s, 1H), 10.98 (s, 1H), 8.33 (s, 1H), 7.61 (d, *J* = 7.9 Hz, 1H), 7.40 (d, *J* = 8.1 Hz, 1H), 7.30 (d, *J* = 6.9 Hz, 2H), 7.25 (d, *J* = 1.7 Hz, 1H), 7.20 (d, *J* = 6.5 Hz, 1H), 7.18 – 7.14 (m, 6H), 7.12 (d, *J* = 7.3 Hz, 1H), 7.04 (t, *J* = 7.2 Hz, 1H), 6.74 (dd, *J* = 20.2, 8.9 Hz, 4H), 6.42 (dd, *J* = 7.4, 5.7 Hz, 1H), 5.36 (d, *J* = 4.8 Hz, 1H), 4.48 (dt, *J* = 10.5, 5.1 Hz, 1H), 3.94-3.92 (m, 3H), 3.69 (s, 6H), 3.25 (dd, *J* = 9.7, 7.4 Hz, 1H), 3.18 – 3.07 (m, 2H), 3.03 (s, 6H), 2.27 – 2.16 (m, 1H).

^{13}C NMR (126 MHz, DMSO-*d$_6$*) δ_C 158.4, 158.4, 158.2, 157.7, 157.5, 149.6, 145.4, 136.9, 136.1, 131.5, 130.1, 130.0, 128.1, 127.0, 126.8, 123.5, 121.8, 120.4, 119.1, 118.8, 113.5, 113.4, 112.1, 108.5, 94.7, 86.3, 85.7, 84.0, 71.7, 71.3, 64.7, 55.4, 55.4, 35.1, 16.0.

HRMS (ESI/Q-TOF) $[M+H]^+$ calcd. for $C_{45}H_{44}N_7O_6$ 778.3353, found 778.3357.

Synthesis of (2R,3S,5R)-5-(8-(3-(1*H*-indol-3-yl)prop-1-yn-1-yl)-2-(((E)-(dimethylamino)methylene)amino)-6-oxo-1,6-dihydro-9*H*-purin-9-yl)-2-((bis(4-methoxyphenyl)(phenyl)methoxy)methyl)tetrahydrofuran-3-yl (2-cyanoethyl) diisopropylphosphoramidite (3b):

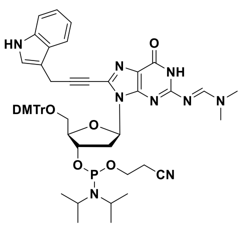

To a solution of compound **23** (0.3 g, 0.39 mmol) in anhydrous CH_2Cl_2, *N,N*-diisopropylethylamine (0.34 mL, 1.93 mmol) was added under stirring. The reaction mixture was cooled to 0 °C, and 2-cyanoethyl-*N,N*-diisopropylchlorophosphoramidite (0.13 mL, 0.62 mmol) was added under an argon atmosphere. The mixture was allowed to warm to room temperature and stirred for 1.5 h. The reaction mixture was then diluted with anhydrous CH_2Cl_2 (20 mL) and washed successively with 5% $NaHCO_3$ (20 mL) and brine solution (20 mL). The organic phase was dried over anhydrous Na_2SO_4 and concentrated under reduced pressure. The crude residue was purified by column chromatography using (10–50% acetone in hexane containing 0.5% Et_3N) as the eluent. The desired compound **3b** was obtained as a 1:1.2 mixture of two diastereomers in the form of a pale-yellow foam (0.26 g, 0.27 mmol, 69% yield).

R$_f$ (Acetone/Hexane 1:1, v/v) 0.5, 0.55.

^{1}H NMR (500 MHz, CD_3CN) δ_H 9.42 (s, 1H), 9.29 (s, 1H), 8.42 (d, *J* = 6.4 Hz, 1H), 7.71 (d, *J* = 7.9 Hz, 1H), 7.49 (d, *J* = 8.2 Hz, 1H), 7.38 (m, 2H), 7.29 (t, *J* = 2.9 Hz, 1H), 7.27 (d, *J* = 1.5 Hz, 1H), 7.26 – 7.19 (m, 7H), 7.17 – 7.12 (m, 1H), 6.82 – 6.72 (m, 4H), 6.52 (td, *J* = 8.1, 4.9 Hz, 1H), 4.98-4.88 (m, 1H), 4.16 – 4.07 (m, 2H), 4.00 (s, 2H), 3.77 (d, *J* = 3.6 Hz, 3H), 3.76 (d, *J* = 2.7 Hz, 3H), 3.72 (ddd, *J* = 10.4, 7.5, 6.0 Hz, 1H), 3.65 – 3.53 (m, 3H), 3.36 – 3.25 (m, 2H), 3.22 (dd, *J* = 10.1, 3.0 Hz, 1H), 3.08 (m, 6H), 2.62 (t, *J* = 6.0 Hz, 1H), 2.50 – 2.42 (m, 1H), 1.19-1.13 (m, 9H), 1.03 (d, *J* = 6.8 Hz, 3H).

^{13}C NMR (126 MHz, CD_3CN) δ_C 158.6, 158.6, 158.5, 158.5, 158.0, 157.9, 157.3, 157.2, 157.1, 149.6, 145.2, 145.2, 136.7, 136.0, 135.9, 135.9, 135.8, 132.2, 130.0, 129.9, 129.8, 129.8, 127.9, 127.9, 127.8, 127.8, 126.8, 126.7, 126.6, 122.9, 122.9, 121.9, 121.9, 120.4, 119.2, 118.6, 118.4, 112.9, 112.9, 111.6, 108.9, 94.4, 94.4, 85.9, 85.8, 84.9, 84.9, 84.2, 84.1, 74.3, 74.1, 71.1, 71.1, 64.1, 63.9, 60.0, 58.7, 58.5, 58.4, 58.3,

54.9, 54.9, 54.9, 43.2, 43.1, 40.9, 40.9, 37.0, 36.8, 34.4, 34.4, 23.9, 23.9, 23.8, 23.8, 20.2, 20.1, 20.0, 19.9, 15.8, 15.7, 13.6.

^{31}P NMR (202 MHz, CD_3CN) δ_P 149.9, 149.8.

HRMS (ESI/Q-TOF) $[M+H]^+$ calcd. for $C_{54}H_{61}N_9O_7P$ 978.4426, found 978.4431.

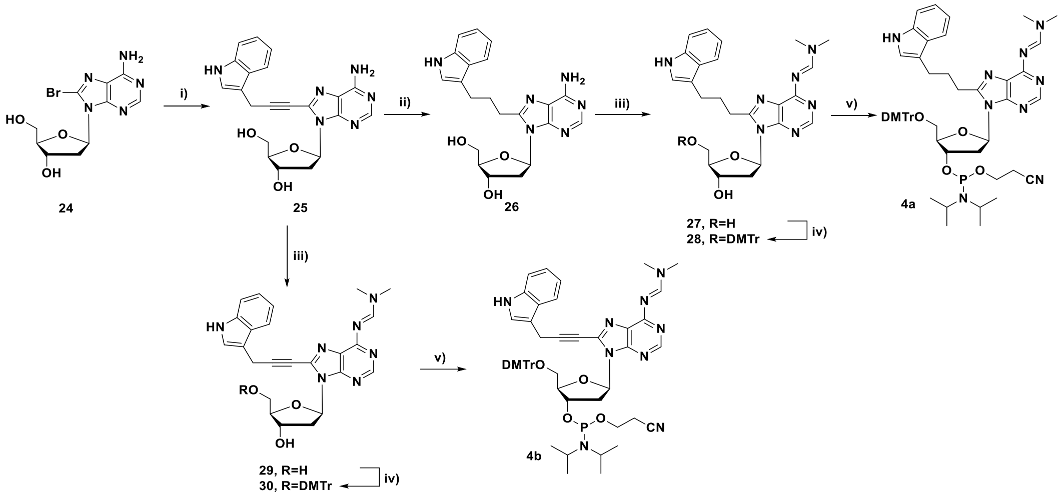

Scheme S4: (i) $Pd(PPh_3)_4$, CuI, Et_3N, 1*H*-Indole,3-(2-propynyl), DMF, 55 °C 2 h, 57% (25) (ii) H_2, 10% Pd/C, MeOH, 50 °C, 12 h, 76% (26) (iii) DMF-DMA, DMF, rt, 1 h, 68% (27), 62% (29) (iv) DMTrCl, DMAP, pyridine, rt, 12 h, 55% (28), 54% (30) (v) 2-cyanoethyl-*N*,*N*-diisopropylchlorophosphoramidite, DIPEA, DCM, 0 °C to rt, 1.5 h, 63% (4a), 61% (4b).

Synthesis of (2R,3S,5R)-5-(8-(3-(1*H*-indol-3-yl)prop-1-yn-1-yl)-6-amino-9*H*-purin-9-yl)-2-(hydroxymethyl)tetrahydrofuran-3-ol (25):

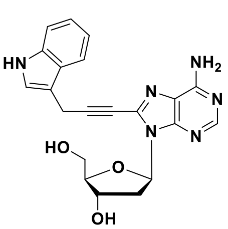

To a solution of 8-Bromo-2'-deoxyadenosine (**24**) (2.0 g, 6.1 mmol) and 3-(prop-2-yn-1-yl)-1*H*-indole[1] (1.2 g, 7.90 mmol) were added to a mixture of $Pd(PPh_3)4$ (0.7 g, 0.61 mmol) and CuI (0.35 g, 1.8 mmol) in anhydrous DMF (30 mL) under a Ar atmosphere in a flask equipped with a gas inlet tube and a magnetic stirrer. Then pump/purge cycles were applied with the addition of Ar gas Et_3N (3.4 mL, 24.0 mmol) was added and then the mixture was heated at 55 °C for 2 h. The progress of the reaction was followed using TLC. After completion of the reaction, evaporated under reduced pressure and the crude product was purified by flash chromatography (0–10% MeOH in CH_2Cl_2). The desired compound **25** (1.4 g, 3.5 mmol, 57%) was obtained as a brown solid.

R_f (DCM/Methanol 9:1, v/v) 0.45.

^{1}H NMR (500 MHz, DMSO-*d_6*) δ_H 11.01 (s, 1H), 8.13 (s, 1H), 7.64 (d, *J* = 7.9 Hz, 1H), 7.50 (s, 2H), 7.40 (d, *J* = 8.1 Hz, 1H), 7.33 (d, *J* = 2.0 Hz, 1H), 7.13 (t, *J* = 7.4 Hz, 1H), 7.05 (t, *J* = 7.4 Hz, 1H), 6.48 (dd, *J* = 7.9, 6.7 Hz, 1H), 5.37 (dd, *J* = 7.9, 4.3 Hz, 1H), 5.33 (d, *J* = 4.0 Hz, 1H), 4.50 – 4.46 (m, 1H), 4.10 (s, 2H), 3.91 (dt, *J* = 6.7, 4.3 Hz, 1H), 3.67 (dd, *J* = 11.8, 4.3 Hz, 1H), 3.53 – 3.47 (m, 1H), 3.15 (m, 1H), 2.18 (ddd, *J* = 12.8, 6.2, 2.2 Hz, 1H).

^{13}C NMR (126 MHz, DMSO-d_6) δ_C 156.37, 153.48, 148.83, 136.81, 133.96, 126.80, 123.64, 121.84, 119.64, 119.16, 118.78, 112.02, 108.26, 96.52, 88.80, 85.66, 71.79, 70.61, 62.69, 38.03, 16.06.

HRMS (ESI/Q-TOF) $[M+H]^+$ calcd. for $C_{21}H_{21}N_6O_3$ 405.1675, found 405.1672.

Synthesis of (2R,3S,5R)-5-(8-(3-(1*H*-indol-3-yl)propyl)-6-amino-9*H*-purin-9-yl)-2-(hydroxymethyl)tetrahydrofuran-3-ol (26):

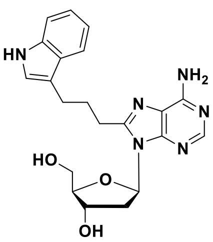

To a solution of compound **25** (1.3 g, 3.2 mmol) in methanol (40 mL), 10% Pd/C (0.34 g, 0.32 mmol) was added under stirring. The reaction mixture was then stirred under a hydrogen atmosphere at 50 °C for 12 h. After completion of the reaction, the reaction mixture was filtered through celite to remove the catalyst. The filtrate was evaporated under reduced pressure to yield the pure compound **26** (1.0 g, 2.4 mmol, 76 %) as a white solid.

R_f (DCM/Methanol 9:1, v/v) 0.4.

^{1}H NMR (500 MHz, DMSO-d_6) δ_H 10.79 (s, 1H), 8.05 (s, 1H), 7.52 (d, *J* = 7.7 Hz, 1H), 7.34 (d, *J* = 8.1 Hz, 1H), 7.17 (m, 3H), 7.06 (d, *J* = 7.9 Hz, 1H), 6.97 (t, *J* = 7.4 Hz, 1H), 6.28 (t, *J* = 7.2 Hz, 1H), 5.58 (dd, *J* = 8.1, 3.6 Hz, 1H), 5.29 (d, *J* = 4.1 Hz, 1H), 4.46 (bs, 1H), 3.90 (bs, 1H), 3.66 (dd, *J* = 11.0, 3.2 Hz, 1H), 3.55 – 3.47 (m, 1H), 3.17 (d, *J* = 5.1 Hz, 1H), 3.13 – 3.05 (m, 1H), 2.97 (t, *J* = 7.5 Hz, 2H), 2.82 (t, *J* = 7.4 Hz, 2H), 2.16-2.11 (m, 3H).

^{13}C NMR (126 MHz, DMSO-d_6) δ_C 155.9, 152.5, 151.7, 150.2, 136.8, 127.6, 122.9, 121.3, 118.8, 118.7, 118.6, 114.3, 111.8, 88.7, 84.9, 71.8, 62.7,38.4, 28.8, 27.7, 24.8.

HRMS (ESI/Q-TOF) $[M+H]^+$ calcd. for $C_{21}H_{25}N_6O_3$ 409.1988, found 409.1990.

Synthesis of (E)-N'-(8-(3-(1H-indol-3-yl)propyl)-9-((2R,4S,5R)-4-hydroxy-5-(hydroxymethyl)tetrahydrofuran-2-yl)-9H-purin-6-yl)-N,N-dimethylformimidamide (27):

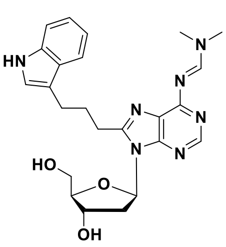

To a solution of compound 26 (0.8 g, 1.96 mmol) in anhydrous DMF (16 mL) under argon atmosphere, dimethylformamide dimethylacetal (3.12 mL, 23.5 mmol) was added. The reaction mixture was stirred at room temperature for 1 h. After the reaction was complete, the solvent was removed under reduced pressure. The crude product was purified by flash chromatography (0–10% MeOH in CH_2Cl_2 with 0.2% of Et_3N). The pure product 28 (0.62 g, 1.34 mmol, 68%) was obtained as a pale-yellow solid.

R_f (DCM/Methanol 9:1, v/v) 0.4.

^{1}H NMR (500 MHz, DMSO-d_6) δ_H 10.79 (s, 1H), 8.86 (s, 1H), 8.33 (s, 1H), 7.57 (d, *J* = 7.9 Hz, 1H), 7.34 (d, *J* = 8.1 Hz, 1H), 7.17 (d, *J* = 2.1 Hz, 1H), 7.06 (t, *J* = 7.5 Hz, 1H), 6.97 (t, *J* = 7.4 Hz, 1H), 6.31 (t, *J* = 7.5 Hz, 1H), 5.35 (dd, *J* = 7.7, 4.2 Hz, 1H), 5.30 (d, *J* = 4.3 Hz, 1H), 4.47 (dt, *J* = 5.0, 2.5 Hz, 1H), 3.88 (app q, J_{app} ~ 5.0 Hz, 1H), 3.67 (dd, *J* = 11.8, 4.2 Hz, 1H), 3.50 (dd, *J* = 12.0, 4.5 Hz, 1H), 3.18 (s, 3H), 3.12 (s, 3H), 3.01 (t, *J* = 7.6 Hz, 2H), 2.84 (t, *J* = 7.4 Hz, 2H), 2.77 (t, *J* = 7.3 Hz, 1H), 2.18-2.13 (m, 3H).

^{13}C NMR (126 MHz, DMSO-d_6) δ_C 158.7, 158.2, 154.5, 152.5, 151.0, 136.8, 127.6, 125.5, 122.8, 121.3, 118.9, 118.6, 114.4, 111.8, 88.5, 84.7, 71.7, 62.6, 41.1, 38.1, 35.1, 28.5, 27.8, 24.8.

HRMS (ESI/Q-TOF) $[M+H]^+$ calcd. for $C_{24}H_{30}N_7O_3$ 464.2410, found 464.2415.

Synthesis of (E)-N'-(8-(3-(1*H*-indol-3-yl)propyl)-9-((2R,4S,5R)-5-((bis(4-methoxyphenyl)(phenyl)methoxy)methyl)-4-hydroxytetrahydrofuran-2-yl)-9*H*-purin-6-yl)-N,N-dimethylformimidamide (28):

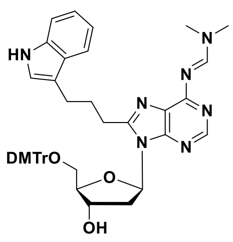

To a solution of compound **27** (0.6 g, 1.29 mmol) in dry pyridine (12 mL), DMAP (16 mg, 0.129 mmol) was added. To this mixture, a solution of 4,4'-dimethoxytrityl chloride (0.52 g, 1.55 mmol) in 4 mL of anhydrous pyridine was added in four equal portions over the time of 1 h. The reaction mixture was stirred at room temperature for 12 h. After completion of the reaction, the solvent was evaporated under reduced pressure. The resulting crude mixture was purified by flash chromatography (0–3% MeOH in CH_2Cl_2 with 0.2% of Et_3N) yielding the desired compound **27** (0.54 g, 0.71 mmol, 55%) as a white solid.

R$_f$ (DCM/Methanol 9:1, v/v) 0.5.

^{1}H NMR (500 MHz, $CDCl_3$) δ_H 8.85 (s, 1H), 8.27 (s, 1H), 7.96 (s, 1H), 7.62 (d, *J* = 7.8 Hz, 1H), 7.41 (d, *J* = 7.3 Hz, 2H), 7.36 (d, *J* = 8.1 Hz, 1H), 7.30 (m, 5H), 7.25 (t, *J* = 7.3 Hz, 2H), 7.20 (t, *J* = 7.1 Hz, 2H), 7.11 (t, *J* = 7.4 Hz, 1H), 7.00 (d, *J* = 1.5 Hz, 1H), 6.78 (dd, *J* = 8.7, 6.6 Hz, 4H), 6.12 (t, *J* = 6.9 Hz, 1H), 4.77 – 4.70 (m, 1H), 3.97 (dd, *J* = 10.2, 5.8 Hz, 1H), 3.78 (s, 6H), 3.46 – 3.36 (m, 3H), 3.25 (s, 3H), 3.18 (s, 3H), 3.01 (t, *J* = 8.0 Hz, 2H), 2.92 (t, *J* = 7.2 Hz, 2H), 2.43 – 2.22 (m, 3H), 2.18 – 2.09 (m, 1H).

^{13}C NMR (126 MHz, $CDCl_3$) δ_C 160.1, 158.4, 158.4, 157.8, 154.8, 152.6, 151.3, 144.8, 136.4, 136.0, 136.1, 130.1, 130.0, 128.2, 127.8, 127.4, 126.8, 125.5, 121.9, 119.2, 119.0, 115.4, 113.1, 111.2, 86.3, 95.4, 83.7, 73.1, 64.0, 55.2, 41.2, 37.1, 35.2, 28.1, 27.9, 24.8.

HRMS (ESI/Q-TOF) $[M+H]^+$ calcd. for $C_{45}H_{48}N_7O_5$ 766.3711, found 766.3688.

Synthesis of (2R,3S,5R)-5-(8-(3-(1*H*-indol-3-yl)propyl)-6-(((E)-(dimethylamino)methylene)amino)-9*H*-purin-9-yl)-2-((bis(4-methoxyphenyl)(phenyl)methoxy)methyl)tetrahydrofuran-3-yl (2-cyanoethyl) diisopropylphosphoramidite (4a):

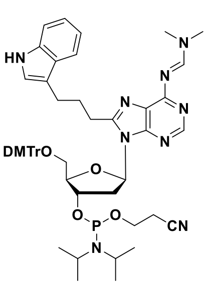

To a solution of compound **28** (0.35 g, 0.46 mmol) in anhydrous CH_2Cl_2, *N,N*-diisopropylethylamine (0.4 mL, 2.28 mmol) was added under stirring. The reaction mixture was cooled to 0 °C, and 2-cyanoethyl-*N,N*-diisopropylchlorophosphoramidite (0.16 mL, 0.73 mmol) was added under an argon atmosphere. The mixture was allowed to warm to room temperature and stirred for 1.5 h. The reaction mixture was then diluted with anhydrous CH_2Cl_2 (20 mL) and washed successively with 5% $NaHCO_3$ (20 mL) and brine solution (20 mL). The organic phase was dried over anhydrous Na_2SO_4 and concentrated under reduced pressure. The crude product was purified by column chromatography using (10–50% acetone in hexane containing 0.5% Et_3N) as the eluent. The desired compound **4a** was obtained as a 1:1.1 mixture of two diastereomers in the form of a white foam (0.28 g, 0.29 mmol, 63% yield).

R$_f$ (Acetone/Hexane 1:1, v/v) 0.45, 0.5.

^{1}H NMR (500 MHz, CD_3CN) δ_H 9.09 (br. s, 1H), 8.87 (s, 1H), 8.26 (dd, *J* = 6.4, 4.4 Hz, 1H), 7.64 (d, *J* = 7.9 Hz, 1H), 7.43 (d, *J* = 8.1 Hz, 1H), 7.34 (d, *J* = 7.7 Hz, 2H), 7.20 (dd, *J* = 8.4, 5.3 Hz, 7H), 7.17 – 7.12 (m, 1H), 7.05

(t, J = 7.5 Hz, 1H), 6.78 – 6.70 (m, 4H), 6.37 – 6.24 (m, 1H), 5.21 – 5.04 (m, 1H), 4.13 – 4.06 (m, 1H), 3.89 – 3.79 (m, 1H), 3.75 (s, 6H), 3.69 (ddd, J = 12.7, 10.3, 4.8 Hz, 2H), 3.65 – 3.54 (m, 3H), 3.32 (ddd, J = 12.3, 10.4, 3.7 Hz, 1H), 3.25 – 3.22 (m, 2H), 3.19 (s, 6H), 3.08 (td, J = 7.6, 1.9 Hz, 1H), 2.96 – 2.89 (m, 2H), 2.68 (t, J = 5.7 Hz, 1H), 2.57 (t, J = 6.0 Hz, 1H), 2.48 – 2.27 (m, 3H), 1.28 – 1.19 (m, 12H).

^{13}C NMR (126 MHz, CD_3CN) δ_C 170.7, 158.6, 158.6, 158.5, 157.7, 154.9, 154.8, 152.5, 151.0, 151.0, 145.2, 145.1, 136.7, 136.2, 136.1, 135.9, 135.8, 130.0, 129.8, 128.0, 127.9, 127.6, 127.5, 126.7, 125.4, 125.4, 122.2, 121.4, 118.7, 118.7, 118.6, 118.4, 114.9, 112.9, 112.8, 111.3, 85.7, 85.7, 84.9, 84.7, 83.7, 83.7, 73.5, 73.4, 72.7, 72.6, 63.6, 63.2, 60.0, 58.8, 58.7, 58.6, 58.5, 54.9, 54.9, 43.1, 43.1, 43.0, 42.9, 40.5, 36.4, 36.1, 34.2, 28.8, 27.9, 27.6, 27.4, 26.6, 24.4, 24.0, 23.9, 23.9, 23.9, 20.2, 20.2, 20.1, 20.1, 20.0, 13.6.

^{31}P NMR (202 MHz, CD_3CN) δ_P 148.4, 148.3.

HRMS (ESI/Q-TOF) $[M+H]^+$ calcd. for $C_{54}H_{65}N_9O_6P$ 966.4790, found 966.4910.

Synthesis of (E)-N'-(8-(3-(1*H*-indol-3-yl)prop-1-yn-1-yl)-9-((2R,4S,5R)-4-hydroxy-5-(hydroxymethyl)tetrahydrofuran-2-yl)-9*H*-purin-6-yl)-N,N-dimethylformimidamide (29):

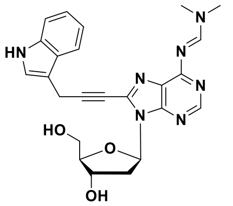

To a solution of compound **25** (0.8 g, 1.98 mmol) in anhydrous DMF (10 mL) under argon atmosphere, dimethylformamide dimethylacetal (5.26 mL, 39.6 mmol) was added. The reaction mixture was stirred at room temperature for 1 h. After the reaction was complete, the solvent was removed under reduced pressure. The crude product was purified by flash chromatography using (0–10% MeOH in DCM) as the eluent. The desired product **29** (0.56 g, 1.22 mmol, 62%) was obtained as a yellow solid.

R_f (DCM/Methanol 9:1, v/v) 0.50.

^{1}H NMR (500 MHz, DMSO-d_6) δ_H 11.03 (s, 1H), 8.88 (s, 1H), 8.41 (s, 1H), 7.66 (d, J = 7.8 Hz, 1H), 7.40 (d, J = 8.1 Hz, 1H), 7.36 (s, 1H), 7.13 (t, J = 7.3 Hz, 1H), 7.06 (t, J = 7.3 Hz, 1H), 6.51 (t, J = 7.2 Hz, 1H), 5.34 (d, J = 2.5 Hz, 1H), 5.20 (dd, J = 6.6, 4.8 Hz, 1H), 4.50 (d, J = 1.9 Hz, 1H), 4.12 (s, 2H), 3.90 (dd, J = 6.7, 4.1 Hz, 1H), 3.67 (dt, J = 10.7, 4.2 Hz, 1H), 3.50 (ddd, J = 11.8, 6.2, 5.3 Hz, 1H), 3.20 (s, 3H), 3.17 (d, J = 4.8 Hz, 1H), 3.12 (s, 3H), 2.20 (ddd, J = 12.9, 6.4, 2.5 Hz, 1H).

^{13}C NMR (126 MHz, DMSO-d_6) δ_C 159.6, 158.4, 152.7, 151.0, 136.9, 135.8, 126.80, 126.1, 123.7, 121.9, 119.2, 118.8, 112.1, 108.4, 97.3, 88.7, 85.5, 71.7, 70.8, 62.7, 41.2, 37.9, 35.1, 16.1.

HRMS (ESI/Q-TOF) $[M+H]^+$ calcd. for $C_{24}H_{26}N_7O_3$ 460.2092, found 460.2088.

Synthesis of (E)-N'-(8-(3-(1*H*-indol-3-yl)prop-1-yn-1-yl)-9-((2R,4S,5R)-5-((bis(4-methoxyphenyl)(phenyl)methoxy)methyl)-4-hydroxytetrahydrofuran-2-yl)-9*H*-purin-6-yl)-N,N-dimethylformimidamide (30):

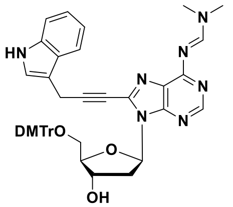

To a solution of compound **29** (0.45 g, 0.98 mmol) in dry pyridine (12 mL), DMAP (12 mg, 0.1 mmol) was added. To this mixture, a solution of 4,4'-dimethoxytrityl chloride (0.4 g, 1.17 mmol) in 8 mL of anhydrous pyridine was added in four equal portions over the course of 1 h. The reaction mixture was stirred at room temperature for 12 h. After completion of the reaction, the solvent was evaporated under reduced pressure. The resulting crude mixture was purified by flash chromatography (0–3%

MeOH in CH_2Cl_2 with 0.2% Et_3N) yielding the desired compound **30** (0.4 g, 0.53 mmol, 54%) as a pale-yellow solid.

R_f (DCM/Methanol 9:1, v/v) 0.6.

^{1}H NMR (500 MHz, DMSO-*d₆*) δ_H 8.85 (s, 1H), 8.27 (s, 1H), 7.96 (s, 1H), 7.62 (d, *J* = 7.8 Hz, 1H), 7.41 (d, *J* = 7.3 Hz, 2H), 7.36 (d, *J* = 8.1 Hz, 1H), 7.30 (s, 1H), 7.29–7.19 (m, 5H), 7.11 (t, *J* = 7.4 Hz, 1H), 7.00 (s, 1H), 6.78 (dd, *J* = 8.7, 6.7 Hz, 4H), 6.12 (t, *J* = 6.9 Hz, 1H), 4.73 (d, *J* = 6.2 Hz, 1H), 3.97 (dd, *J* = 10.2, 5.7 Hz, 1H), 3.78 (s, 6H), 3.46 – 3.36 (m, 3H), 3.25 (s, 3H), 3.18 (s, 3H), 3.01 (dd, *J* = 12.1, 8.1 Hz, 2H), 2.92 (t, *J* = 7.2 Hz, 2H), 2.38 – 2.23 (m, 3H), 2.17 – 2.10 (m, 1H).

^{13}C NMR (126 MHz, DMSO-d_6) δ_C 159.4, 158.4, 158.3, 158.2, 152.8, 151.0, 145.5, 136.9, 136.2, 136.1, 136.1, 130.2, 130.0, 128.1, 128.1, 127.0, 126.8, 126.0, 123.7, 121.9, 119.2, 118.8, 113.5, 113.4, 112.1, 108.2, 96.9, 86.3, 85.7, 84.9, 71.3, 71.2, 55.4, 55.4, 41.2, 37.2, 35.1, 16.1.

HRMS (ESI/Q-TOF) $[M+H]^+$ calcd. for $C_{45}H_{44}N_7O_5$ 762.3404, found 762.3409.

Synthesis of (2R,3S,5R)-5-(8-(3-(1*H*-indol-3-yl)prop-1-yn-1-yl)-6-(((E)-(dimethylamino)methylene)amino)-9*H*-purin-9-yl)-2-((bis(4-methoxyphenyl)(phenyl)methoxy)methyl)tetrahydrofuran-3-yl (2-cyanoethyl) diisopropylphosphoramidite (4b):

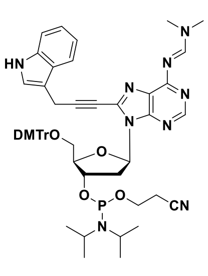

To a solution of compound **30** (0.3 g, 0.39 mmol) in anhydrous CH_2Cl_2, *N,N*-diisopropylethylamine (0.34 mL, 1.97 mmol) was added under stirring. The reaction mixture was cooled to 0 °C, and 2-cyanoethyl-*N,N*-diisopropylchlorophosphoramidite (0.13 mL, 0.63 mmol) was added under an argon atmosphere. The mixture was allowed to warm to room temperature and stirred for 1.5 h. The reaction mixture was then diluted with anhydrous CH_2Cl_2 (20 mL) and washed successively with 5% $NaHCO_3$ (20 mL) and brine solution (20 mL). The organic phase was dried over anhydrous Na_2SO_4 and concentrated under reduced pressure. The crude product was purified by column chromatography using (10–40% acetone in hexane containing 0.5% Et_3N). The desired compound **4b** was obtained as a 1:1.2 mixture of two diastereomers in the form of a pale-yellow foam (0.23 g, 0.24 mmol, 61% yield).

R_f (Acetone/Hexane 1:1, v/v) 0.5, 0.55.

^{1}H NMR (500 MHz, CD_3CN) δ_H 9.29 (br. s, 1H), 8.90 (s, 1H), 8.31 (s, 1H), 7.73 (d, *J* = 7.9 Hz, 1H), 7.49 (d, *J* = 8.1 Hz, 1H), 7.35 (dd, *J* = 10.9, 8.4 Hz, 3H), 7.26 – 7.19 (m, 8H), 7.15 (t, *J* = 7.5 Hz, 1H), 6.80 – 6.71 (m, 4H), 6.64 – 6.57 (m, 1H), 5.19 – 5.00 (m, 1H), 4.24 – 4.14 (m, 1H), 4.07 (d, *J* = 2.7 Hz, 2H), 3.89 – 3.80 (m, 1H), 3.76 (s, 6H), 3.69 (dt, *J* = 12.0, 6.0 Hz, 1H), 3.66-3.49 (m, 3H), 3.41 – 3.28 (m, 1H), 3.28 – 3.22 (m, 1H), 3.21(s, 3H), 3.19 (s, 3H), 2.65 (dd, *J* = 12.7, 6.9 Hz, 1H), 2.54 (s, 1H), 2.54 – 2.42 (m, 1H), 1.27-1.19 (m, 12H).

^{13}C NMR (126 MHz, DMSO-d_6) δ_C 170.7, 159.4, 158.6, 158.5, 157.9, 152.6, 150.9, 145.2, 145.2, 136.7, 136.3, 136.2, 136.1, 136.0, 135.9, 135.9, 130.0, 129.8, 128.0, 127.9, 127.7, 126.7, 126.6, 125.9, 123.0, 121.9, 119.2, 118.5, 118.4, 112.9, 112.9, 111.6, 108.7, 96.1, 96.1, 85.8, 85.8, 85.2, 85.2, 84.9, 84.7, 84.7, 73.6, 73.5, 73.0, 72.9, 70.9, 63.8, 63.6, 60.0, 58.8, 58.7, 58.6, 58.6, 54.9, 54.9, 54.8, 54.3, 43.1, 43.1, 43.0, 42.9, 40.6, 36.5, 36.5, 36.2, 36.2, 34.2, 28.8, 24.0, 23.9, 23.9, 23.9, 20.2, 20.1, 20.1, 20.0, 19.9, 15.8, 13.6.

^{31}P NMR (202 MHz, CD_3CN) δ_P 148.0, 147.8.

HRMS (ESI/Q-TOF) $[M+H]^+$ calcd. for $C_{54}H_{61}N_9O_6P$ 982.4739, found 982.4736.

2. List of base-modified DNA sequences, along with their calculated and measured masses.

Strands	Sequences	Mass calc. [Da]	Mass found [Da]
Z1	●<br>5' - F - TTG*AATT***CCCGGG**TCCAAA - 3'	6512.5	6512.5
Z11	○<br>5' - F - TTG*AATT***CCCGGG**TCCAAA - 3'	6508.5	6508.4
Z4	● ● ● ● ●<br>5' - F - TTG*AAUU***CCCGGG**TCCAAA - 3'	7113.3	7113.5
Z44	○ ○ ○ ○ ○<br>5' - F - TTG*AAUU***CCCGGG**TCCAAA - 3'	7093.2	7093.3
Z5	●<br>5' - F -TTG*AATU***CCCGGG**TCCAAA - 3'	6498.5	6498.4
Z55	○<br>5' - F -TTG*AATU***CCCGGG**TCCAAA - 3'	6494.5	6492.3
Z6	● ●<br>5' - F - TTG*AAUU***CCCGGG**TCCAAA - 3'	6641.7	6642.0
cZ3	3' - Q - TTC*UUAA***GGGCCC**AG - 5'<br>● ● ●	5474.1	5473.7
cZ4	3' - Q - TTC*UUAA***GGGCCC**AG - 5'<br>● ● ● ●	5631.3	5630.8
cZ5	3' - Q - TTC*UUAA***GGGCCC**AG - 5'<br>● ● ● ● ●	5788.5	5793.3

Table S1: ● solid circles: 3-propyl-indole linked nucleotide; ○ open circles: 3-(prop-2-yn-1-yl)-indole linked nucleotide; F: 5'-(6-FAM)-labeled; Q: 3'-(Dabcyl)-labeled. *Italics* EcoR1 restriction site; **bold** XmaI, SmaI restriction site.

3. Thermal studies of ZImera

A Melting points

	WT	Z1	Z5	Z6	Z4
cWT	51°C	44°C	43°C	39°C	33°C
cZ3	44°C	41°C	37°C	35°C	38°C
cZ4	39°C	37°C	36°C	36°C	39°C
cZ5	33°C	31°C	30°C	32°C	43°C

	Z11	Z55	Z44
cWT	42°C	34°C	33°C

B Structural analysis using CD

	WT	Z1	Z5	Z6	Z4
cWT	B	BZ	B	B	Distorted B
cZ3	Distorted B	Distorted B	BZ	Distorted B	B
cZ4	B	Distorted B	BZ	Z	B
cZ5	BZ	Distorted B	Z	BZ	B

	Z11	Z55	Z44
cWT	B	B	B

Figure S1: Melting points and structures of Zimera duplexes. (A) Grid showing the melting points of Zimera duplexes compared to wildtype. Melting point was monitored using FRET assays with a Fluorophore-labeled sense strand (WT; Z1-Z55) and a quencher- labeled antisense strand (cWT; cZ3-cZ5). (B) Grid showing structural variation of Zimera duplexes compared to wildtype. Analysis of the structure was performed by CD spectroscopy using 1: 2.5 ratio of WT/Z1-Z55: cWT/Z3/Z4/Z5 in PBS, 20°C.

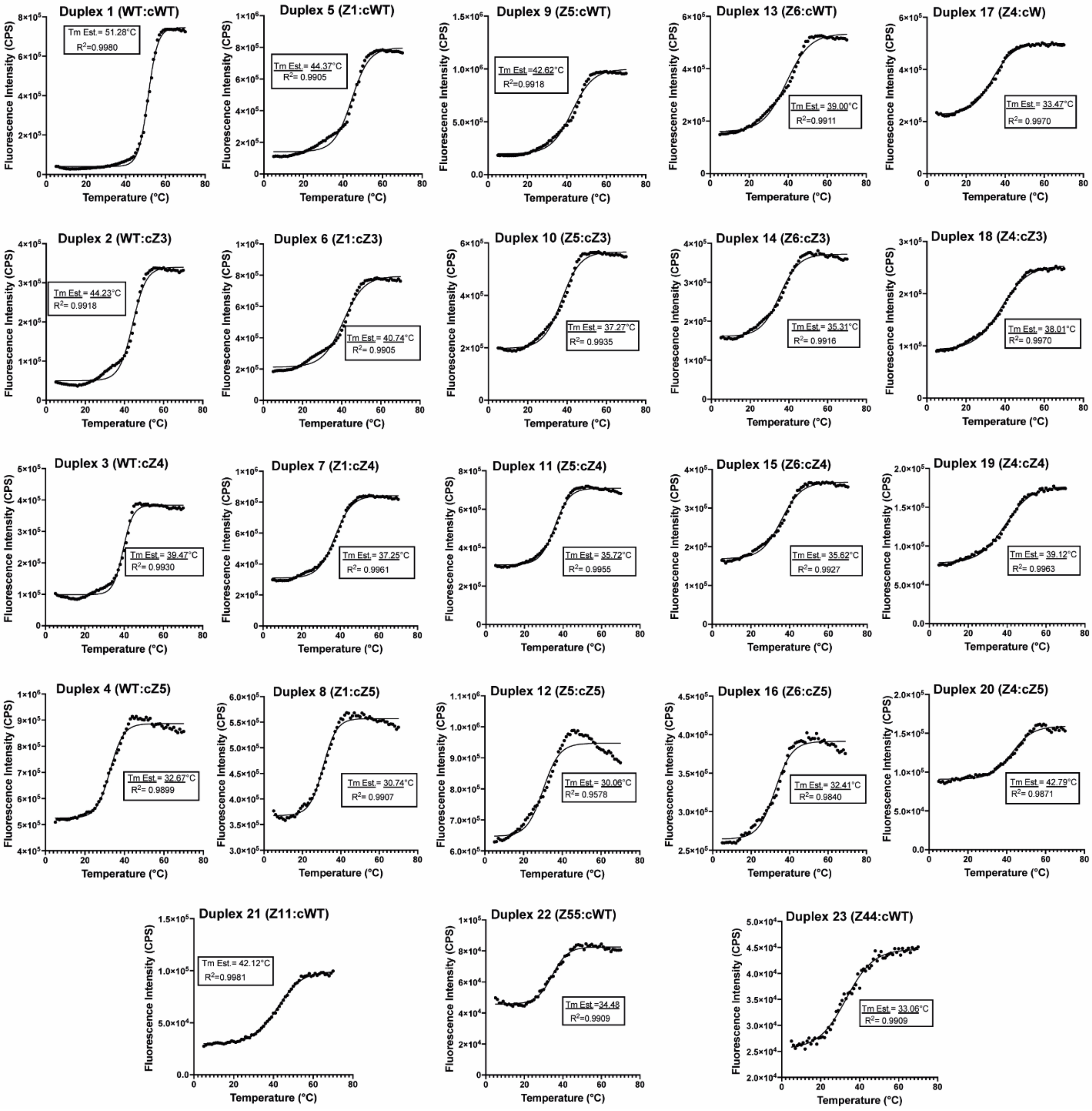

Figure S2. Thermal analysis of ZImera. Tm measurements were recorded in a quartz cuvette with a 3 mm pathlength. The temperature was varied in 1°C increments across a range from 5 °C to 70 °C.

4. FRET analysis

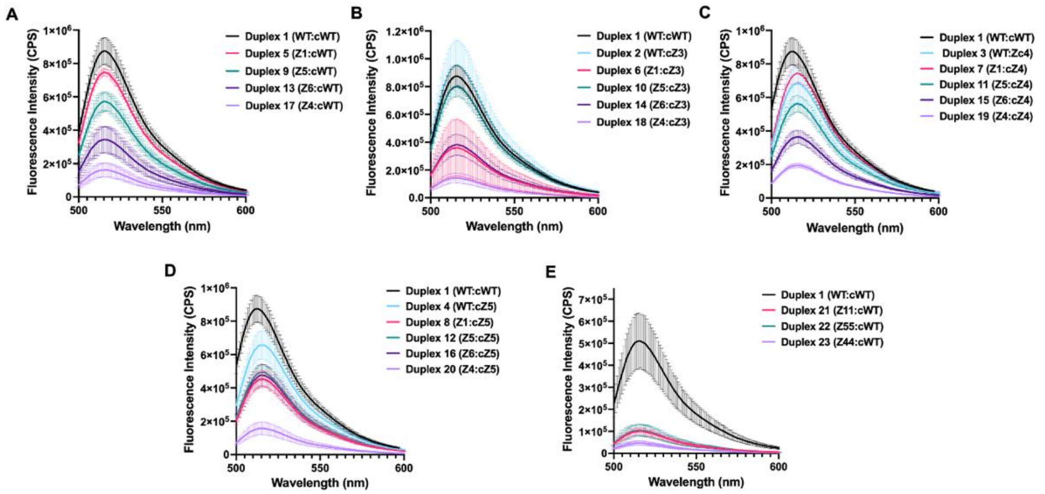

Figure S3. Analysis of DNase 1 sensitivity of ZImera. (A) Fluorescence emission spectra of cWT duplexes hybridized with propyl-linked indole-modified complementary strands. (B–D) Fluorescence emission spectra of cZ3, cZ4, and cZ5 duplexes, respectively, hybridized with propyl-linked indole-modified complementary strands. (E) Fluorescence emission spectra of cWT duplexes hybridized with propargyl-linked indole-modified complementary strands. All spectra include a control WT duplex (Duplex 1) with DNase I treatment. DNase I sensitivity of ZImera duplexes was assessed by preparing 10 µmol DNA samples in a total volume of 10 µL. Samples were treated with 1.4 units of DNase I (Thermo Scientific, Cat# EN0521, 0.56 U/µL) for 15 minutes at 37 °C. Fluorescence measurements were recorded at 20 °C, over a wavelength range of 500–600 nm.

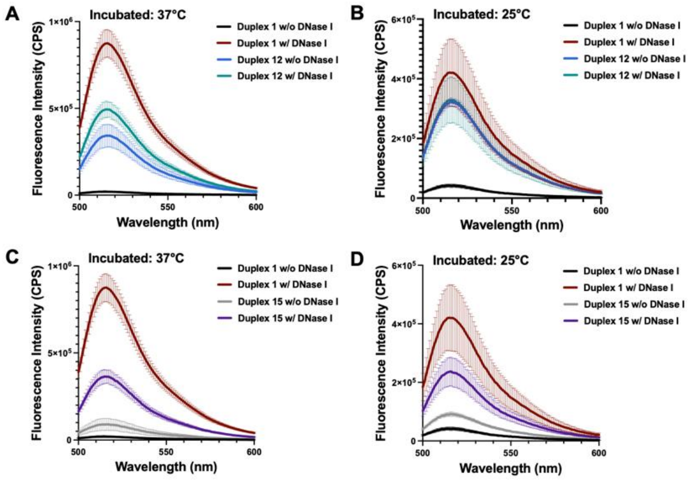

Figure S4. **DNase I sensitivity of duplexes 12 and 15 at 25 °C.** (A, B) Fluorescence emission spectra comparing DNase I digested Duplex 1 and Duplex 12 at 37 °C and 25 °C, respectively. (C, D) Fluorescence emission spectra comparing DNase I-digested Duplex 1 and Duplex 15 at 37 °C and 25 °C, respectively. For the 25 °C digestion reactions shown in panels (B) and (D), both untreated and DNase I-treated samples were incubated overnight at 25 °C, following the same preparation method as the 37 °C assays. Fluorescence measurements were recorded at 20 °C, over a wavelength range of 500–600 nm

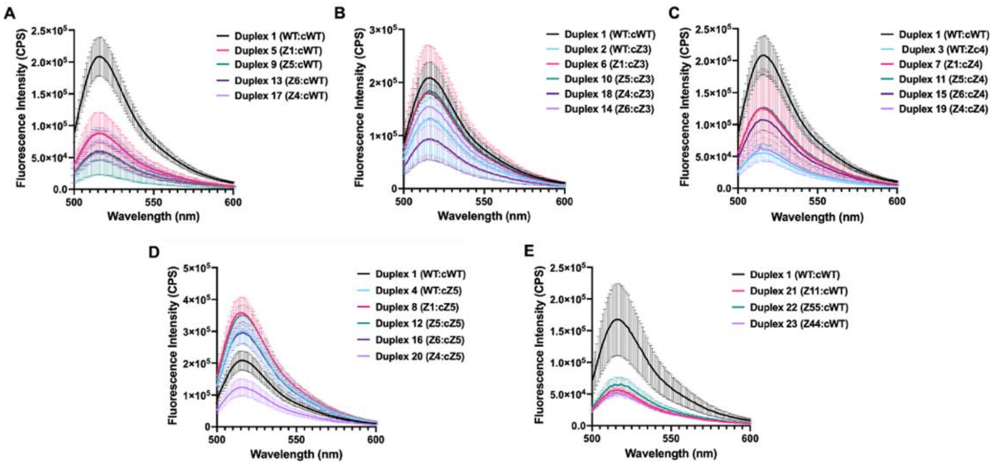

Figure S5. **Analysis of EcoRI sensitivity of ZImera.** (A) Fluorescence emission spectra of cWT duplexes hybridized with propyl-linked indole-modified complementary strands. (B–D) Fluorescence emission spectra of cZ3, cZ4, and cZ5 duplexes, respectively, hybridized with propyl-linked indole-modified complementary strands. (E) Fluorescence emission spectra of cWT duplexes hybridized with propargyl-linked indole-modified complementary strands. Each plot includes the WT control duplex (Duplex 1). EcoRI-HF activity on ZImera duplexes was assessed by preparing 10 µmol DNA samples in a 10 µL volume. Samples were treated with 70 units of EcoRI-HF (NEB, Cat# R3101M, 28 U/µL) and incubated overnight at 37 °C. Fluorescence measurements were performed at 20 °C, over a wavelength range of 500–600 nm.

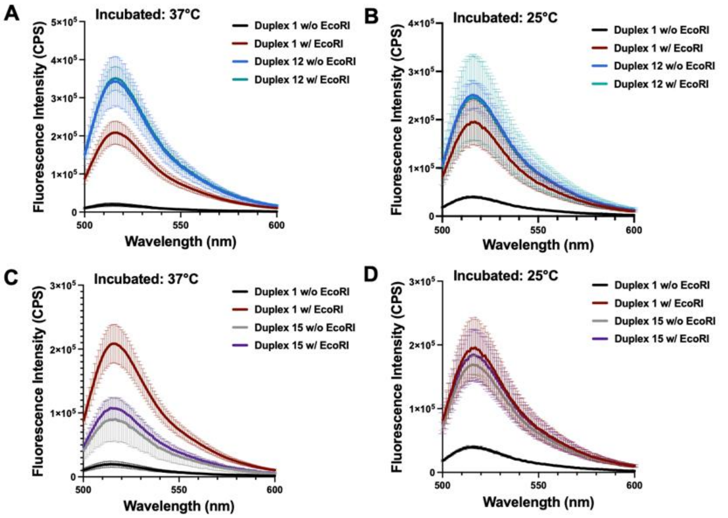

Figure S6. **Analyses of EcoRI sensitivity of duplexes 12 and 15 at 25 °C.** (A, B) Fluorescence emission spectra comparing DNase I-digested Duplex 1 and Duplex 12 at 37 °C and 25 °C, respectively. (C, D) Fluorescence emission spectra comparing DNase I-digested Duplex 1 and Duplex 15 at 37 °C and 25 °C, respectively. For the 25 °C digestion reactions shown in panels (B) and (D), both untreated and DNase I-treated samples were incubated overnight at 25 °C. The same sample preparation protocol used for the 37 °C digestion assays was applied. Fluorescence measurements were performed at 20 °C, over a wavelength range of 500–600 nm.

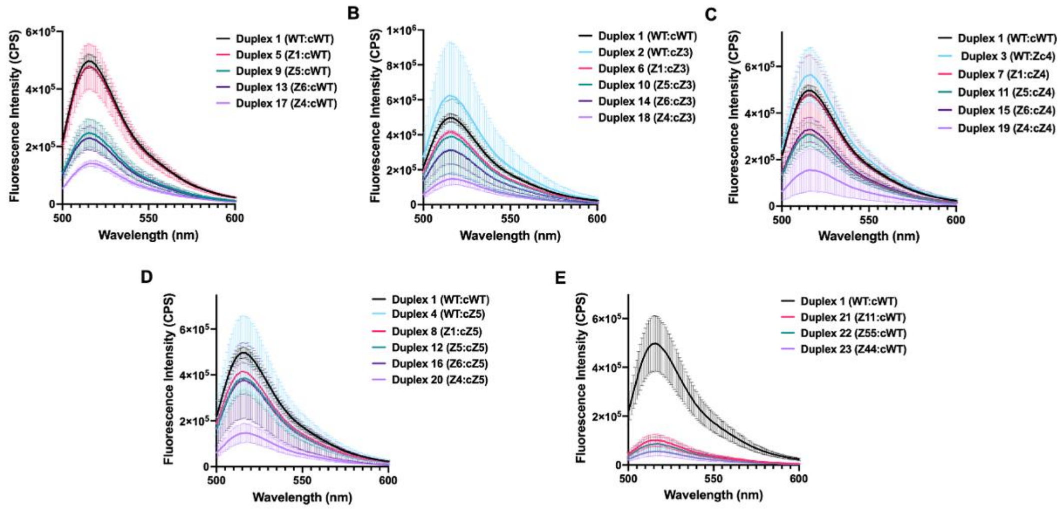

Figure S7. **Analysis of XmaI sensitivity of ZImera.** (A) Fluorescence emission spectra of cWT duplexes hybridized with propyl-linked indole-modified complementary strands. (B–D) Fluorescence emission spectra of cZ3, cZ4, and cZ5 duplexes, respectively, hybridized with propyl-linked indole-modified complementary strands. (E) Fluorescence emission spectra of duplexes hybridized with propargyl-linked indole-modified complementary strands. Each plot includes the WT control duplex (Duplex 1). XmaI activity on ZImera duplexes was analyzed by preparing 10 µmol DNA samples in a total volume of 10 µL. Samples were treated with 7 units of XmaI (NEB, Cat# R0180S, 2.8 U/µL) and incubated overnight at 37 °C. Fluorescence measurements were performed at 20 °C over a wavelength range of 500–600 nm.

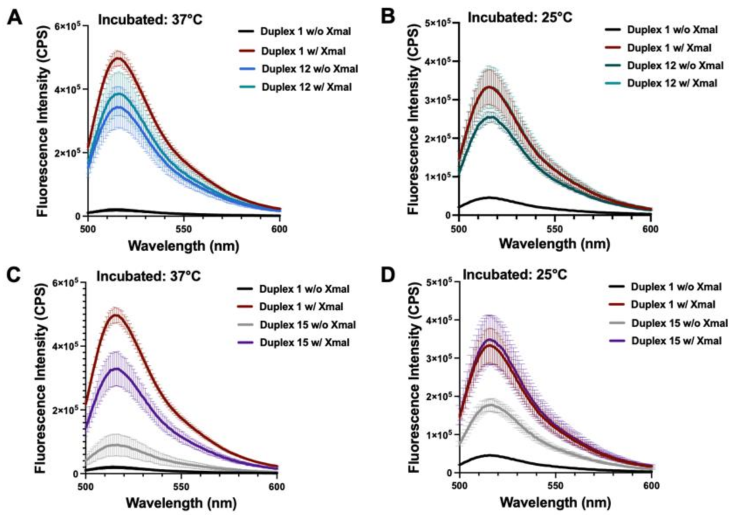

Figure S8. Analyses of XmaI sensitivity of Duplexes 12 and 15 at 25 °C. (A, B) Fluorescence emission spectra of DNase I-digested Duplex 1 and Duplex 12 at 37 °C and 25 °C, respectively, using cWT sequences. (C, D) Fluorescence emission spectra of DNase I-digested Duplex 1 and Duplex 15 at 37 °C and 25 °C, respectively, using cWT sequences. For the 25 °C digestion reactions shown in panels (B) and (D), both untreated and DNase I-treated samples were incubated overnight at 25 °C. Sample preparation followed the same protocol as used for the 37 °C digestion assays. Fluorescence measurements were performed at 20 °C over a wavelength range of 500–600 nm.

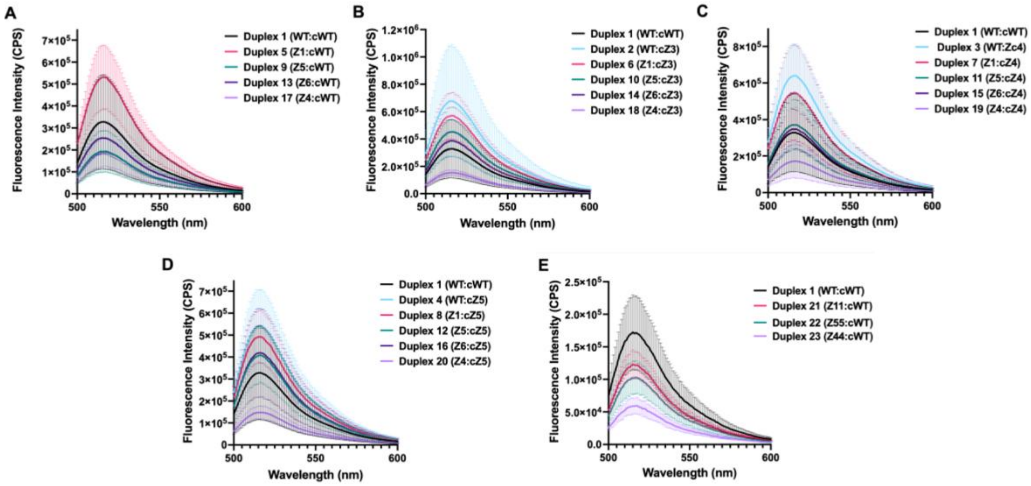

Figure S9. **Analysis of SmaI sensitivity of ZImera**. (A) Fluorescence emission spectra of cWT duplexes hybridized with propyl-linked indole-modified complementary strands. (B–D) Fluorescence emission spectra of cZ3, cZ4, and cZ5 duplexes, respectively, hybridized with propyl-linked indole-modified complementary strands. (E) Fluorescence emission spectra of duplexes hybridized with propargyl-linked indole-modified complementary strands. Each plot includes the WT control duplex (Duplex 1). SmaI activity on ZImera duplexes was analyzed by preparing 10 µmol DNA samples in a total volume of 10 µL. Samples were treated with 7 units of SmaI (Thermo Scientific, Cat# ER0665, 2.8 U/µL) and incubated overnight at 25 °C. Fluorescence measurements were performed at 20 °C over a wavelength range of 500–600 nm.

5. NMR Spectra:

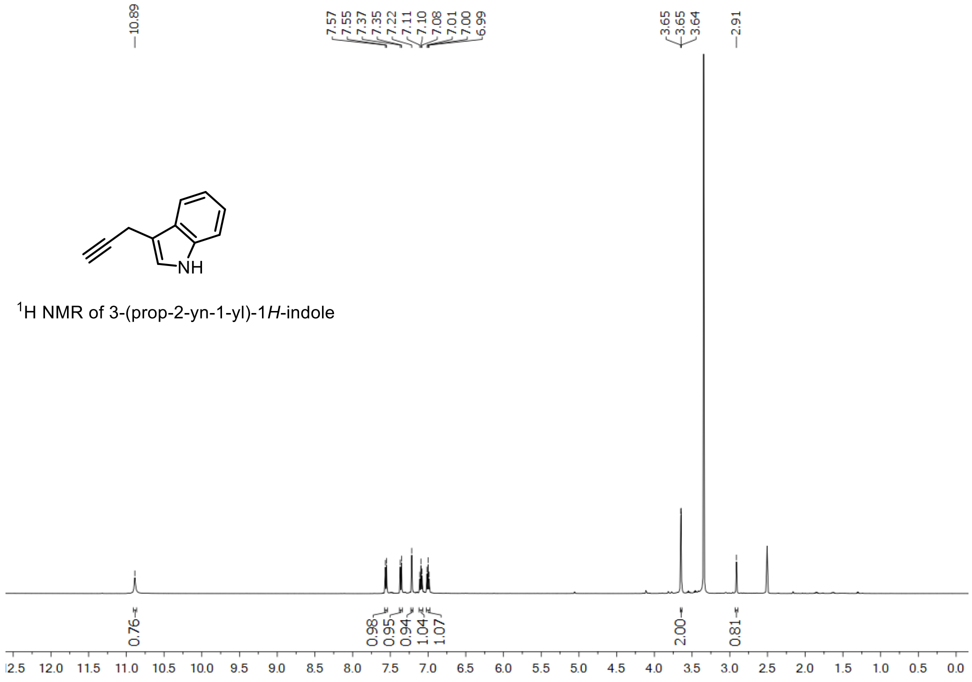

Figure S10 ^{1}H NMR spectrum of 3-(prop-2-yn-1-yl)-1*H*-indole (DMSO-*d6*)

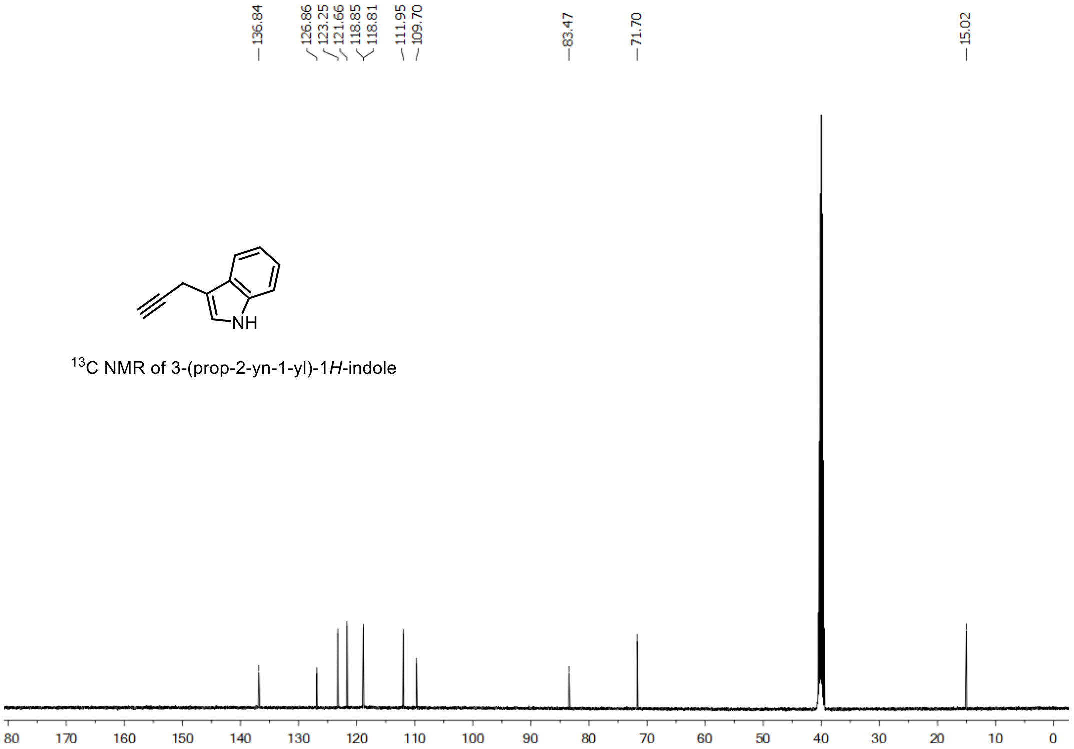

Figure S11 ^{13}C NMR spectrum of 3-(prop-2-yn-1-yl)-1*H*-indole (DMSO-*d6*)

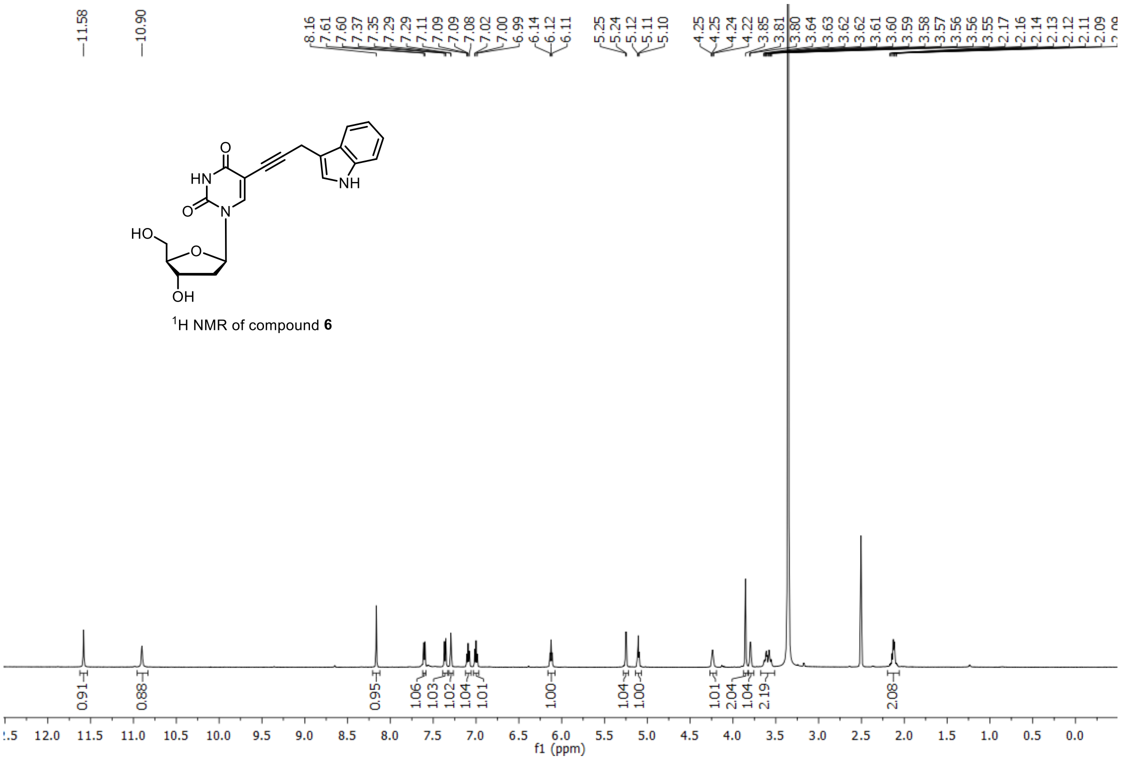

Figure S12 ^{1}H NMR spectrum of compound **6** (DMSO-*d6*)

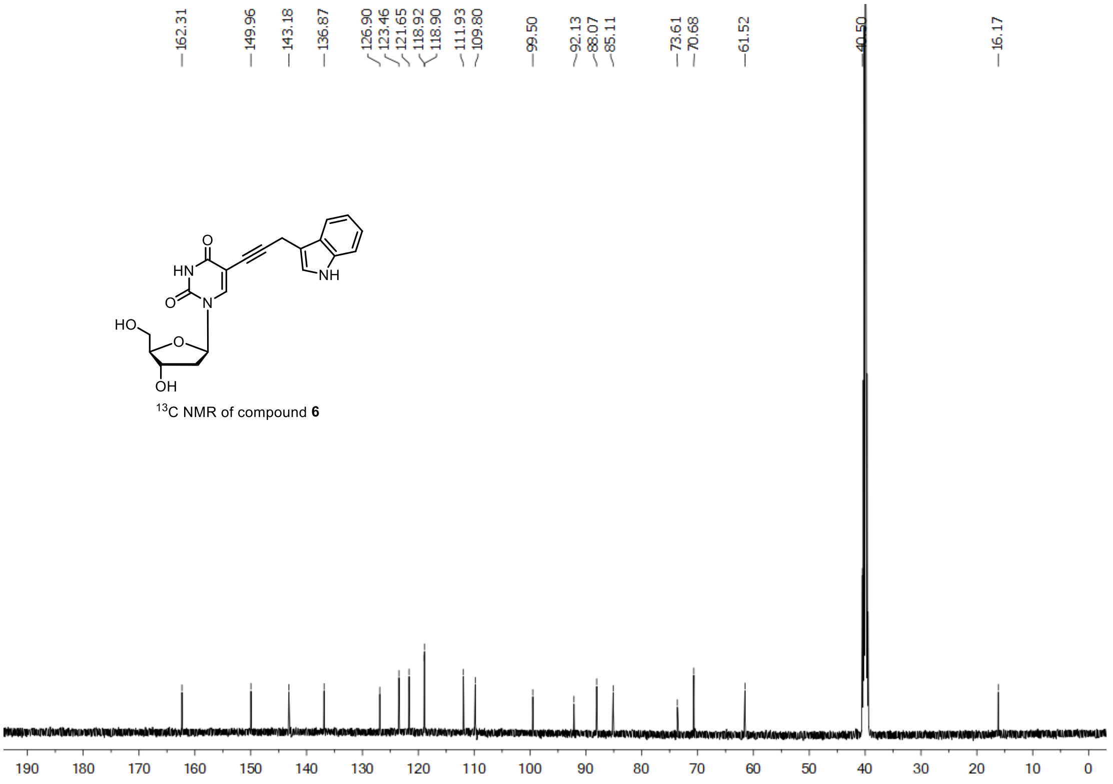

Figure S13 ^{13}C NMR spectrum of compound **6** (DMSO-*d6*)

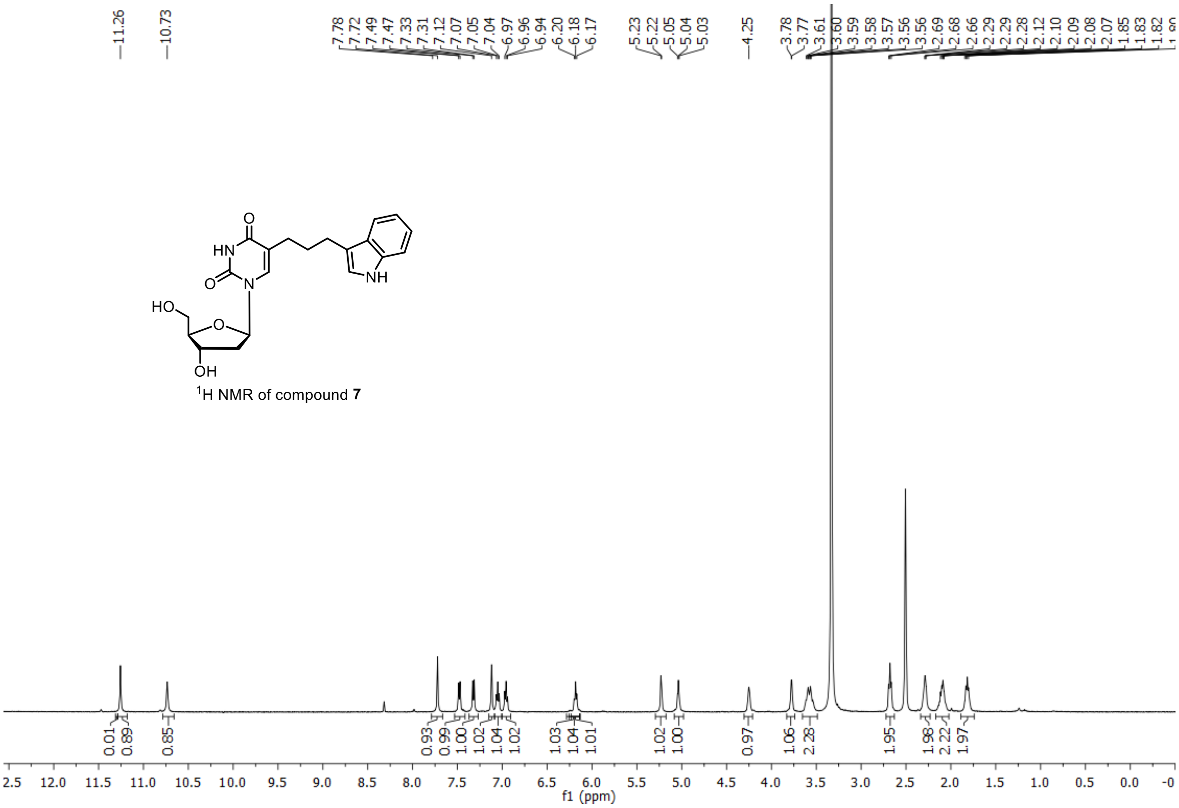

Figure S14 ^{1}H NMR spectrum of compound **7** (DMSO-*d6*)

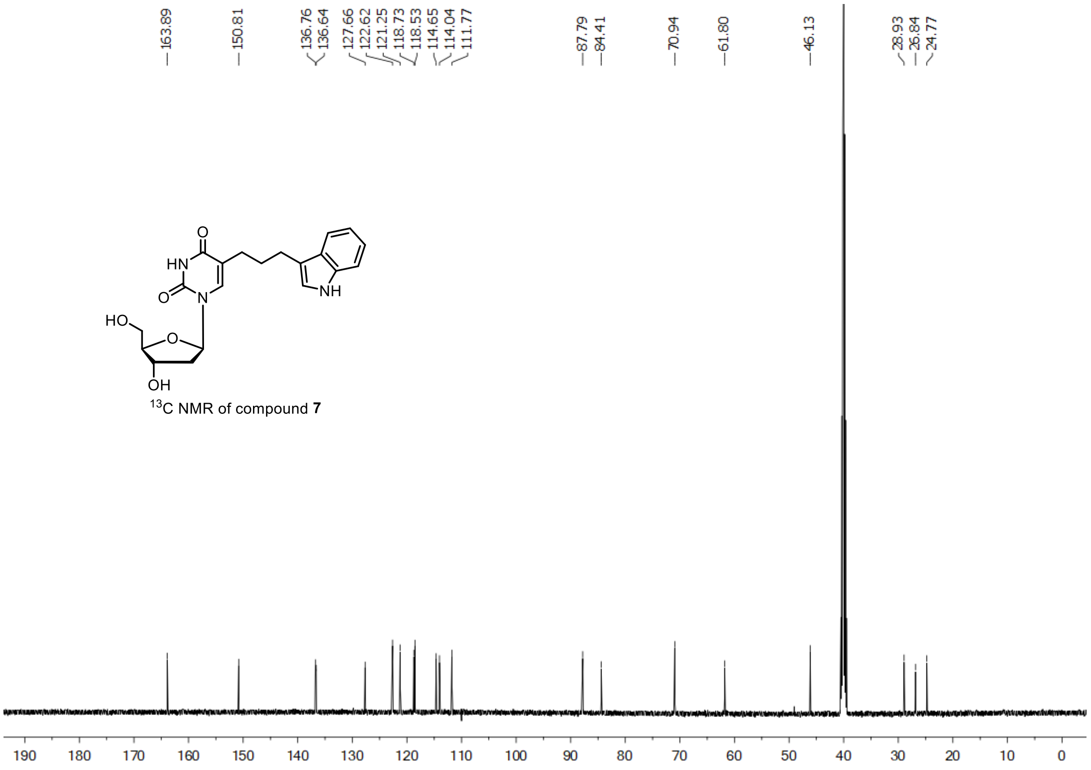

Figure S15 ^{13}C NMR spectrum of compound **7** (DMSO-*d6*)

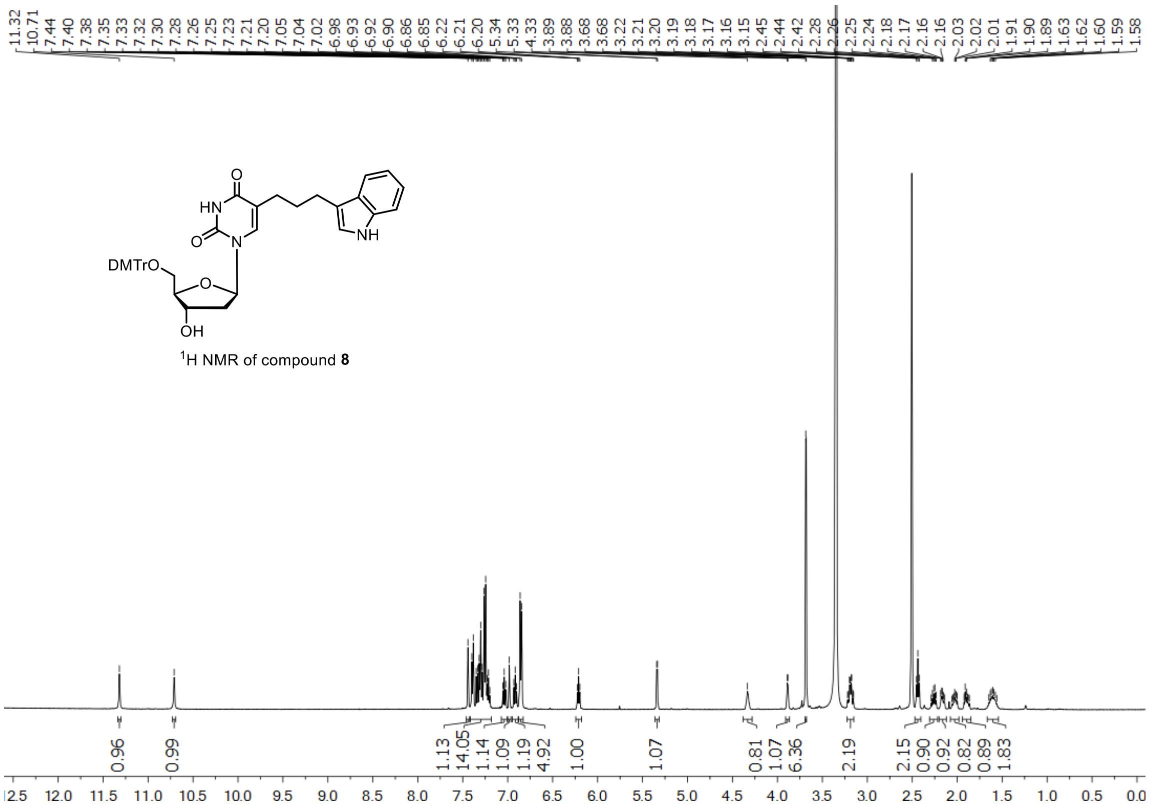

Figure S16. ^{1}H NMR spectrum of compound **8** (DMSO-*d6*)

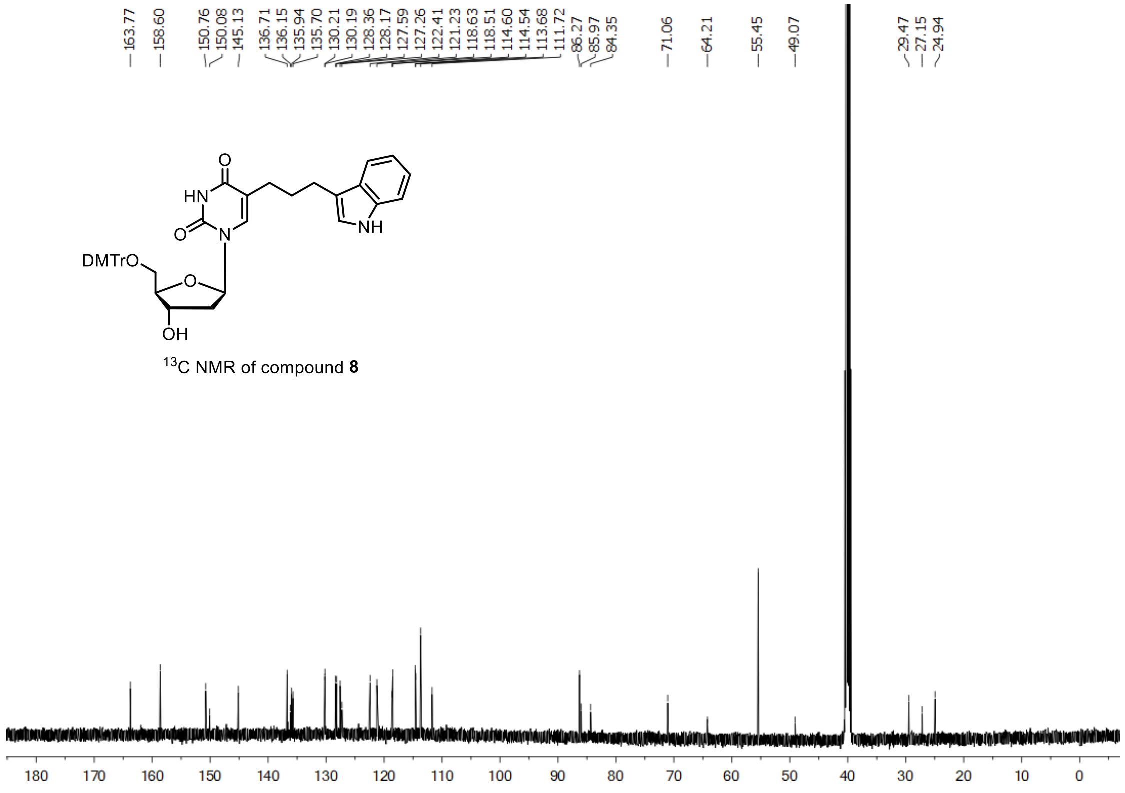

Figure S17. ^{13}C NMR spectrum of compound **8** (DMSO-*d6*)

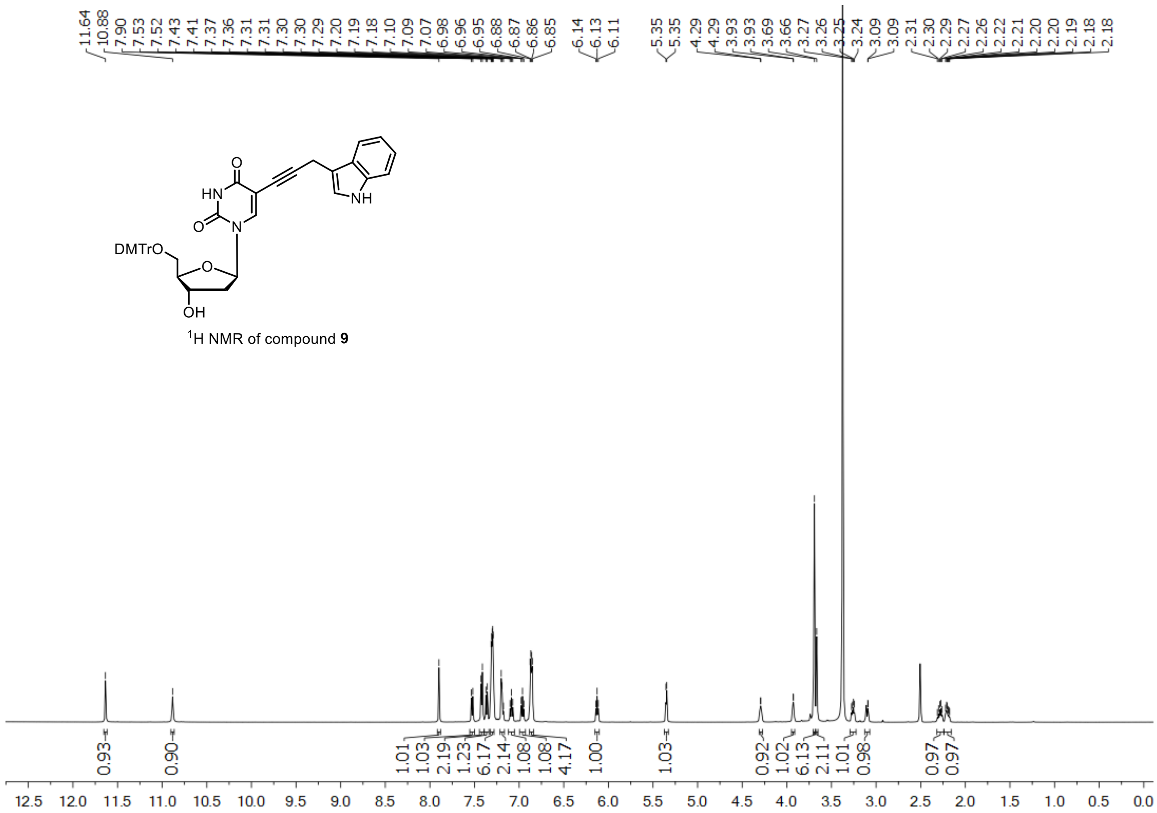

Figure S18 ^{1}H NMR spectrum of compound **9** (DMSO-*d6*)

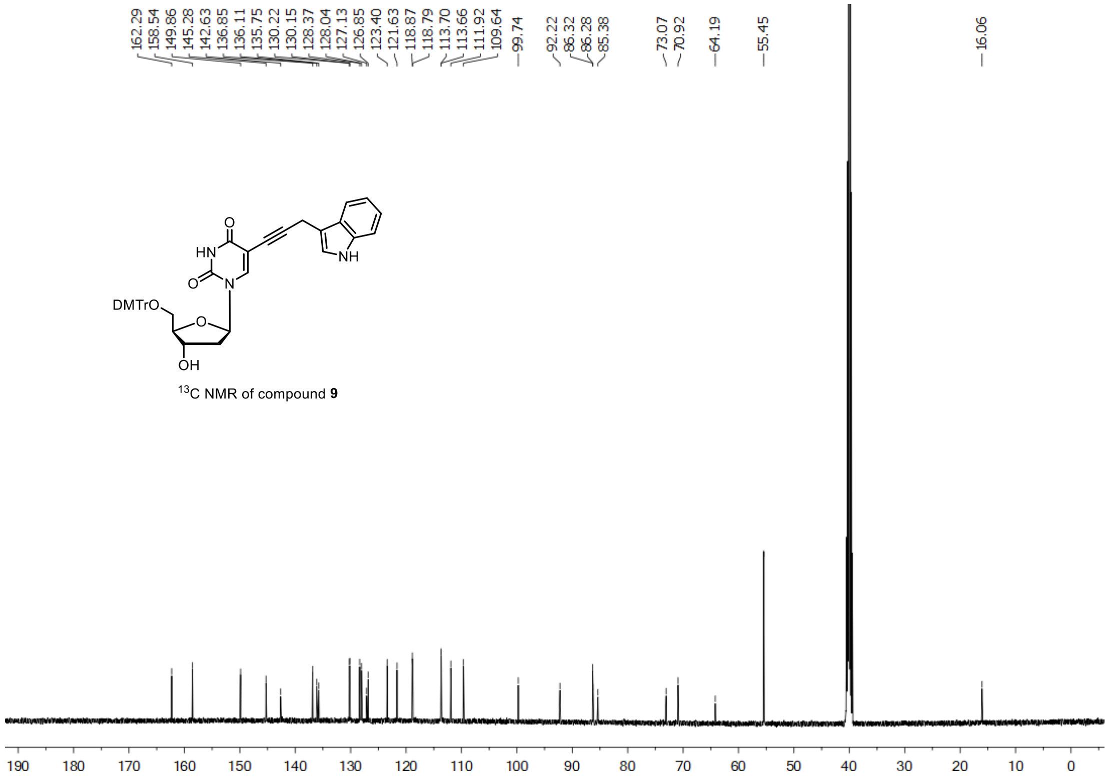

Figure S19 ^{13}C NMR spectrum of compound **9** (DMSO-*d6*)

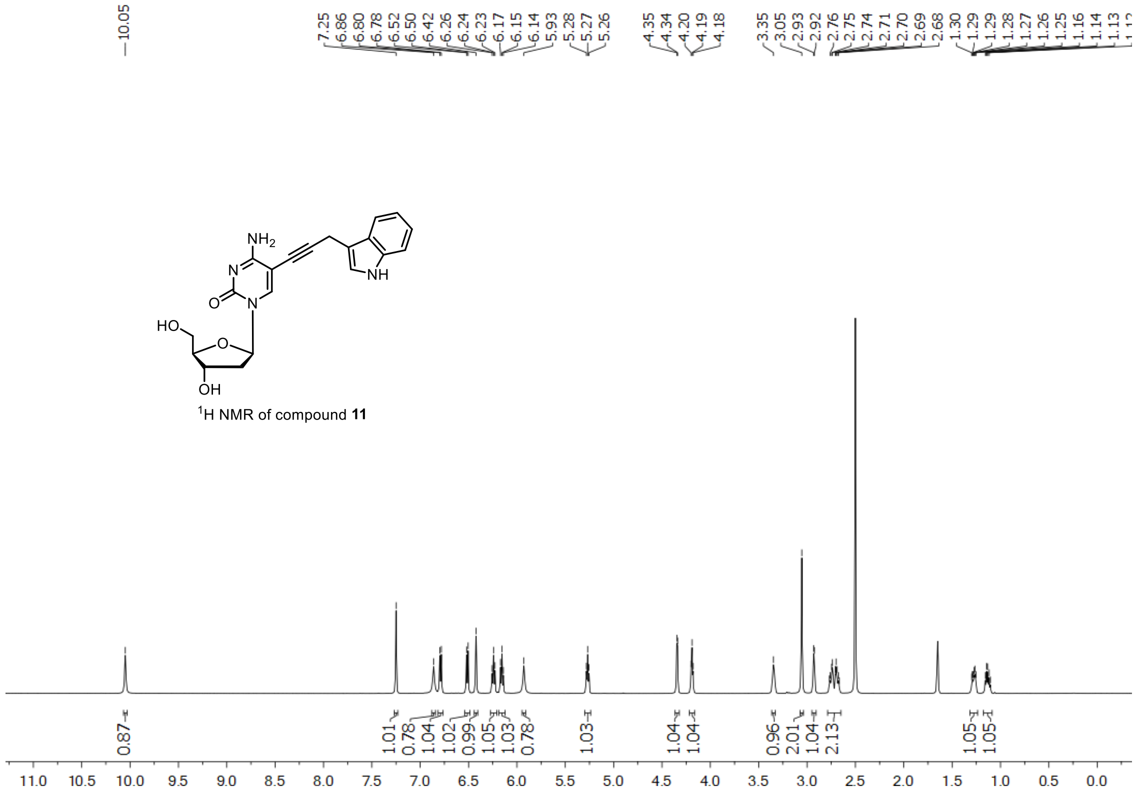

Figure S20 ^{1}H NMR spectrum of compound **11** (DMSO-*d6*)

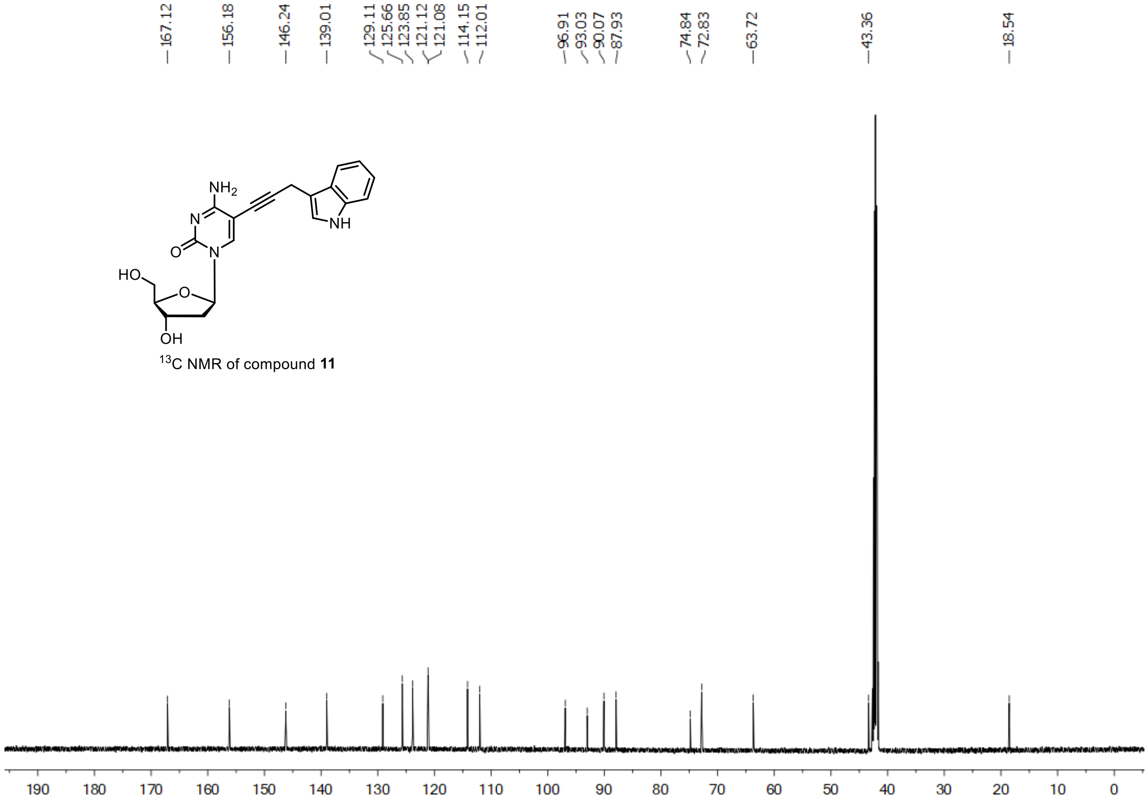

Figure S21 ^{13}C NMR spectrum of compound **11** (DMSO-*d6*)

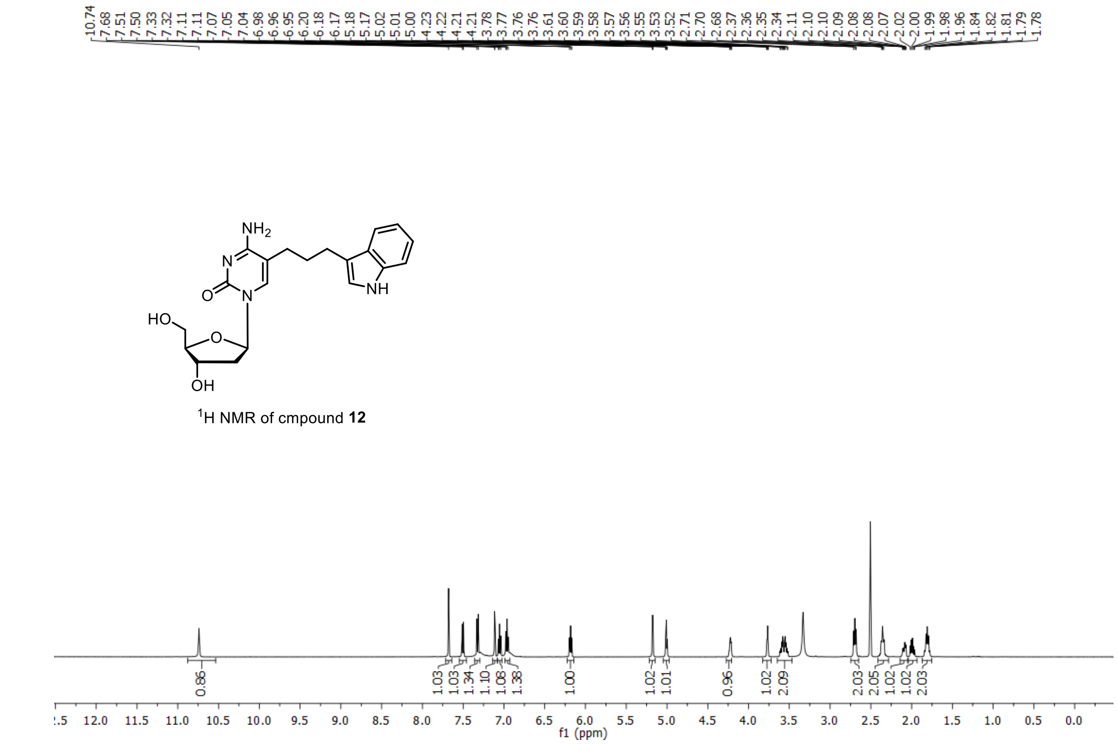

Figure S22 ^{1}H NMR spectrum of compound **12** (DMSO-*d6*)

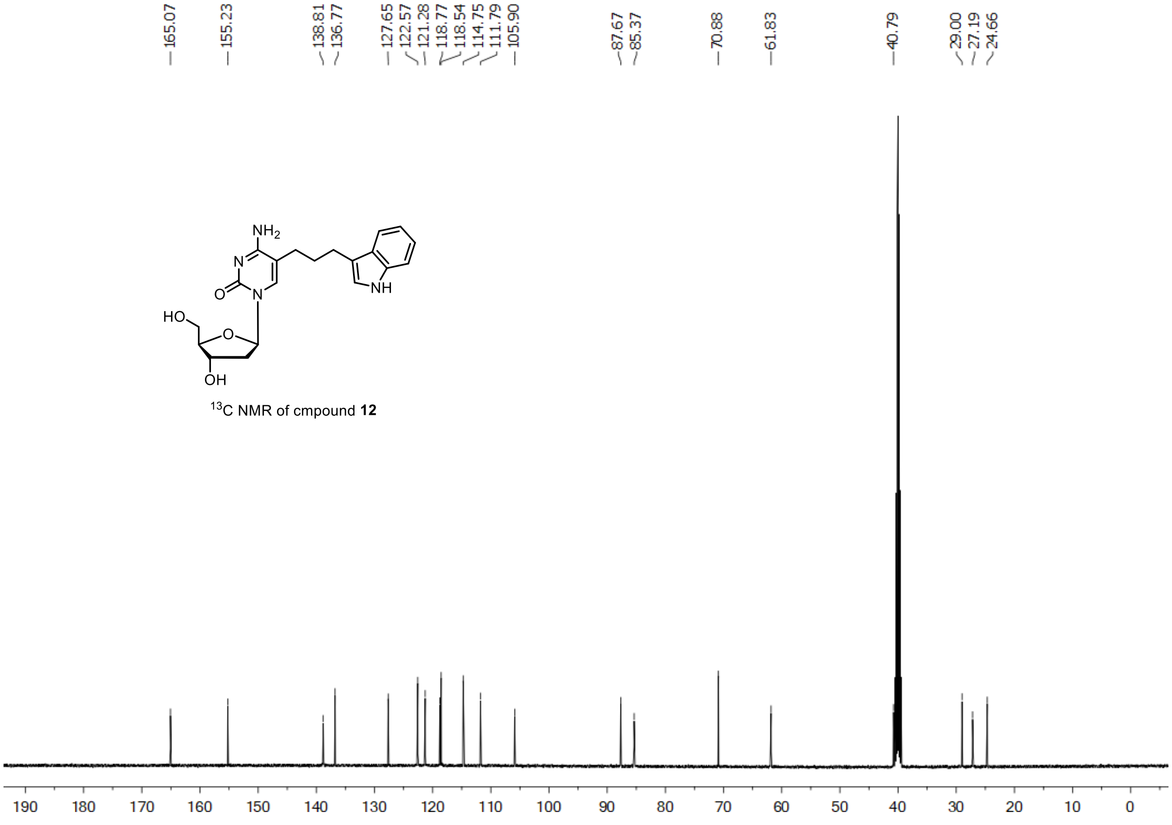

Figure S23 ^{13}C NMR spectrum of compound **12** (DMSO-*d6*)

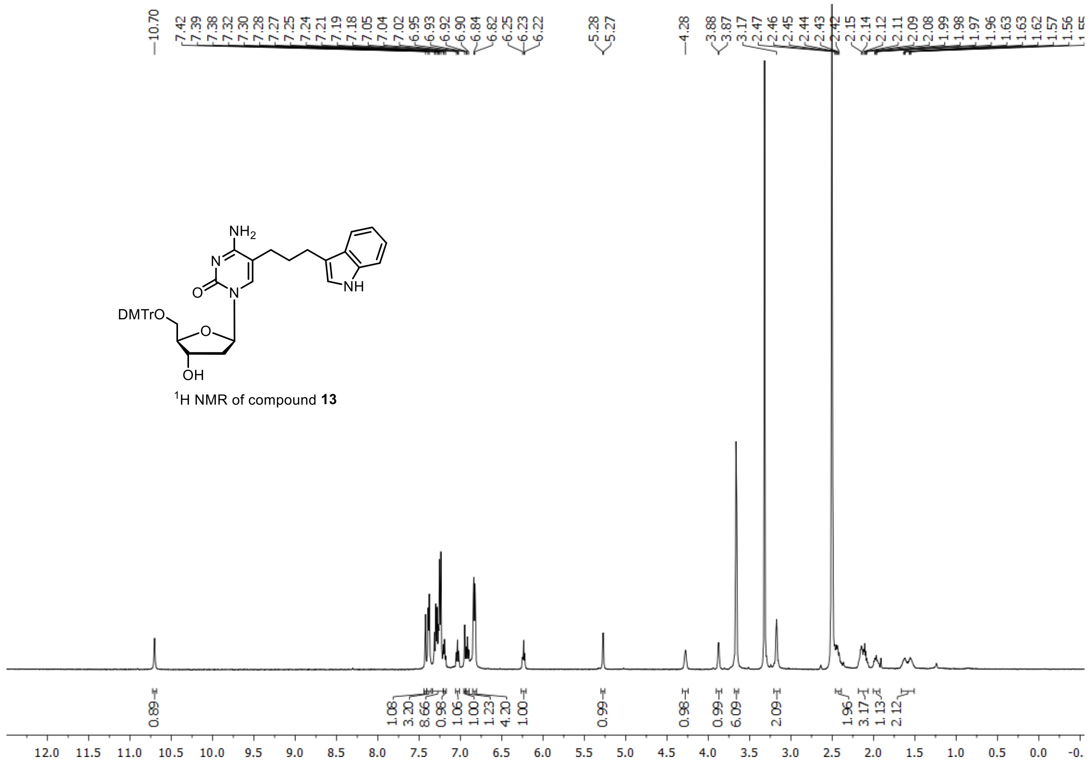

Figure S24 ^{1}H NMR spectrum of compound **13** (DMSO-*d6*)

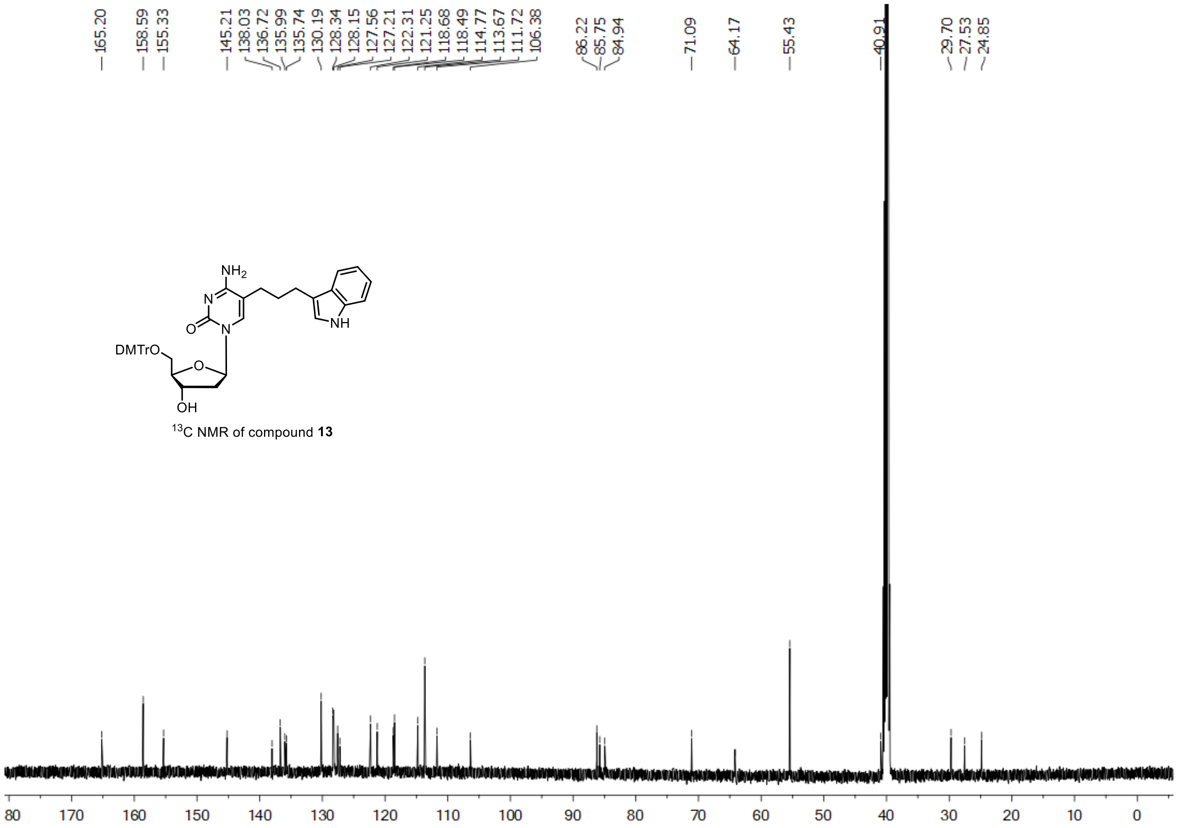

Figure S25 ^{13}C NMR spectrum of compound **13** (DMSO-*d6*)

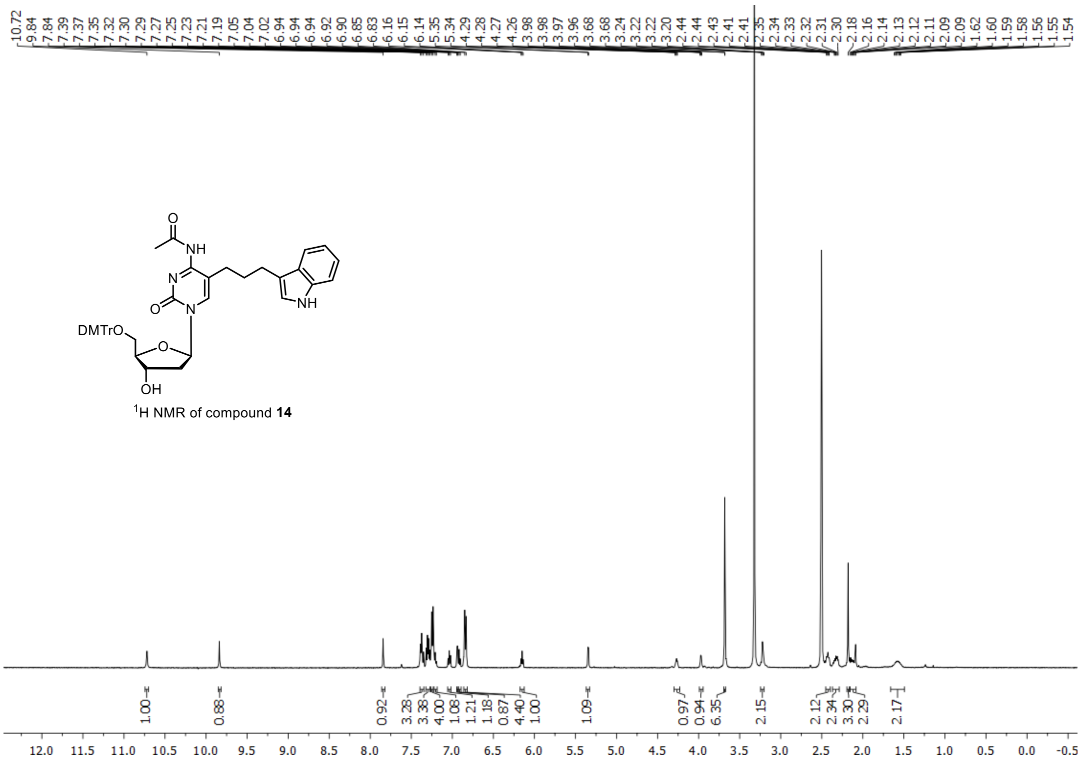

Figure S26 ^{1}H NMR spectrum of compound **14** (DMSO-*d6*)

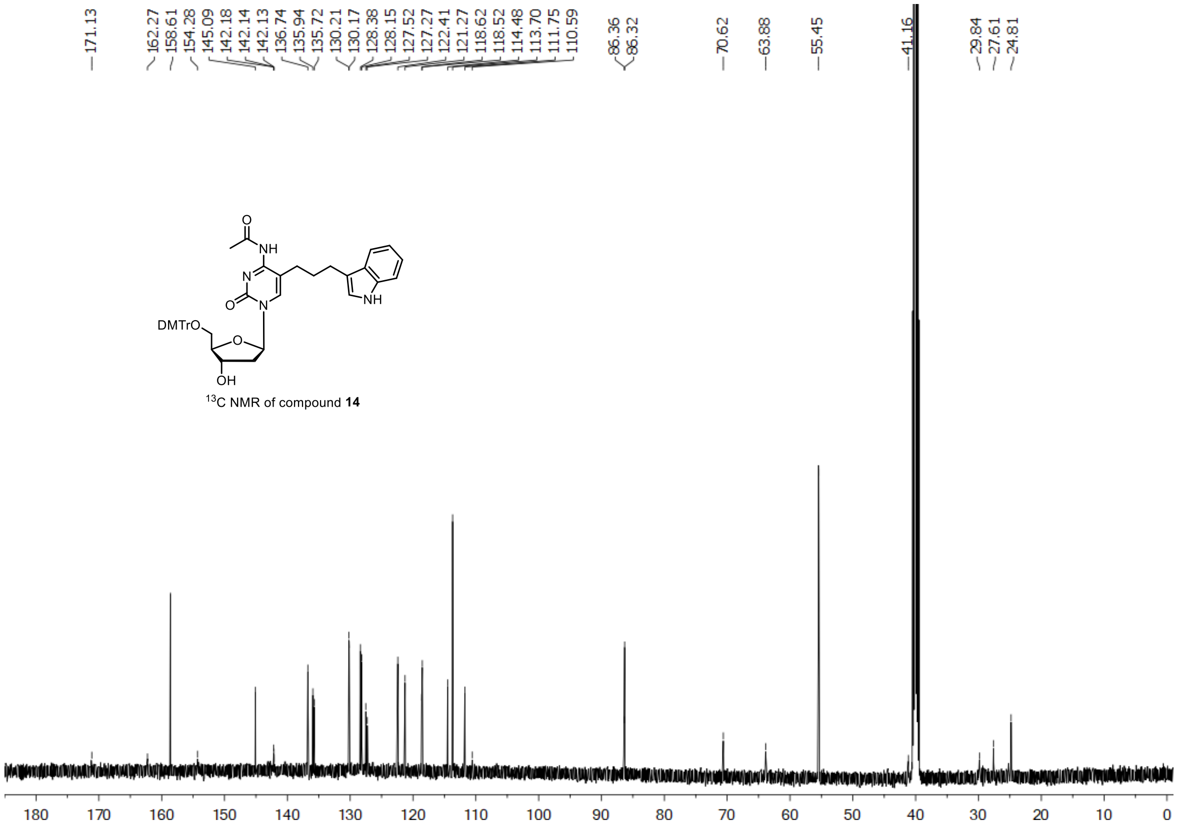

Figure S27. ^{13}C NMR spectrum of compound **14** (DMSO-*d6*)

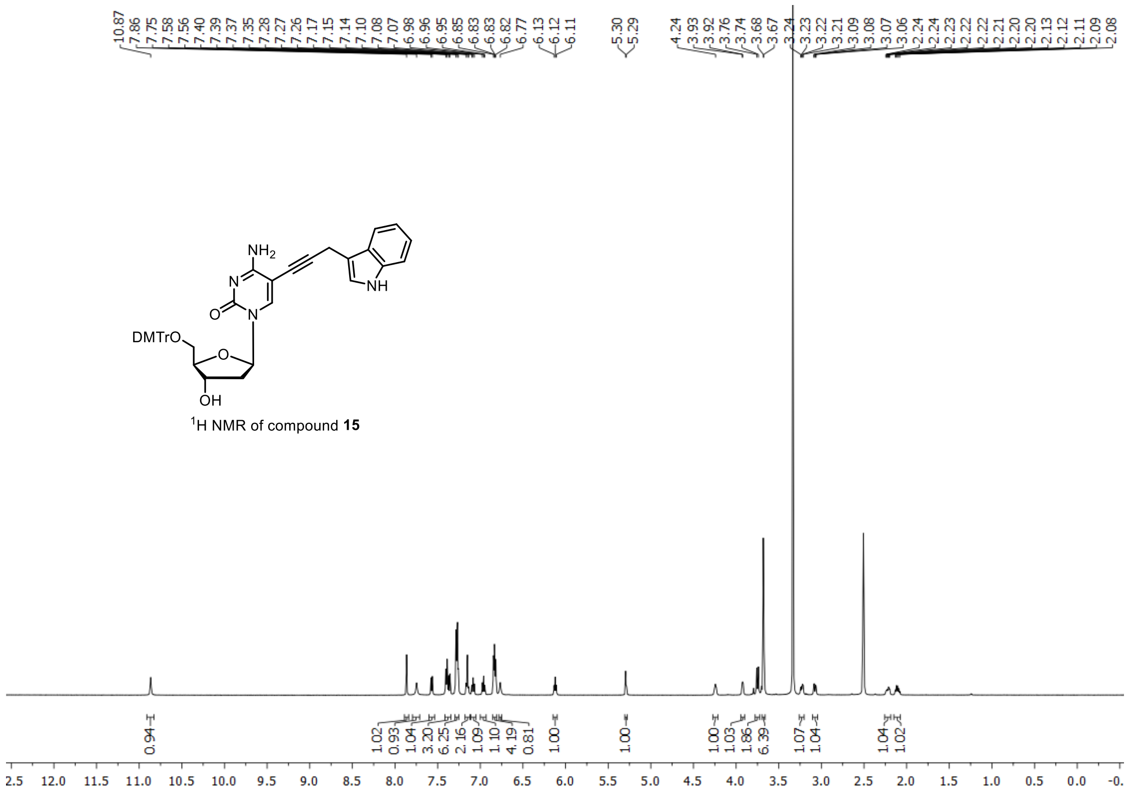

Figure S28 ^{1}H NMR spectrum of compound **15** (DMSO-*d6*)

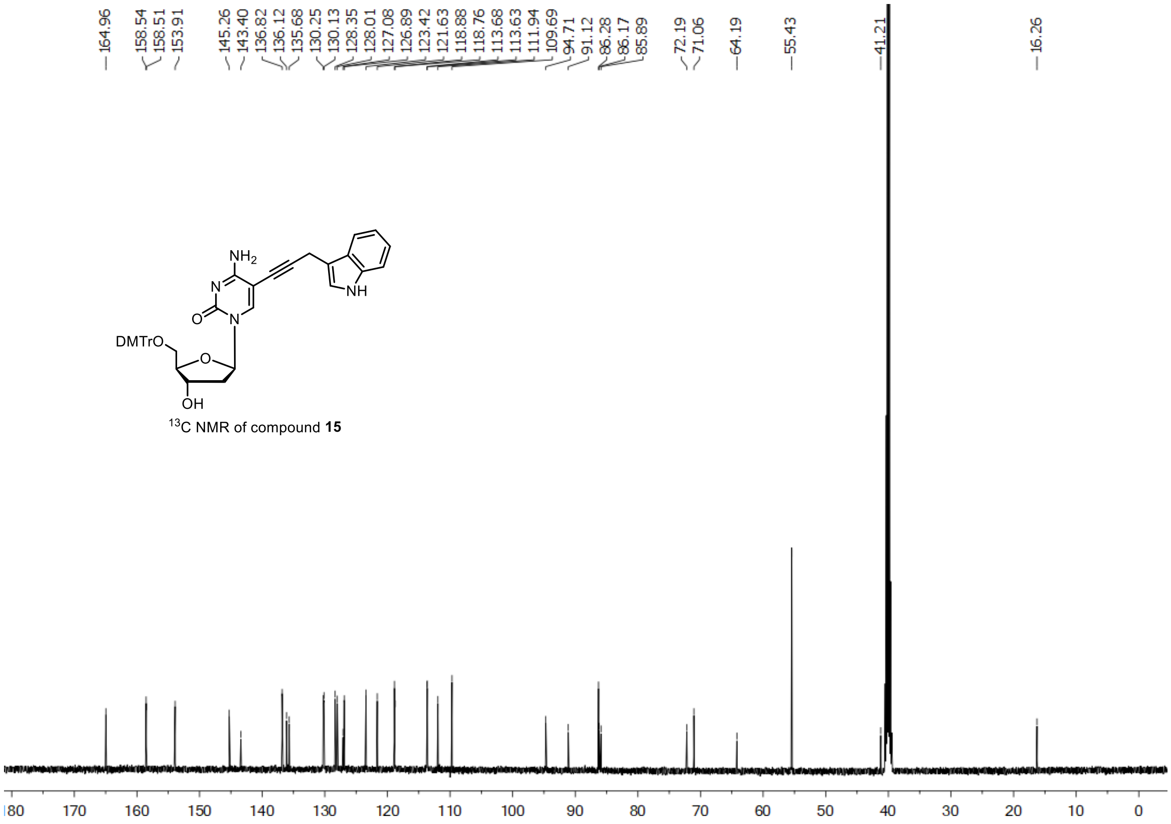

Figure S29 ^{13}C NMR spectrum of compound **15** (DMSO-*d6*)

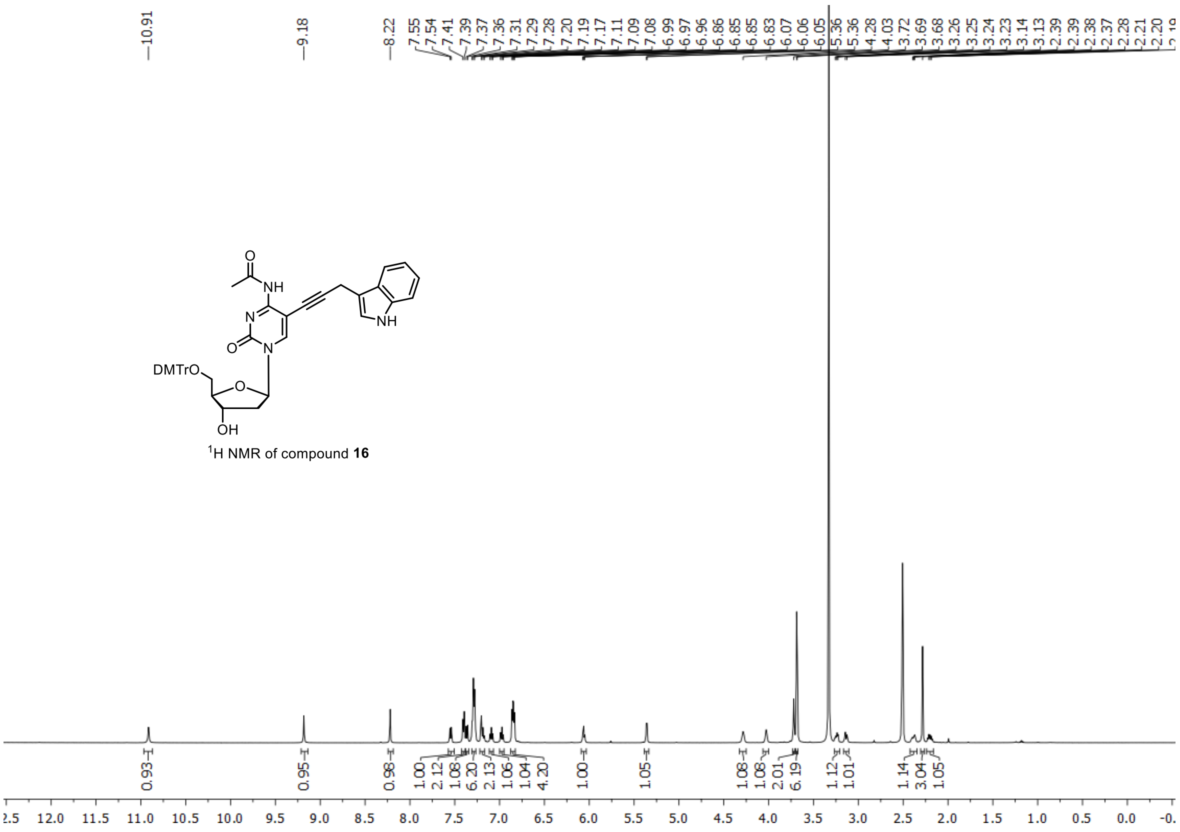

Figure S30. ^{1}H NMR spectrum of compound **16** (DMSO-*d6*)

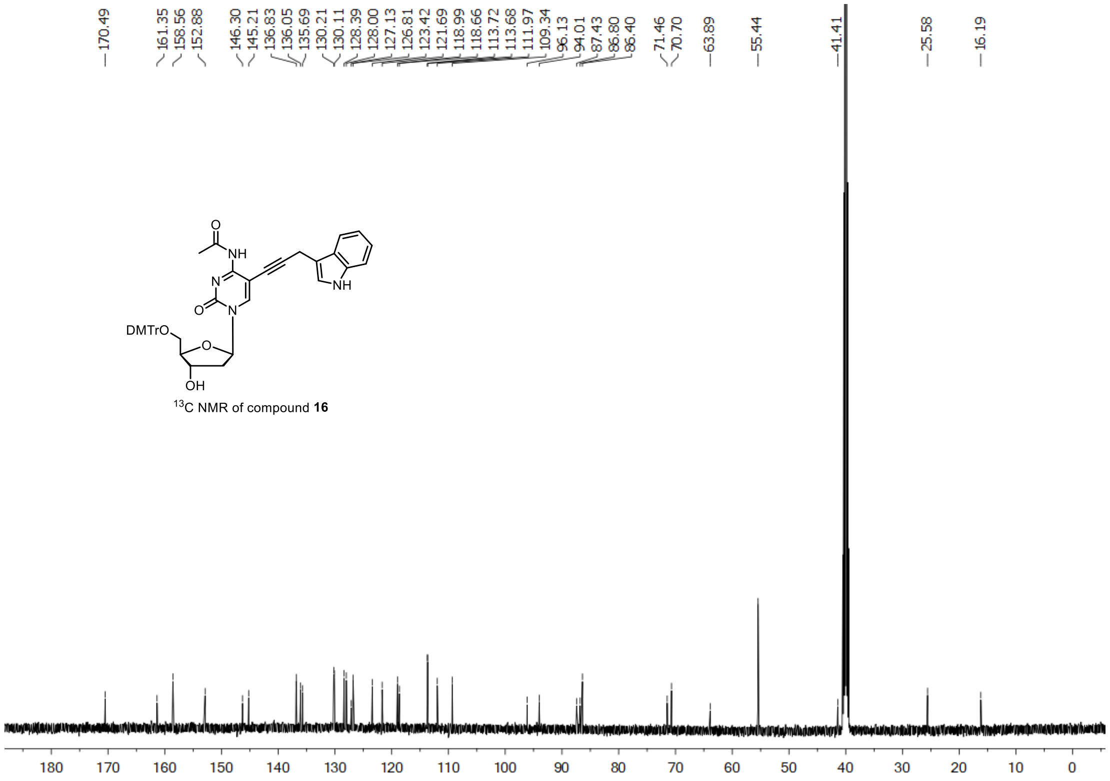

Figure S31. ^{13}C NMR spectrum of compound **16** (DMSO-*d6*)

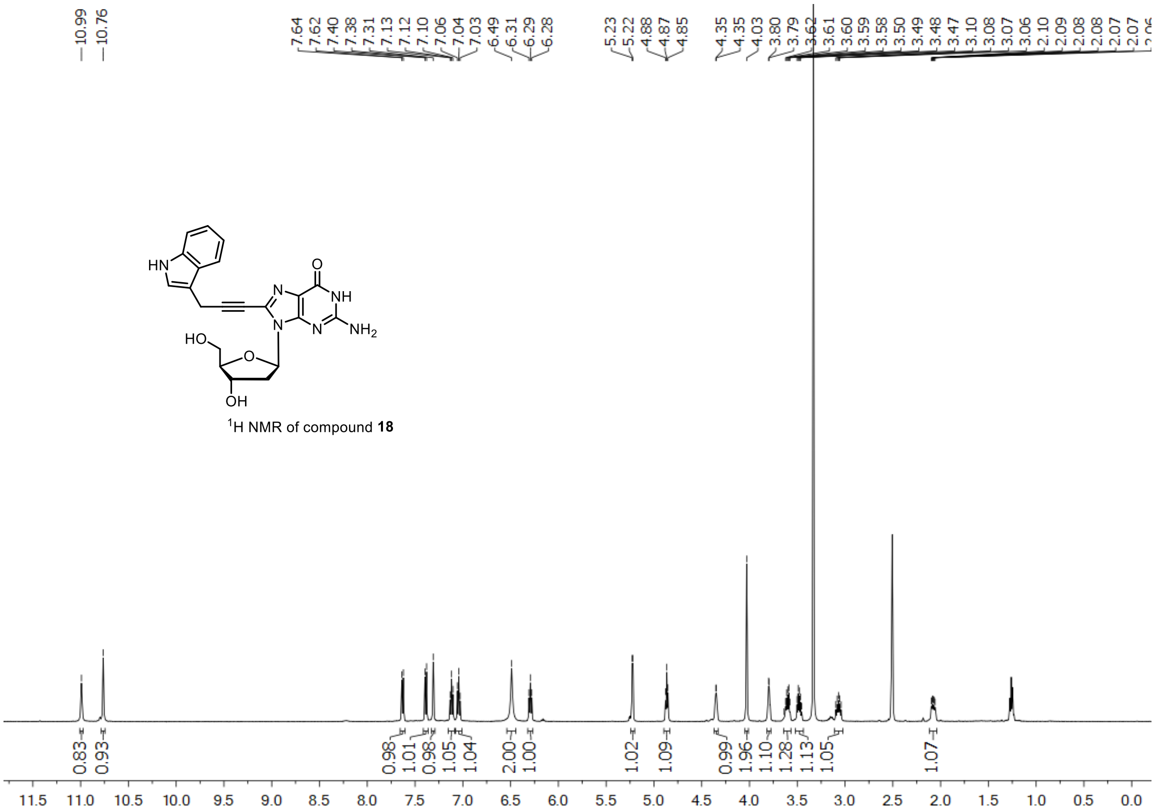

Figure S32. ^{1}H NMR spectrum of compound **18** (DMSO-*d6*)

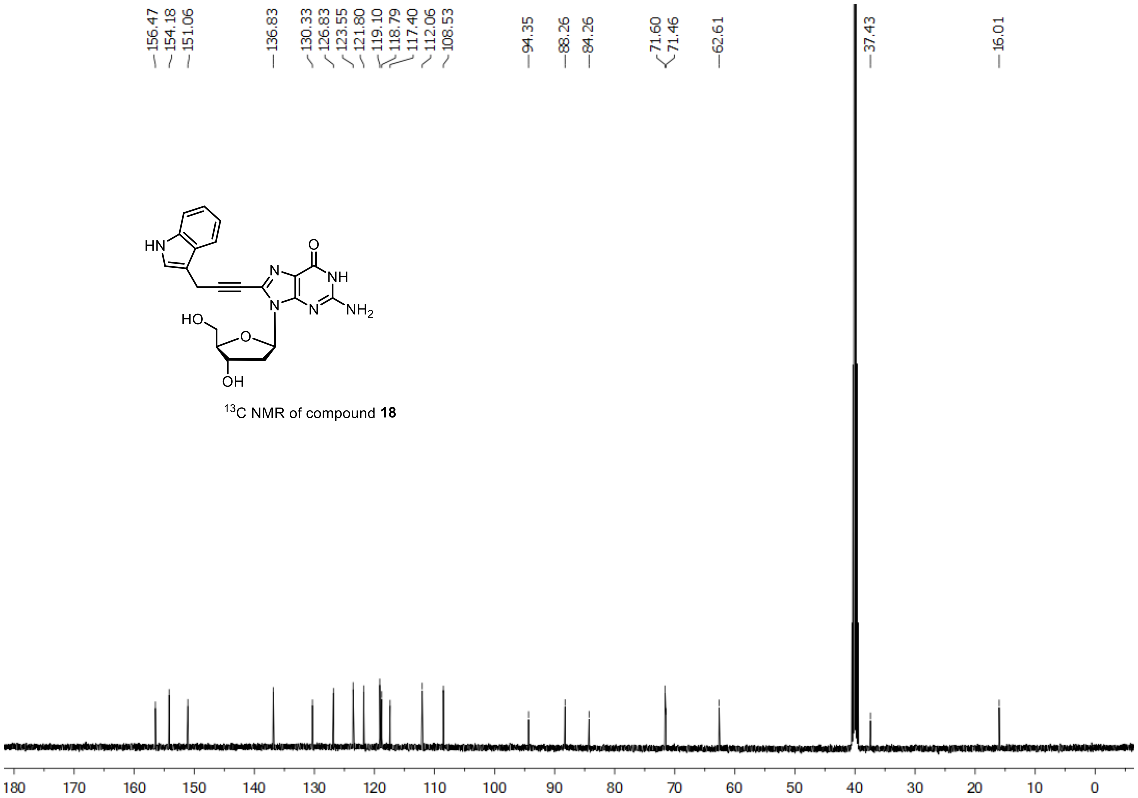

Figure S33 ^{13}C NMR spectrum of compound **18** (DMSO-*d6*)

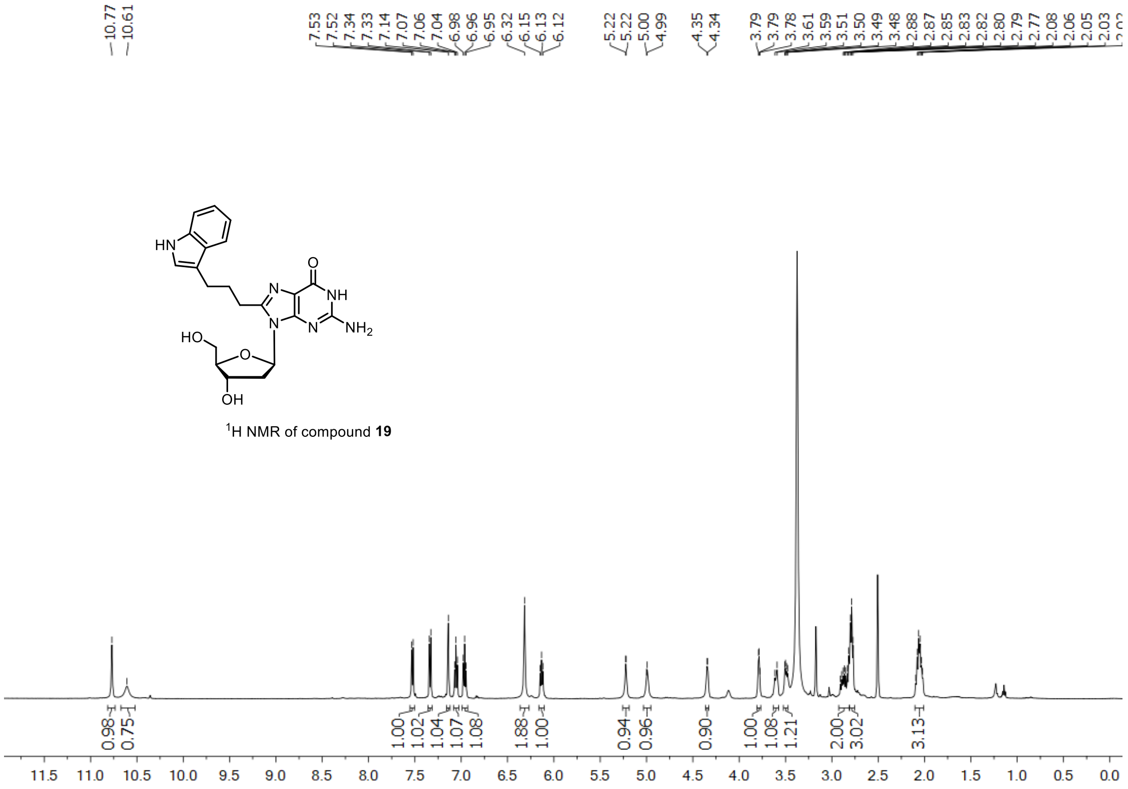

Figure S34 ^{1}H NMR spectrum of compound **19** (DMSO-*d6*)

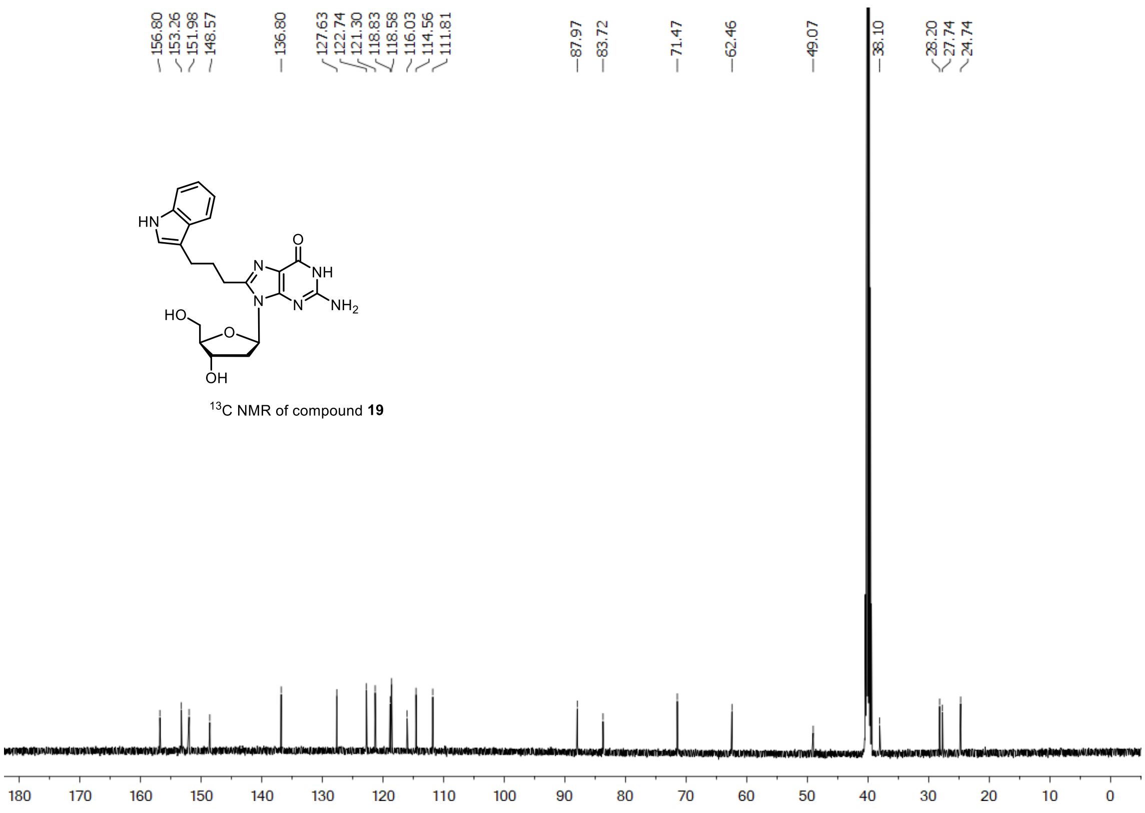

Figure S35 ^{13}C NMR spectrum of compound **19** (DMSO-*d6*)

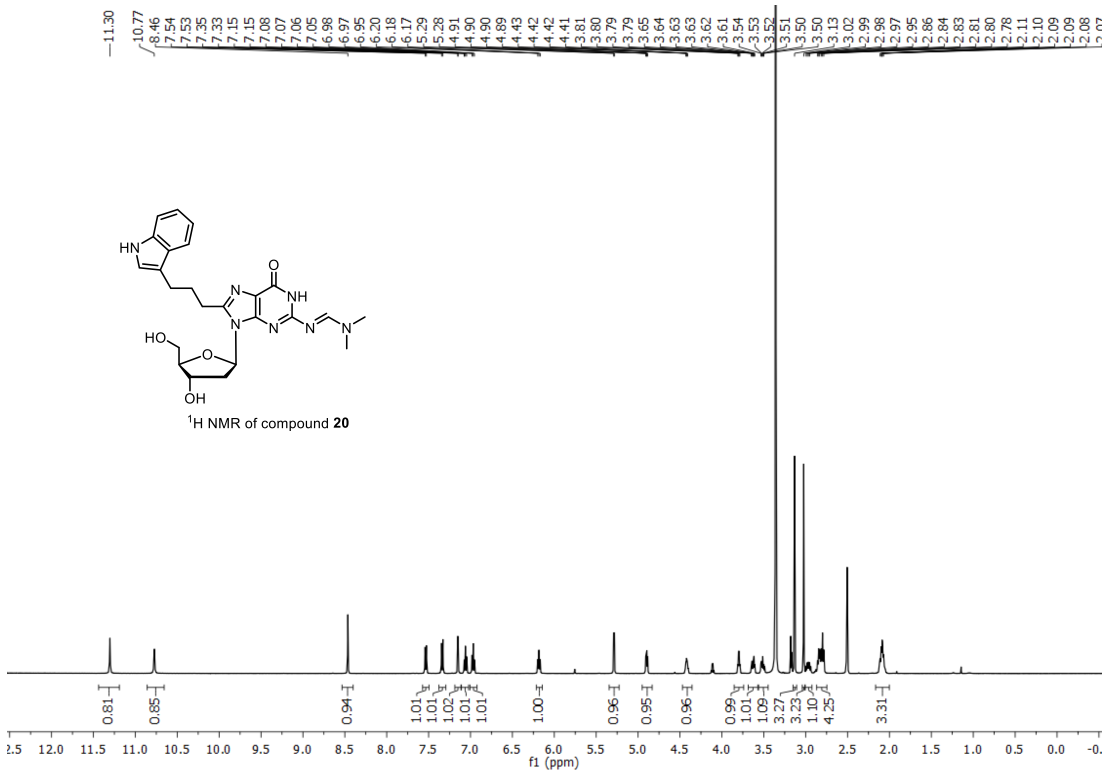

Figure S36 ^{1}H NMR spectrum of compound **20** (DMSO-*d6*)

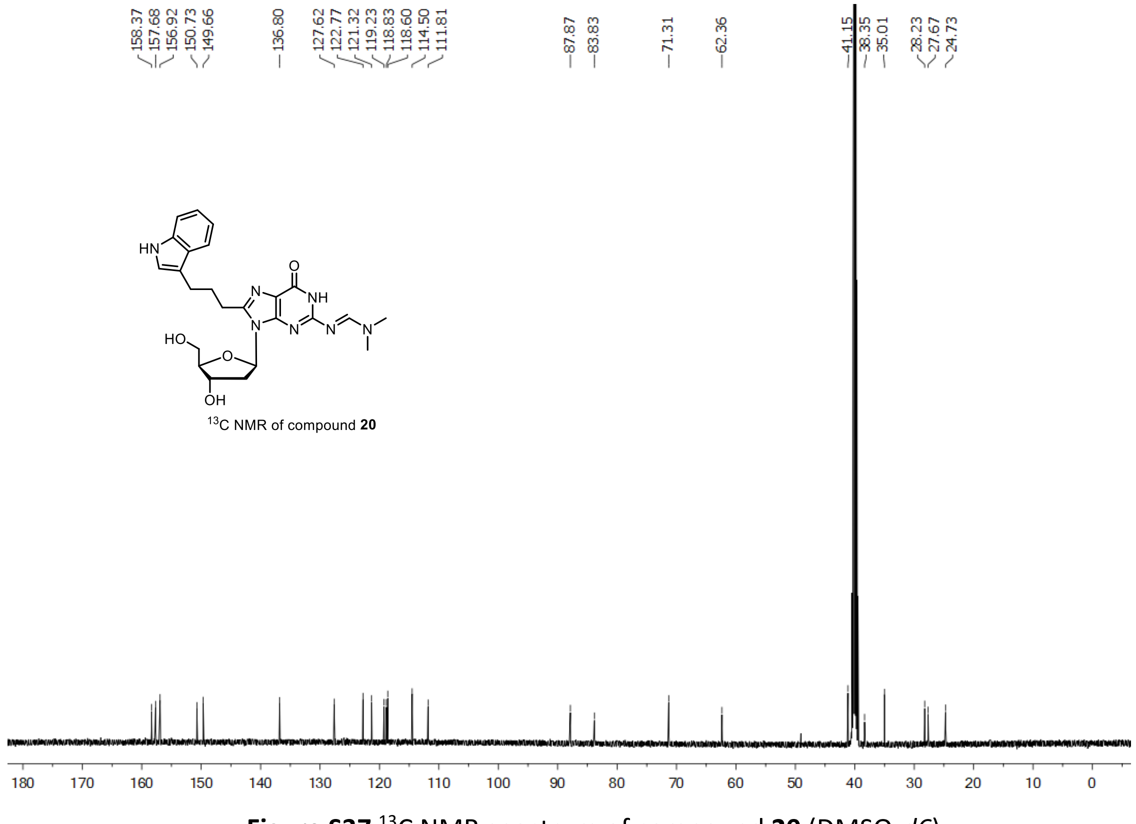

Figure S37 ^{13}C NMR spectrum of compound **20** (DMSO-*d6*)

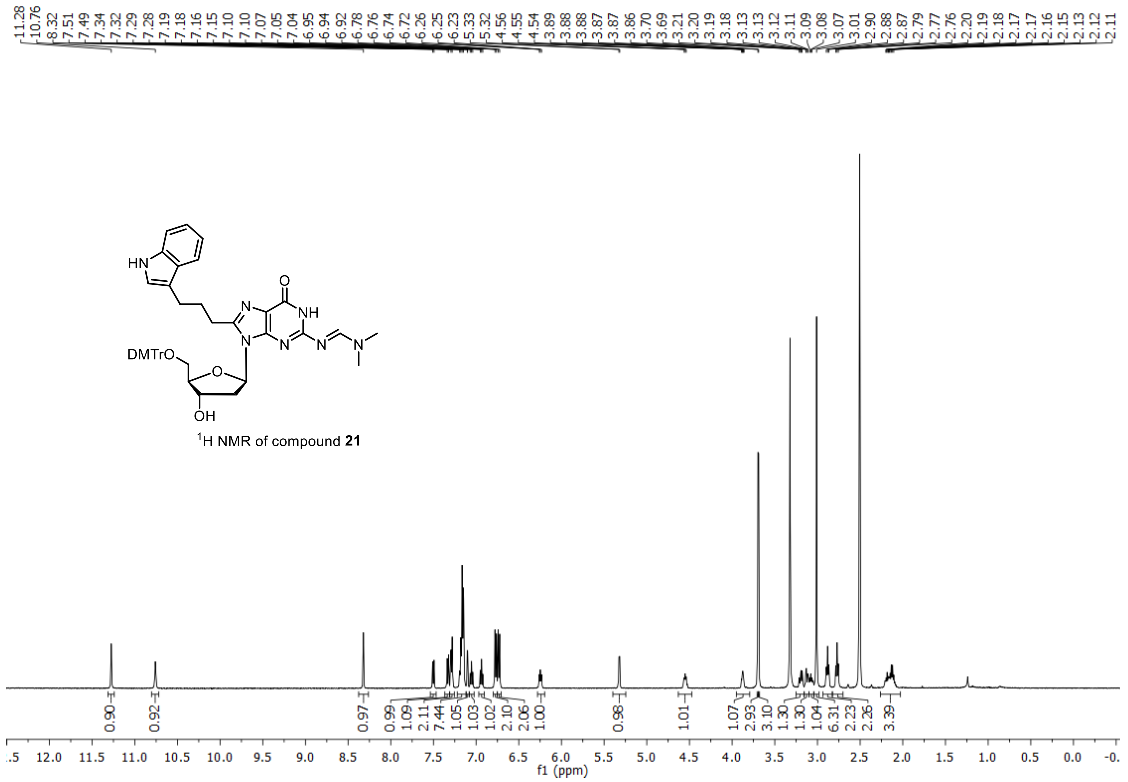

Figure S38 ^{1}H NMR spectrum of compound **21** (DMSO-*d6*)

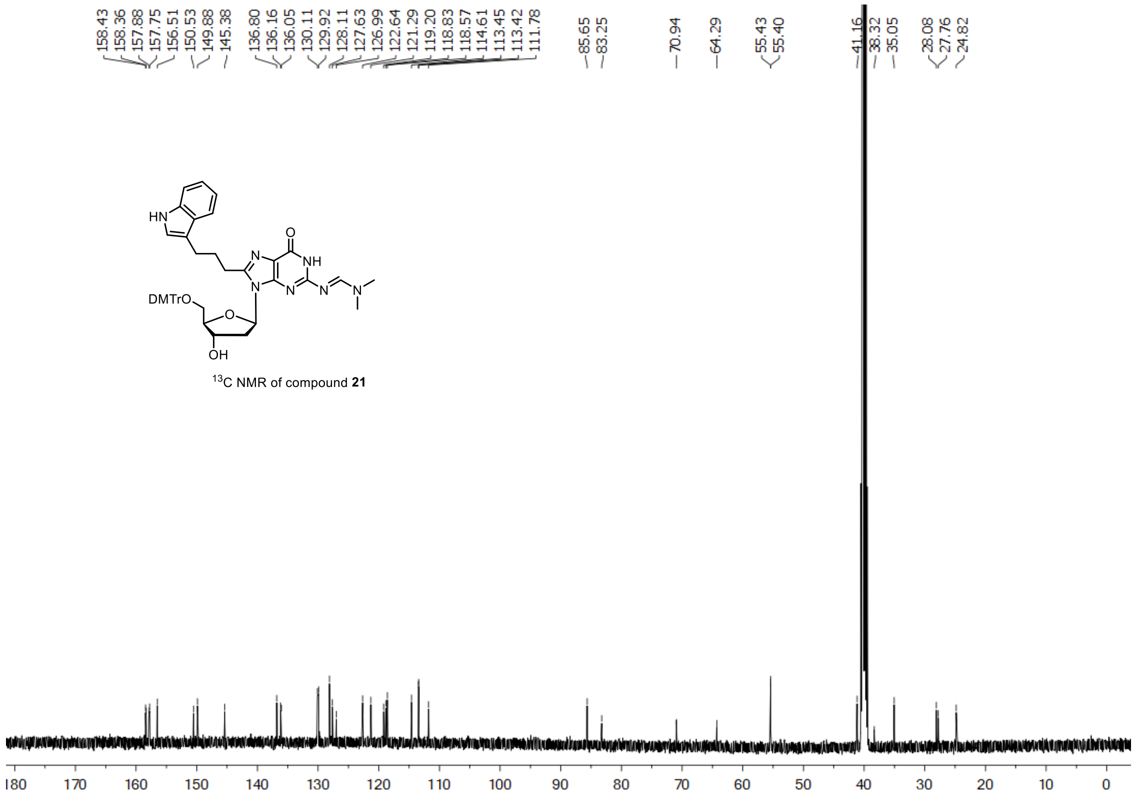

Figure S39 ^{13}C NMR spectrum of compound **21** (DMSO-*d6*)

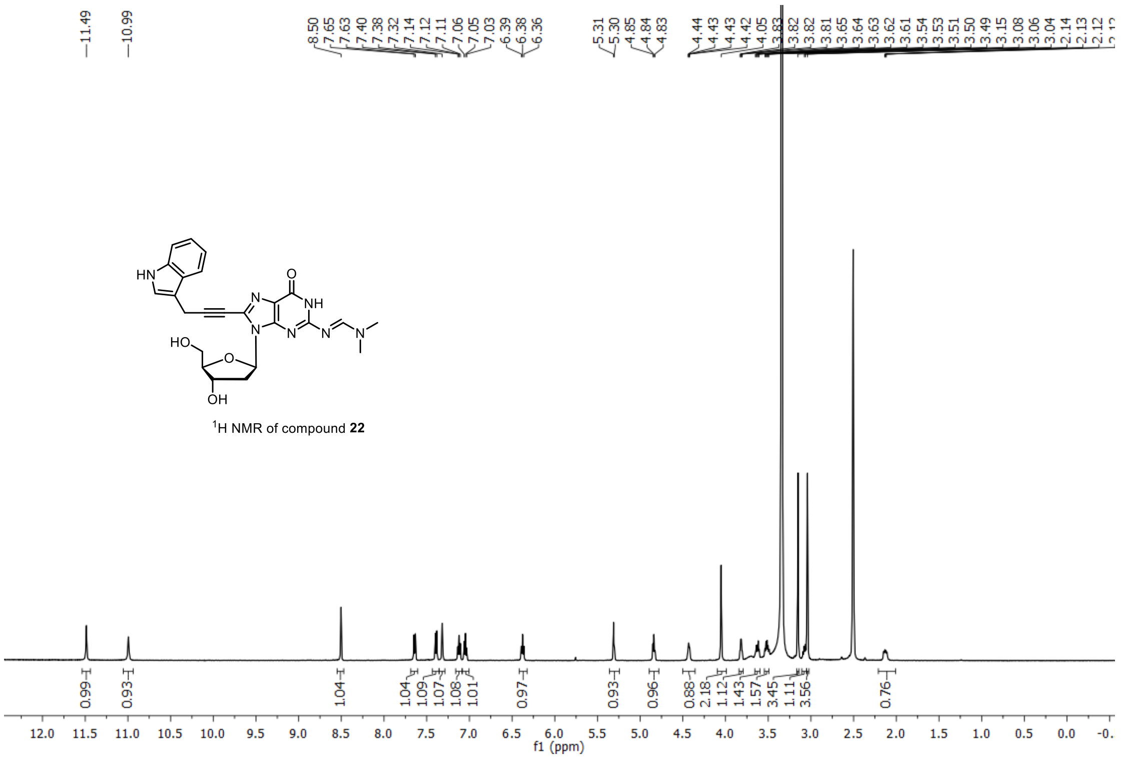

Figure S40 ^{1}H NMR spectrum of compound **22** (DMSO-*d6*)

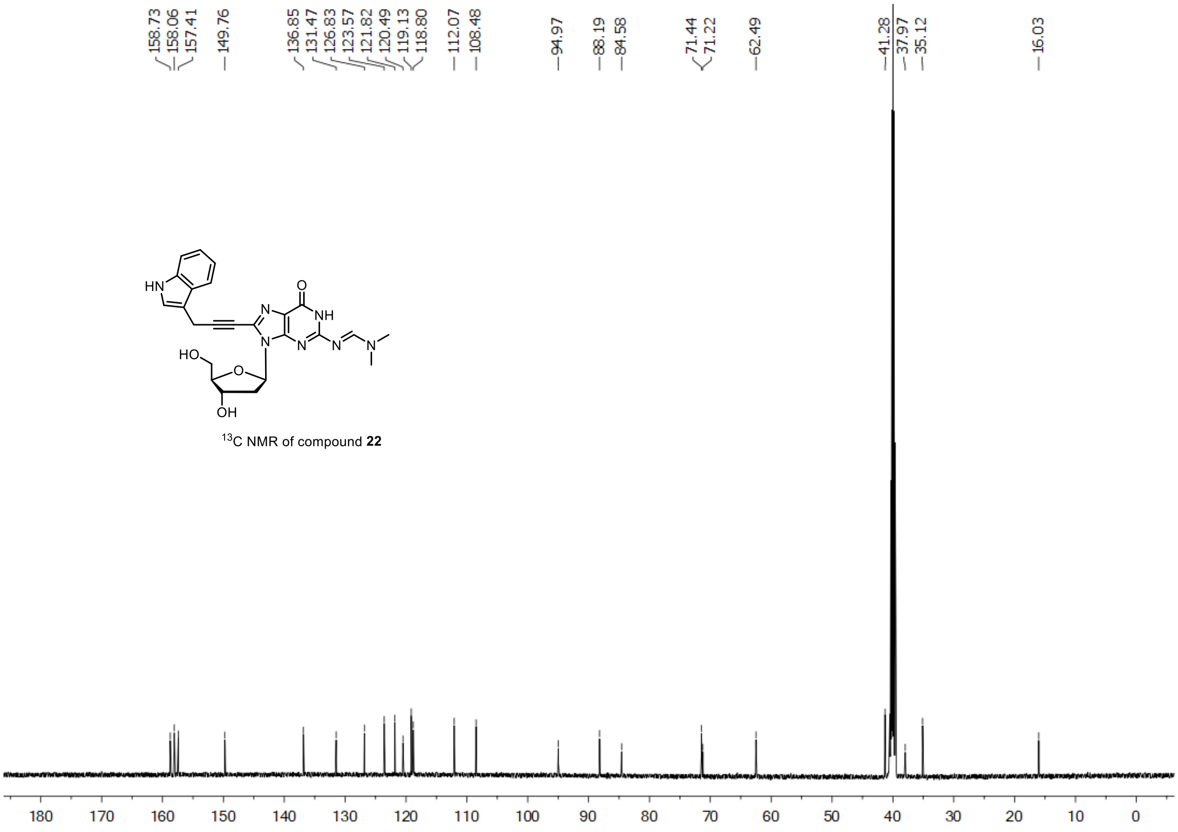

Figure S41 ^{13}C NMR spectrum of compound **22** (DMSO-*d6*)

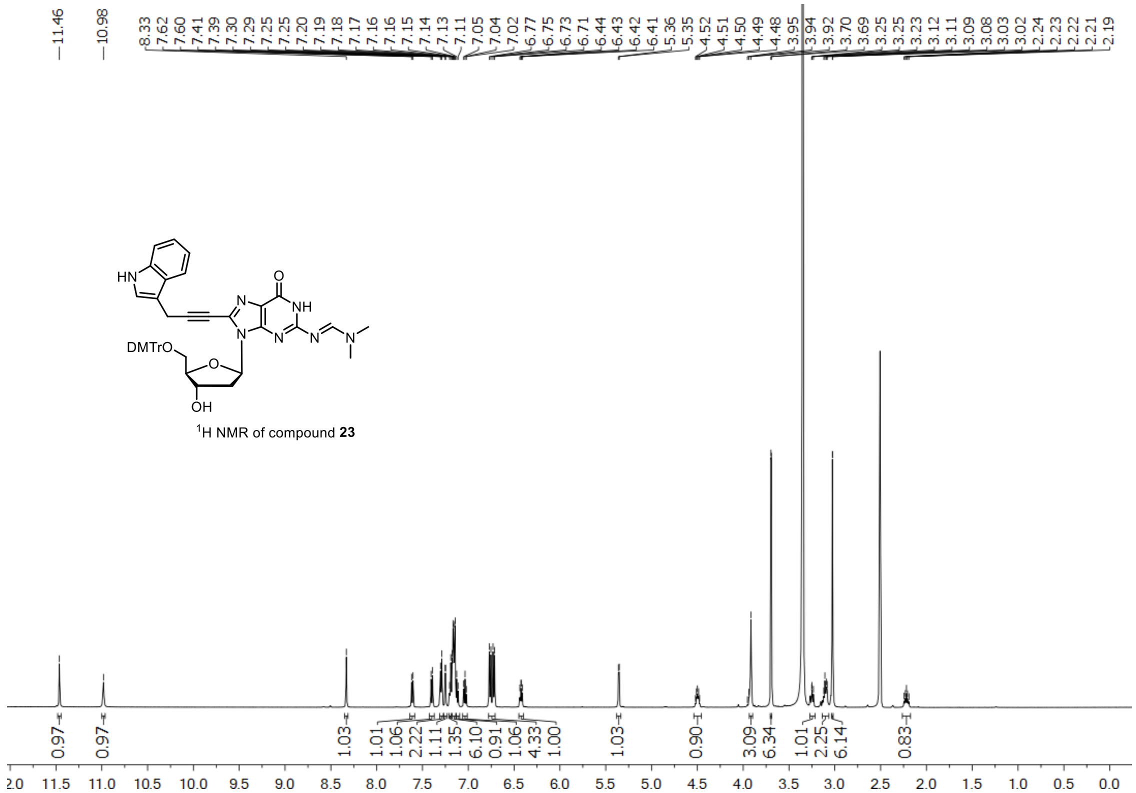

Figure S42 ^{1}H NMR spectrum of compound **23** (DMSO-*d6*)

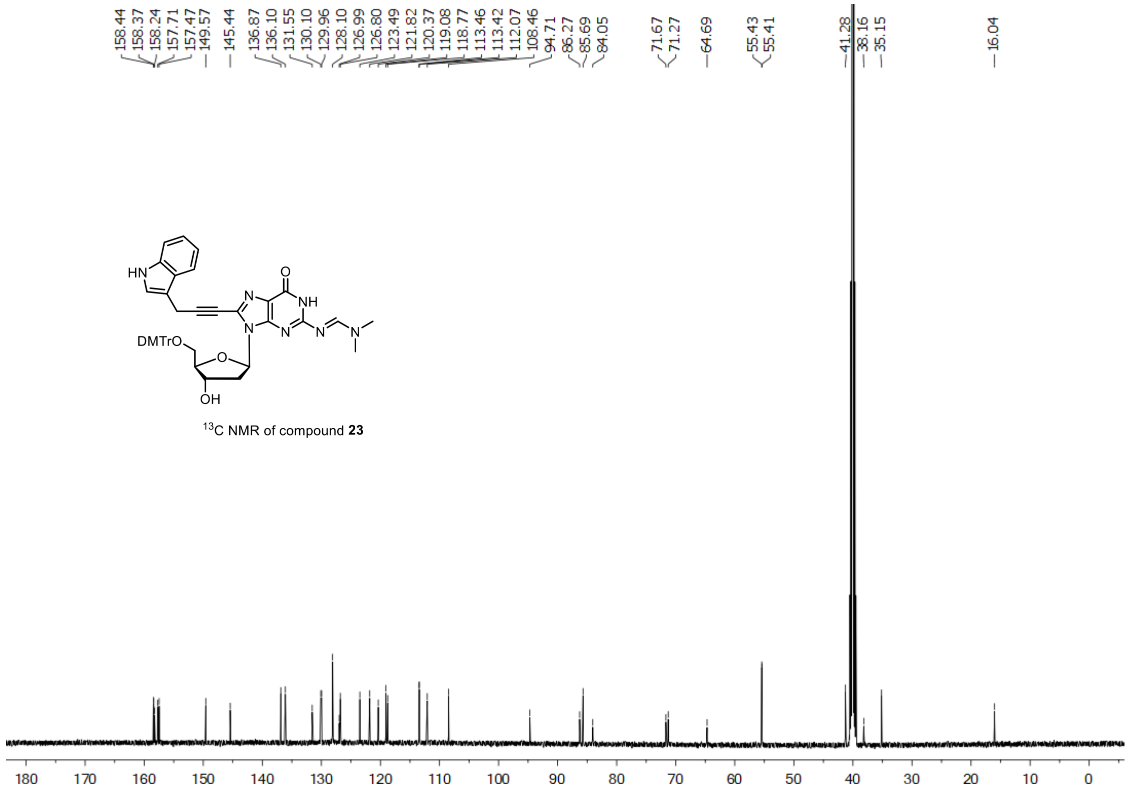

Figure S43 ^{13}C NMR spectrum of compound **23** (DMSO-*d6*)

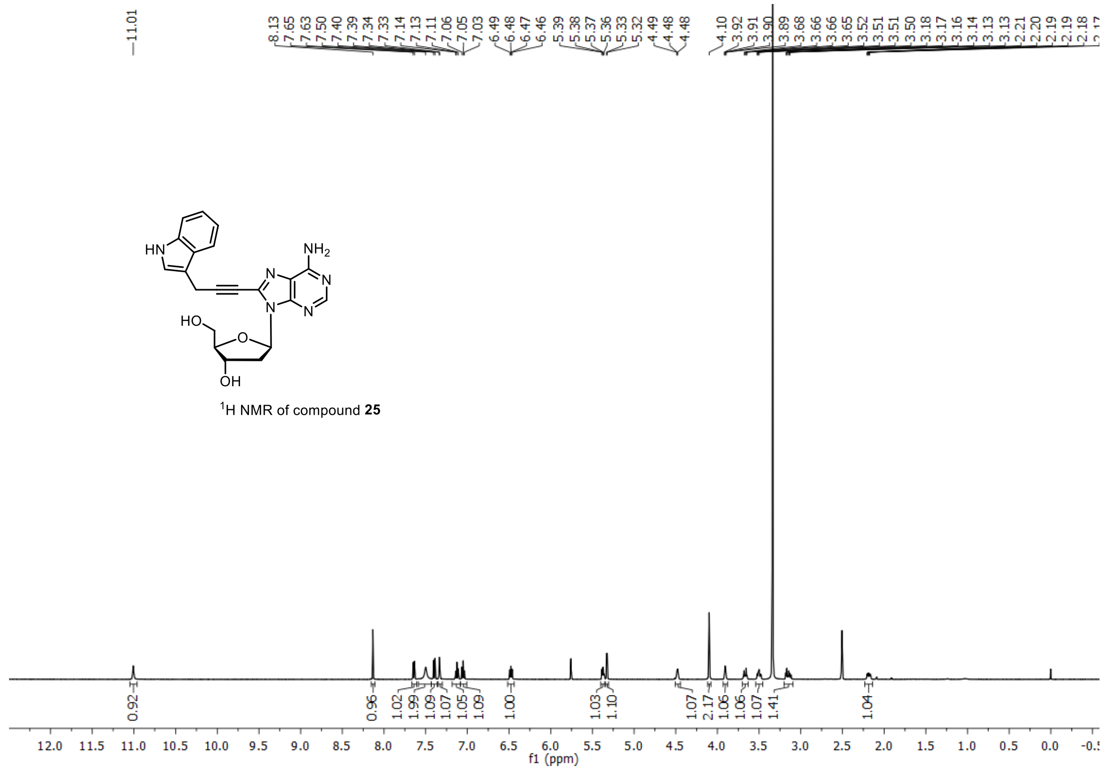

Figure S44 ^{1}H NMR spectrum of compound **25** (DMSO-*d6*)

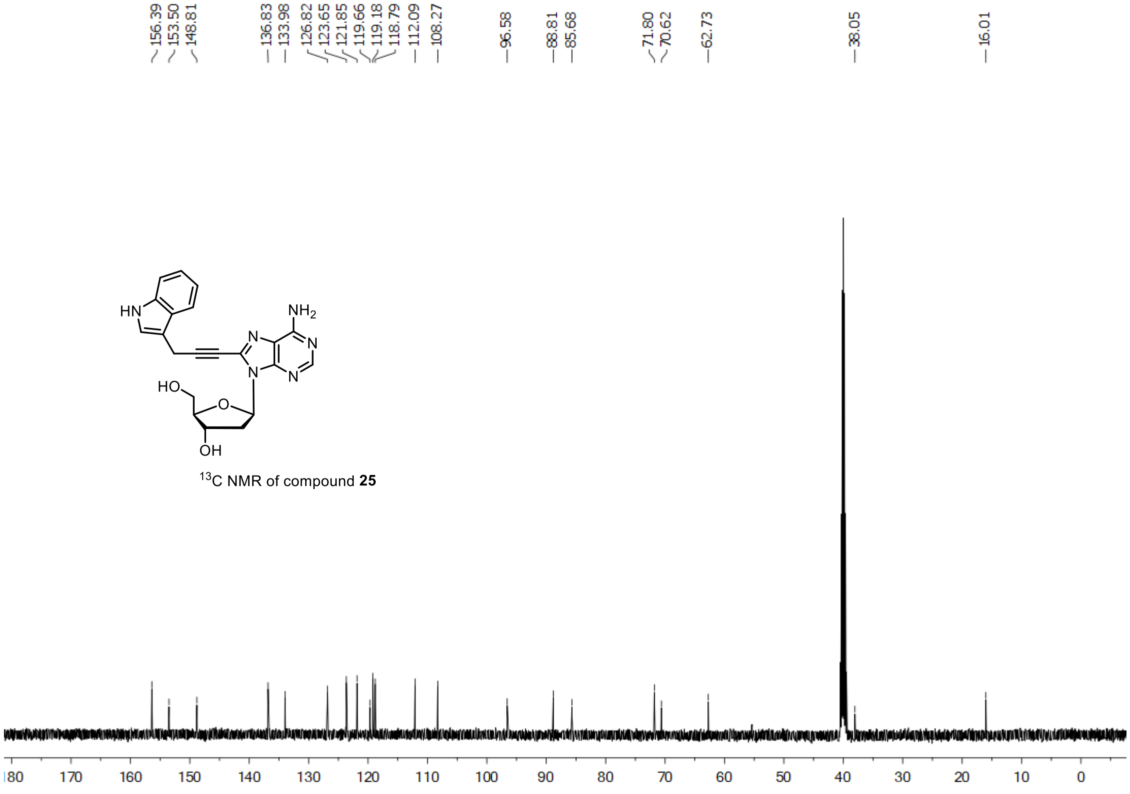

Figure S45 ^{13}C NMR spectrum of compound **25** (DMSO-*d6*)

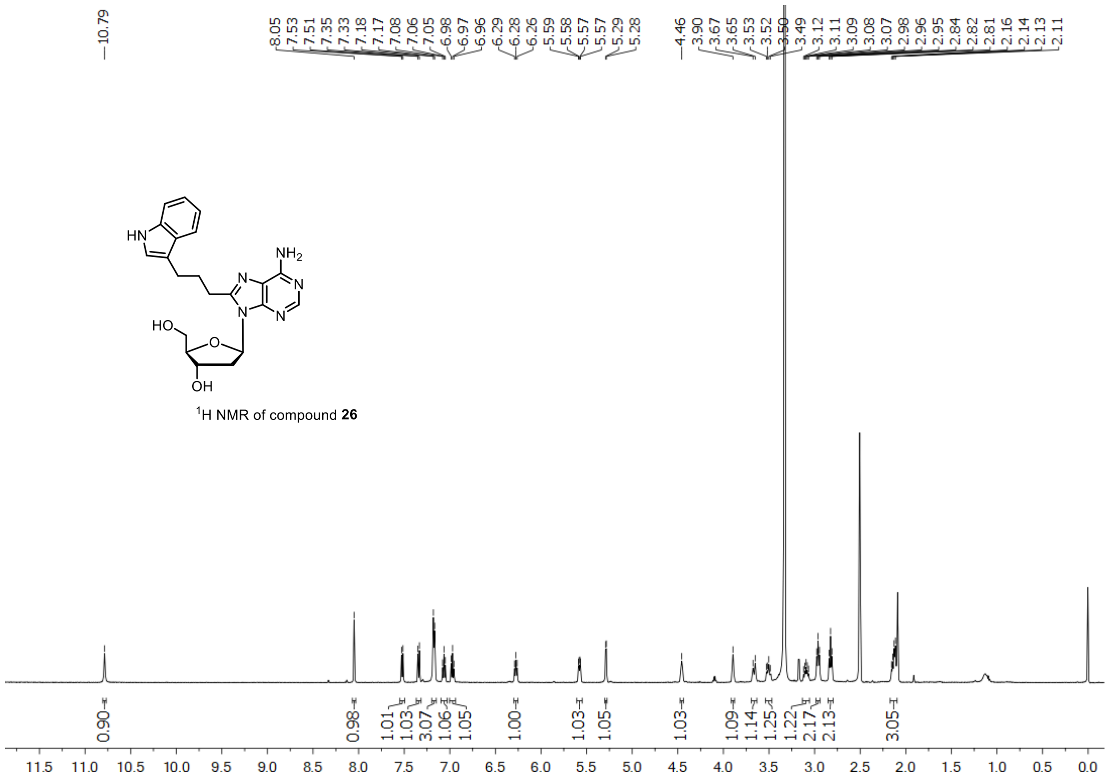

Figure S46 ^{1}H NMR spectrum of compound **26** (DMSO-*d6*)

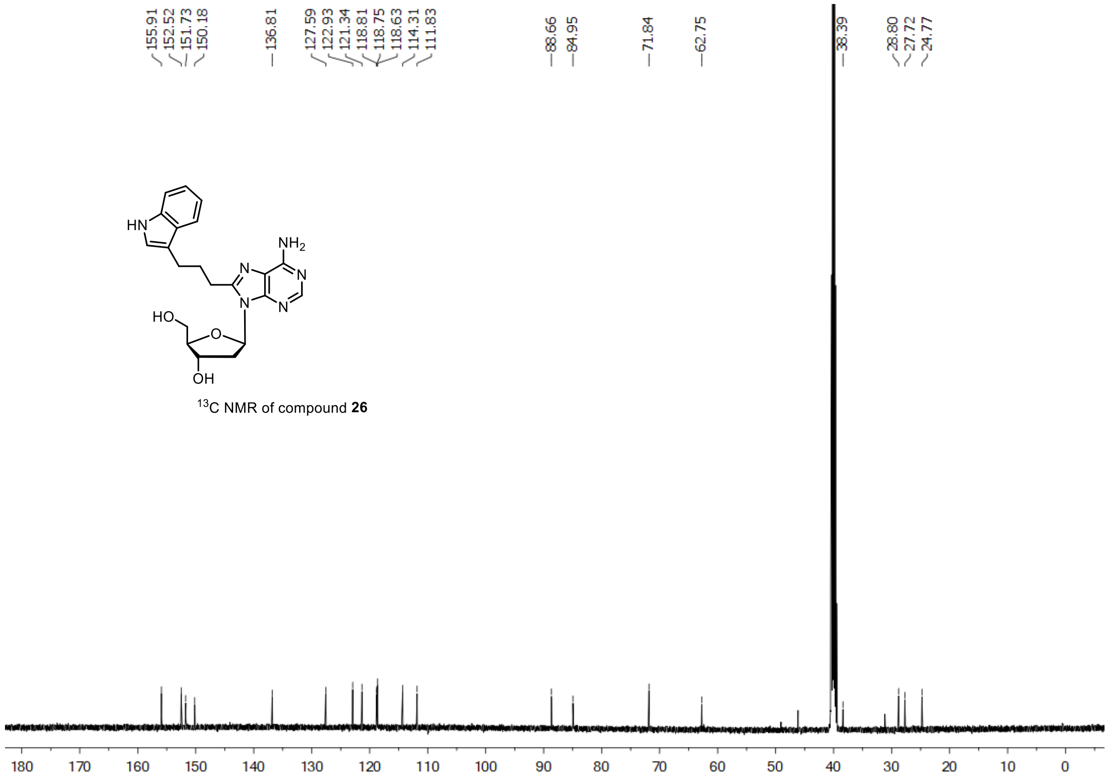

Figure S47 ^{13}C NMR spectrum of compound **26** (DMSO-*d6*)

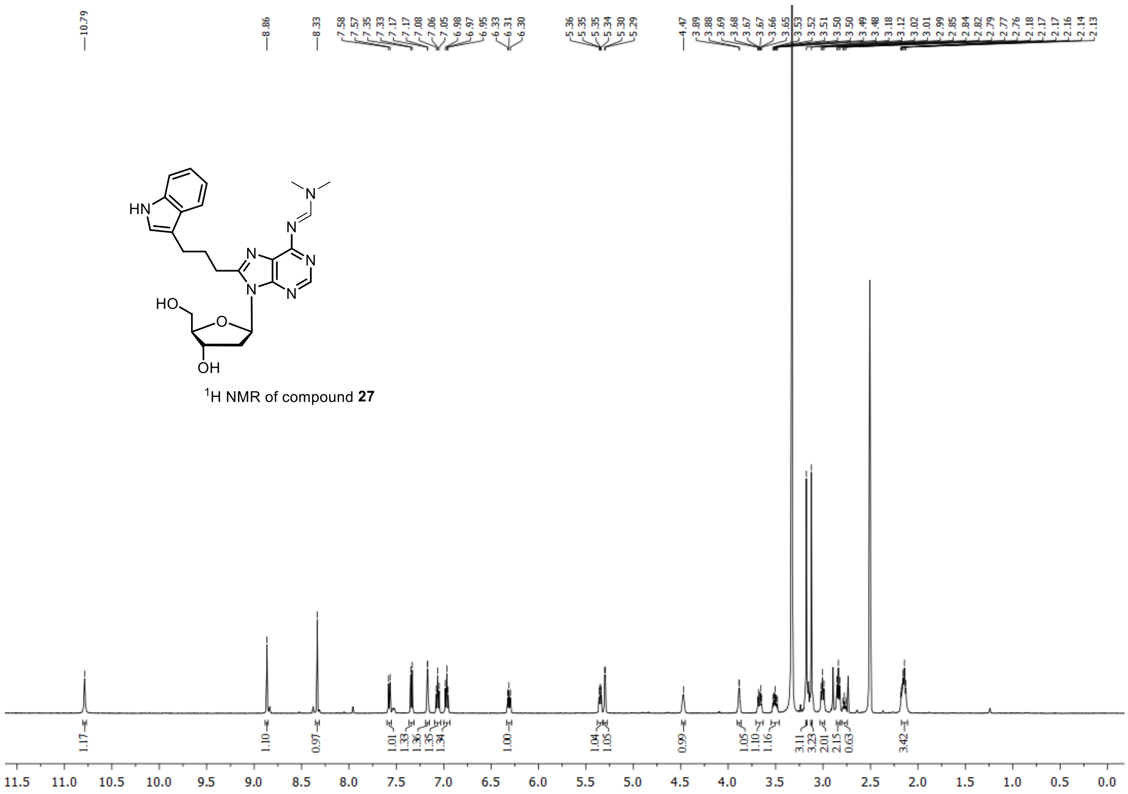

Figure S48 ^{1}H NMR spectrum of compound **27** (DMSO-*d6*)

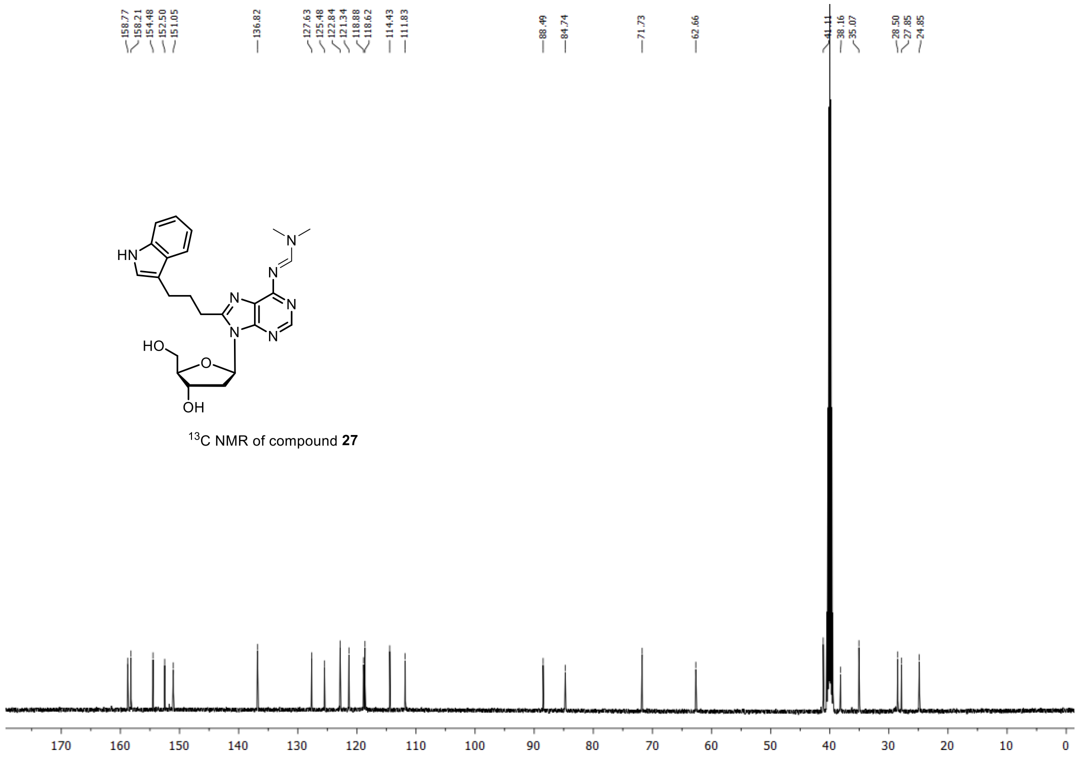

Figure S49 ^{13}C NMR spectrum of compound **27** (DMSO-*d6*)

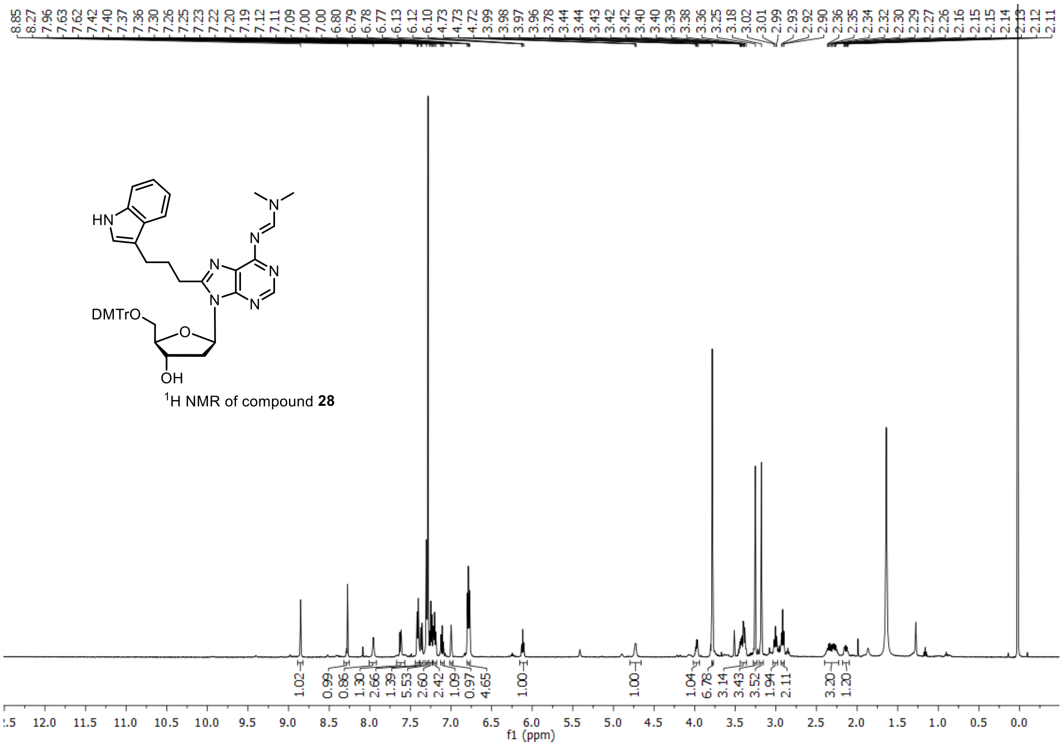

Figure S50 ^{1}H NMR spectrum of compound **28** ($CDCl_3$)

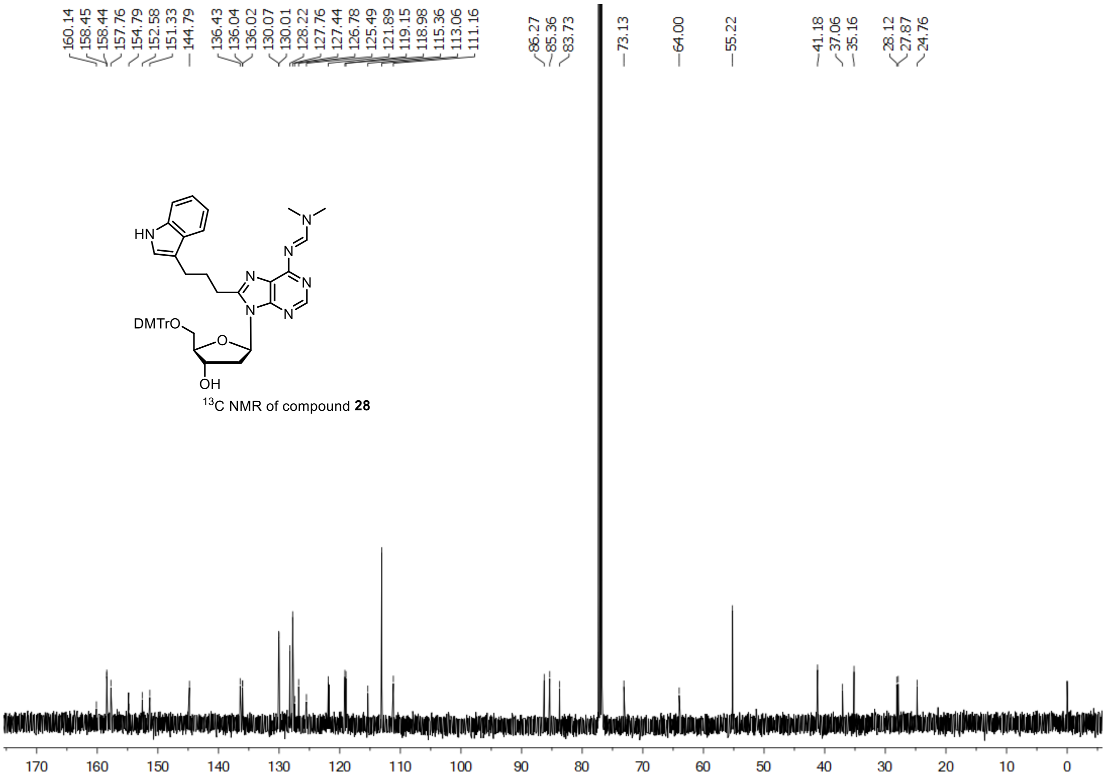

Figure S51 ^{13}C NMR spectrum of compound **28** ($CDCl_3$)

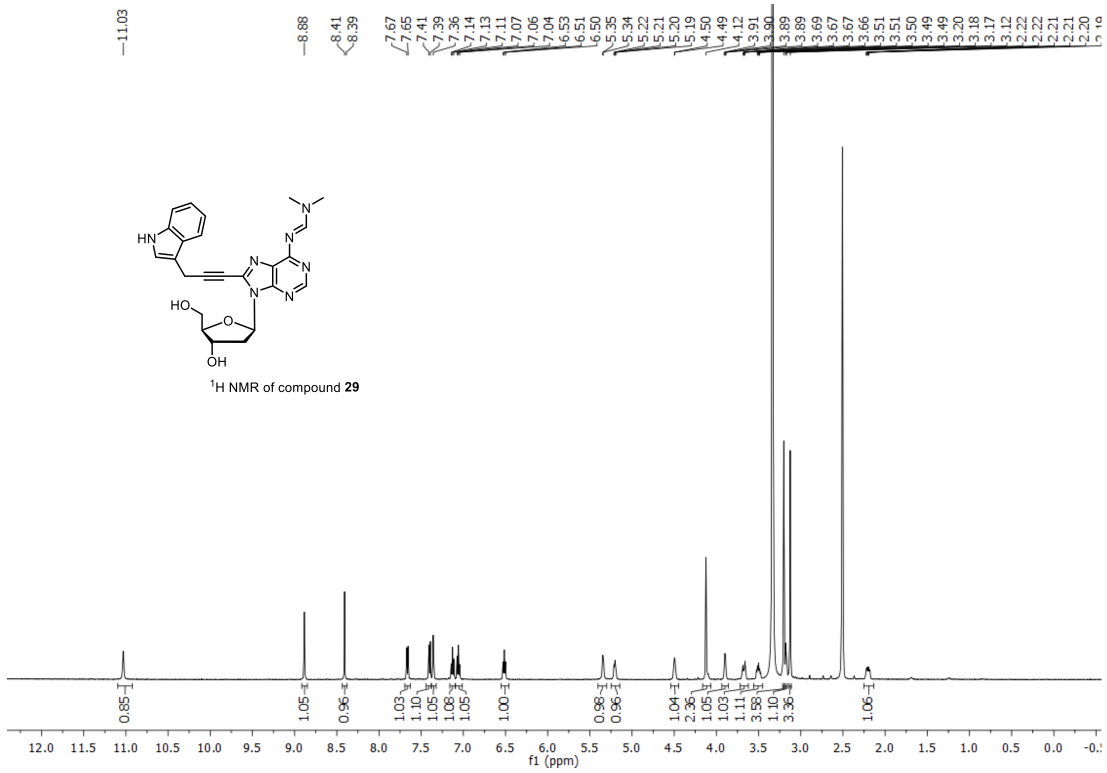

Figure S52 ^{1}H NMR spectrum of compound **29** (DMSO-*d6*)

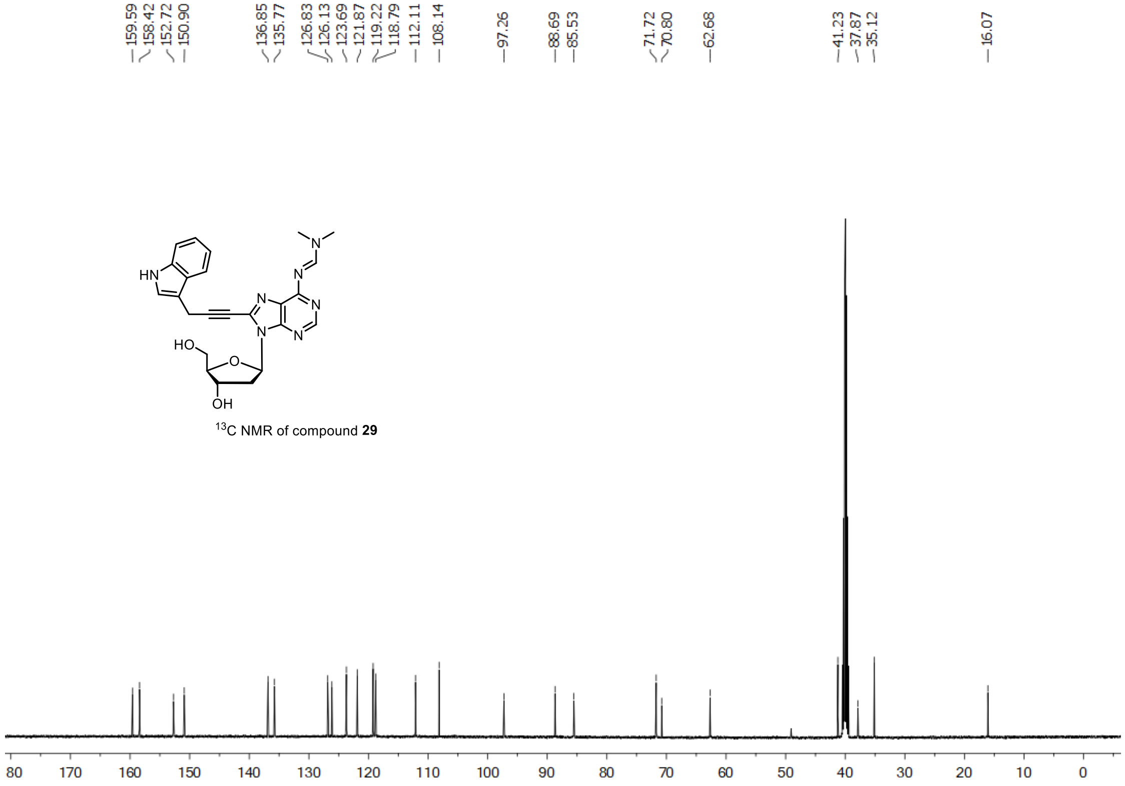

Figure S53 ^{13}C NMR spectrum of compound **29** (DMSO-*d6*)

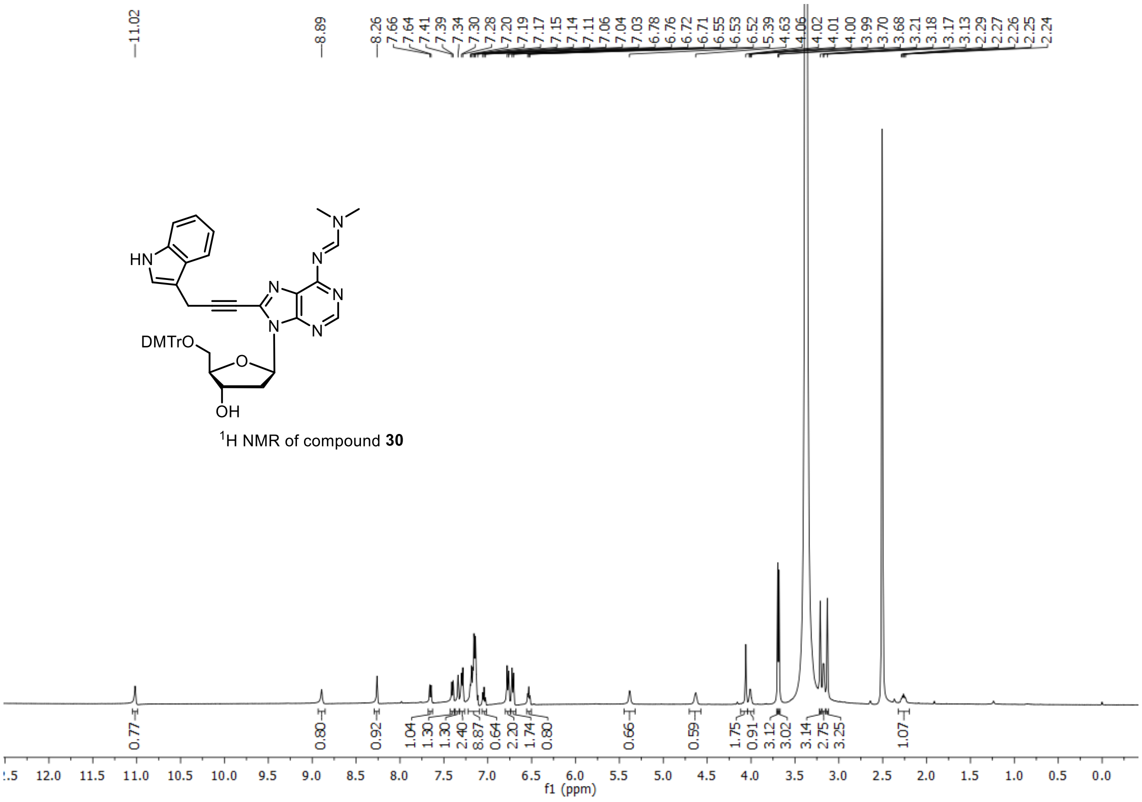

Figure S54 ^{1}H NMR spectrum of compound **30** (DMSO-*d6*)

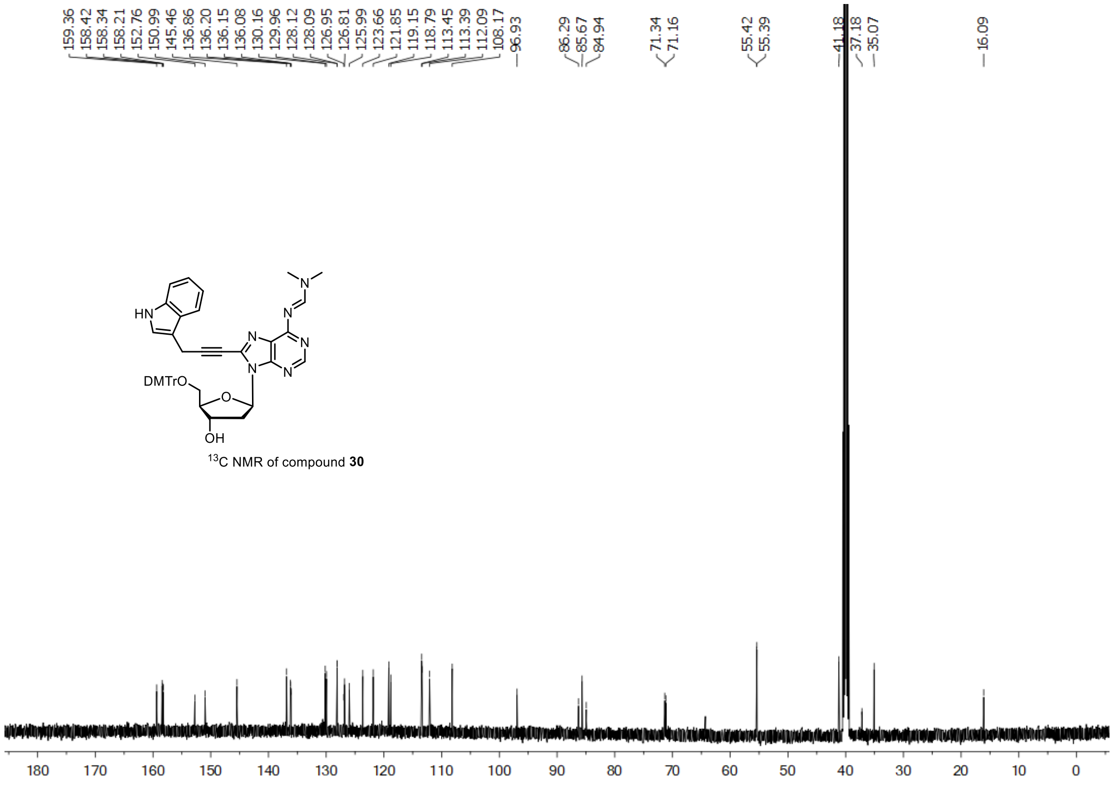

Figure S55 ^{13}C NMR spectrum of compound **30** (DMSO-*d6*)

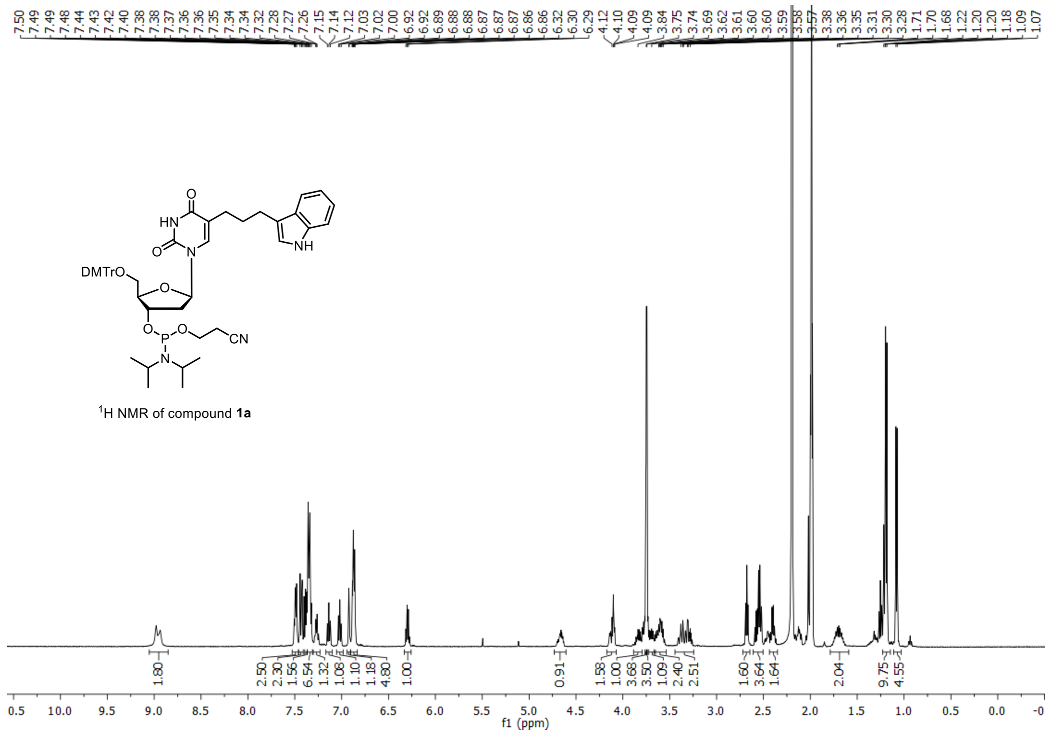

Figure S56 ${}^{1}H$ NMR spectrum of compound **1a** (CD_3CN)

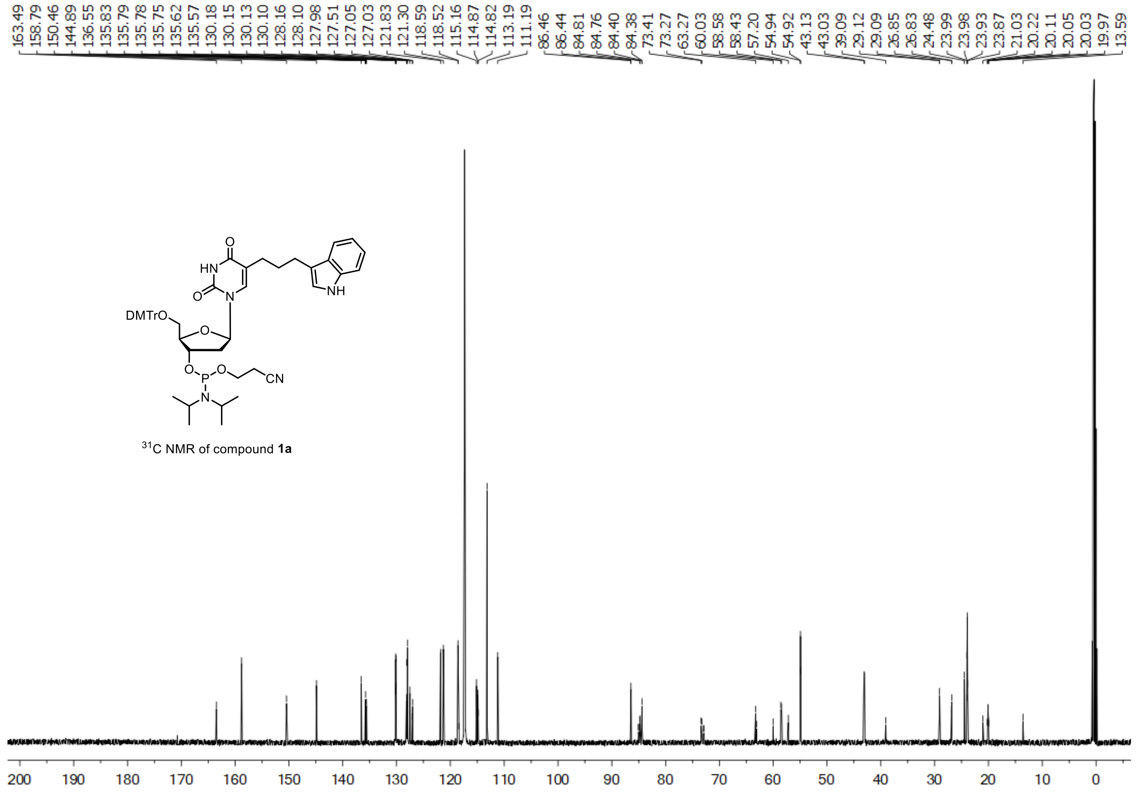

Figure S57 ${}^{13}C$ NMR spectrum of compound **1a** (CD_3CN)

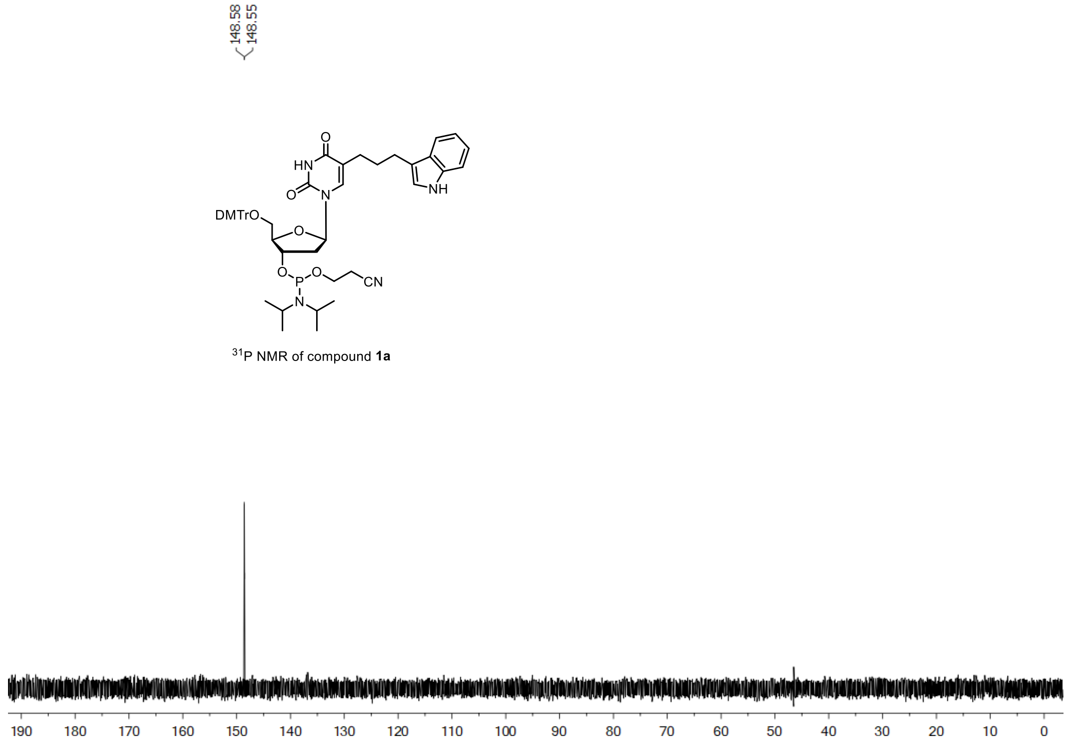

Figure S58 ^{31}P NMR spectrum of compound **1a** (CD_3CN)

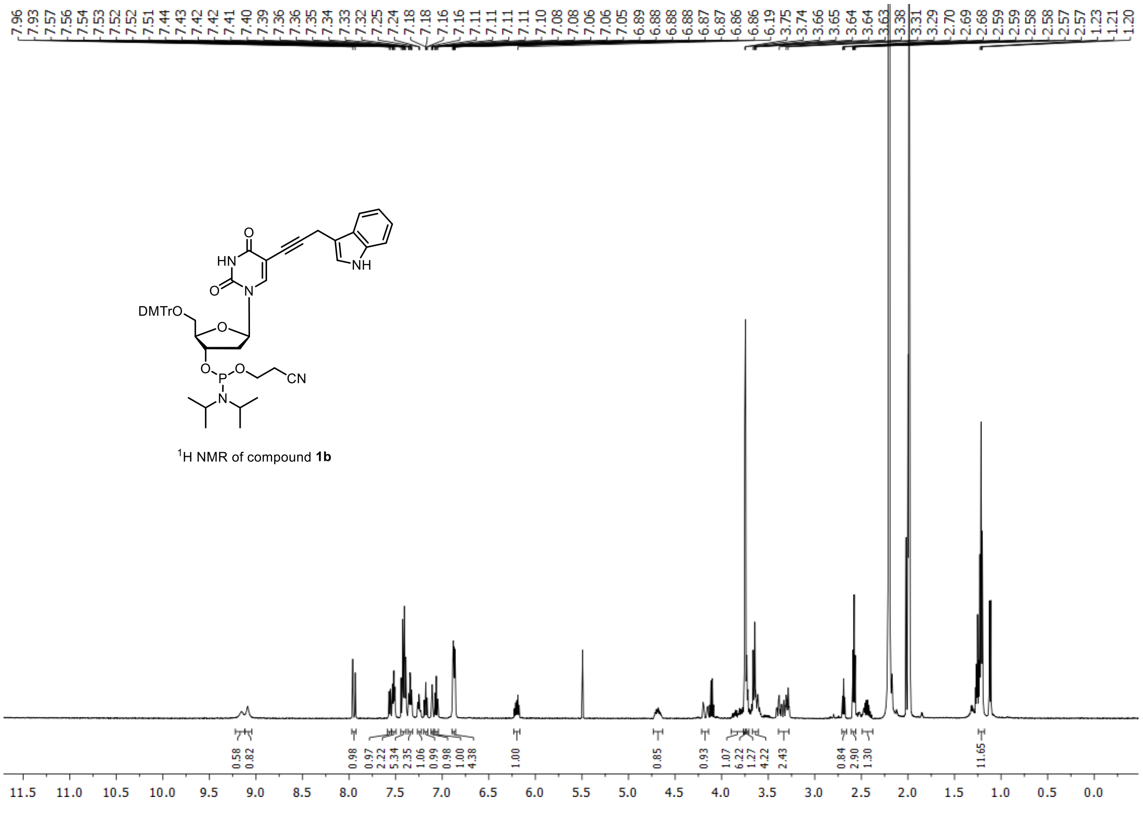

Figure S59 ^{1}H NMR spectrum of compound **1b** (CD_3CN)

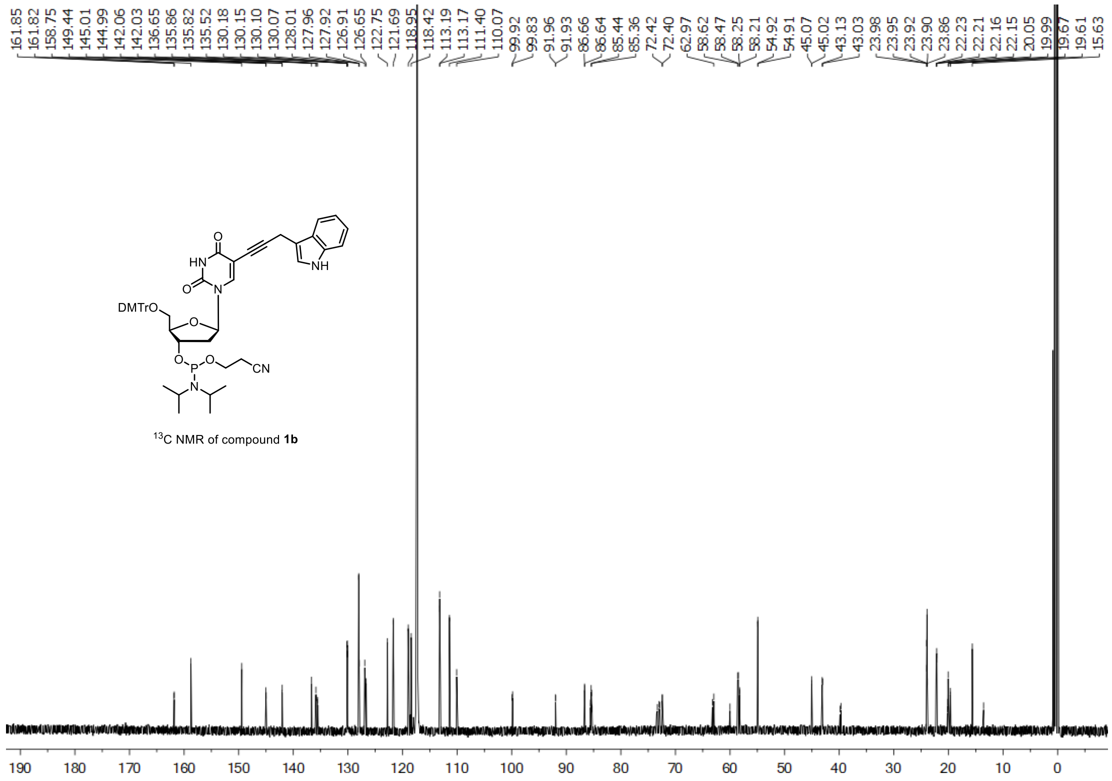

Figure S60 ^{13}C NMR spectrum of compound **1b** (CD_3CN)

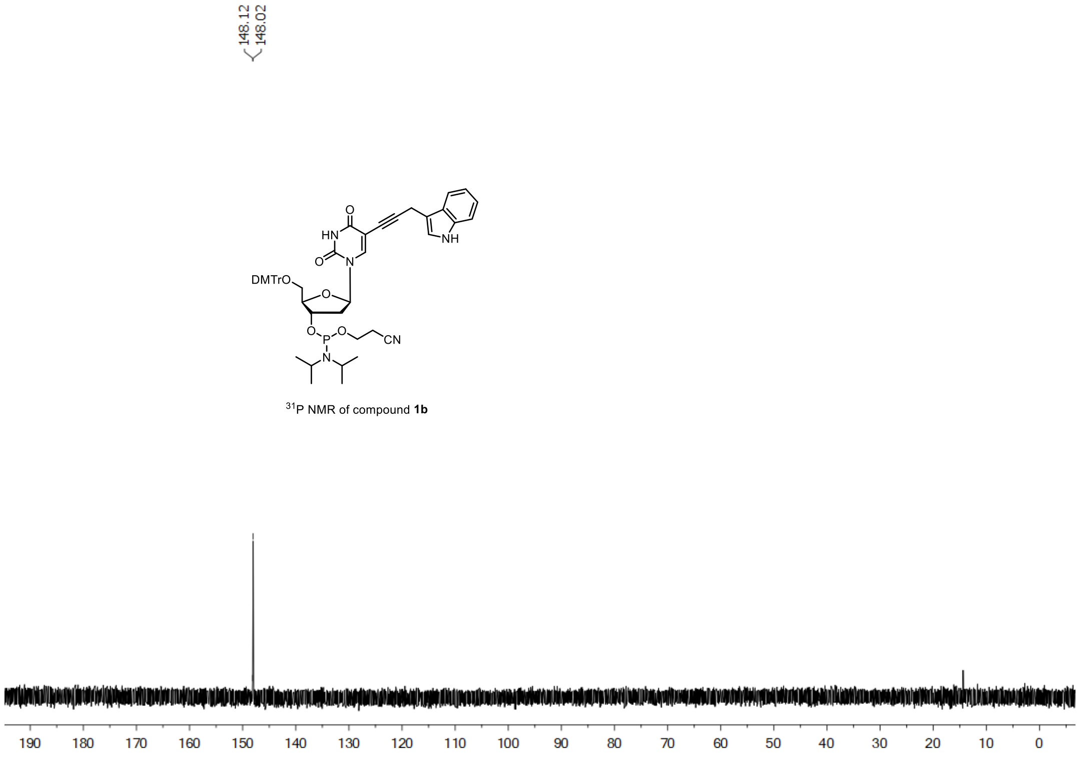

Figure S61 ^{31}P NMR spectrum of compound **1b** (CD_3CN)

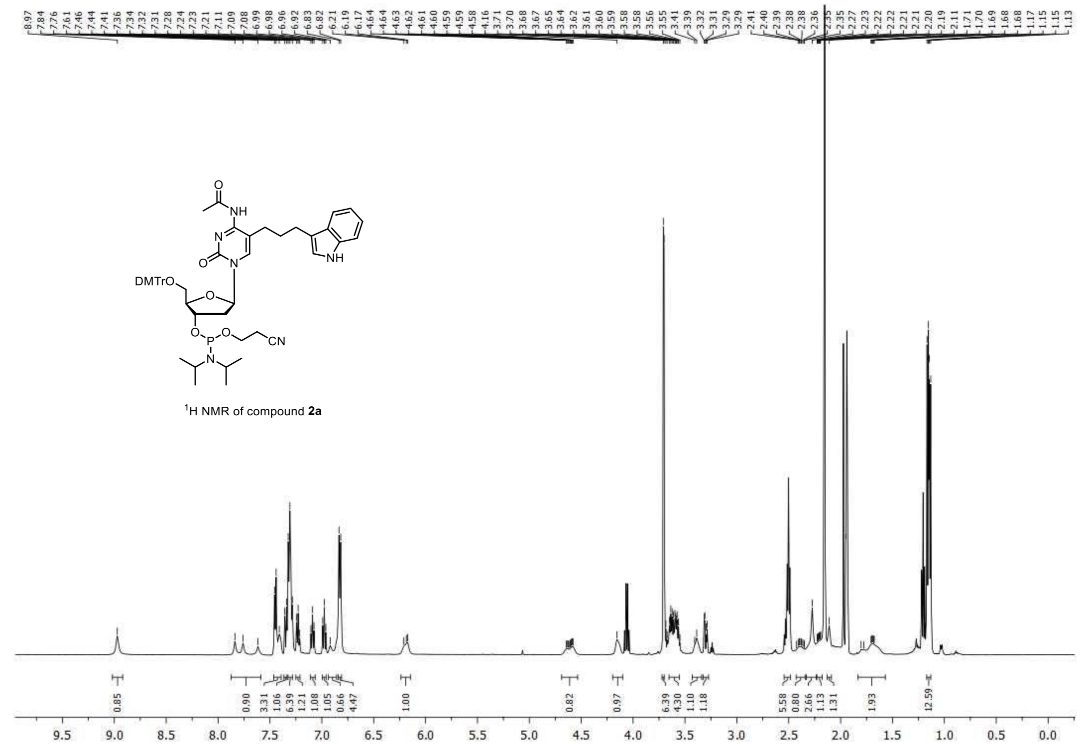

Figure S62 ^{1}H NMR spectrum of compound **2a** (CD_3CN)

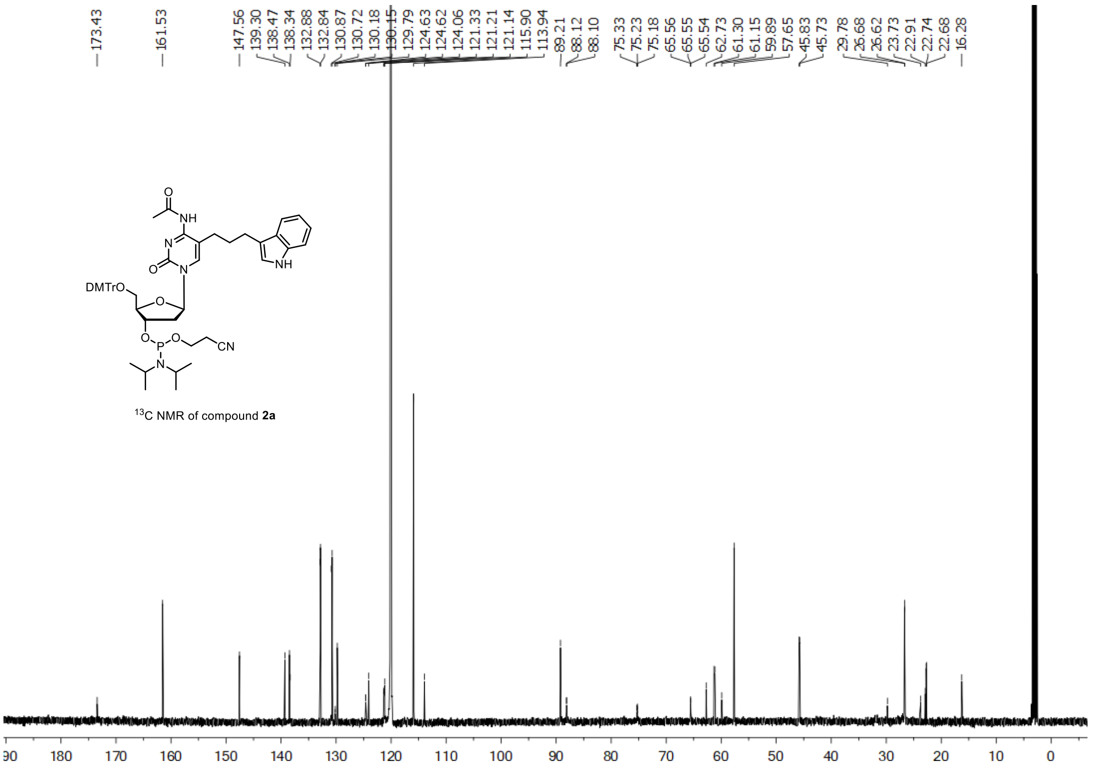

Figure S63 ^{13}C NMR spectrum of compound **2a** (CD_3CN)

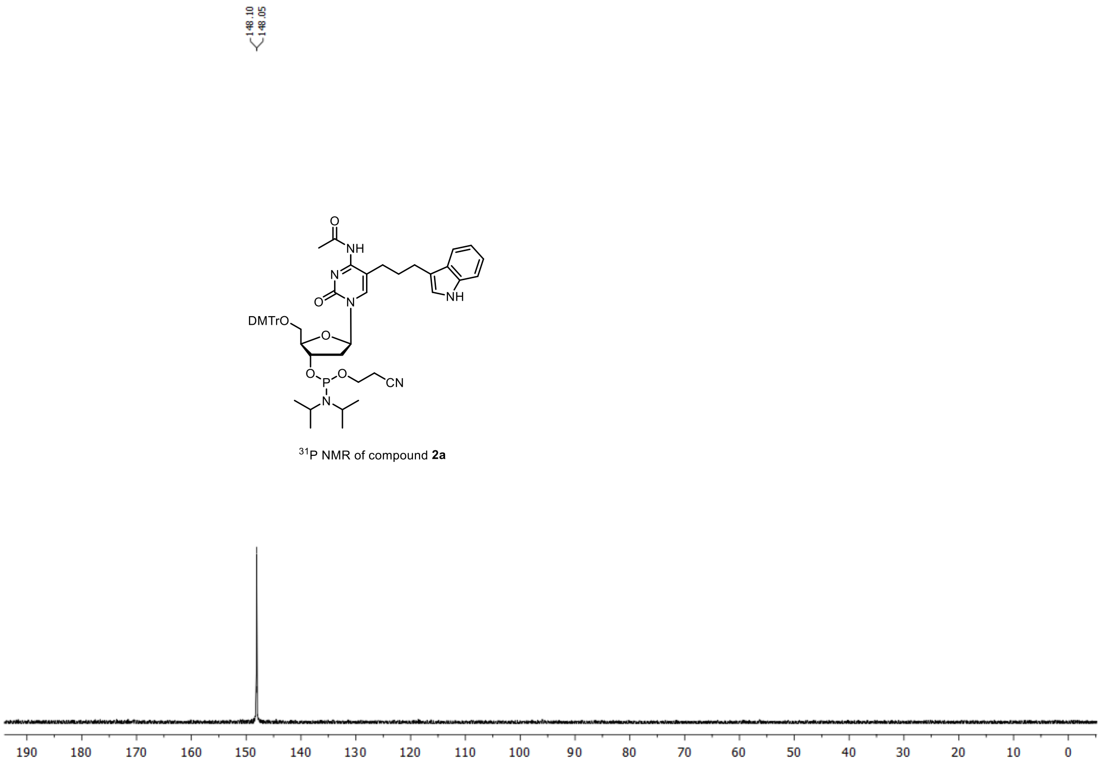

Figure S64 ^{31}P NMR spectrum of compound **2a** (CD_3CN)

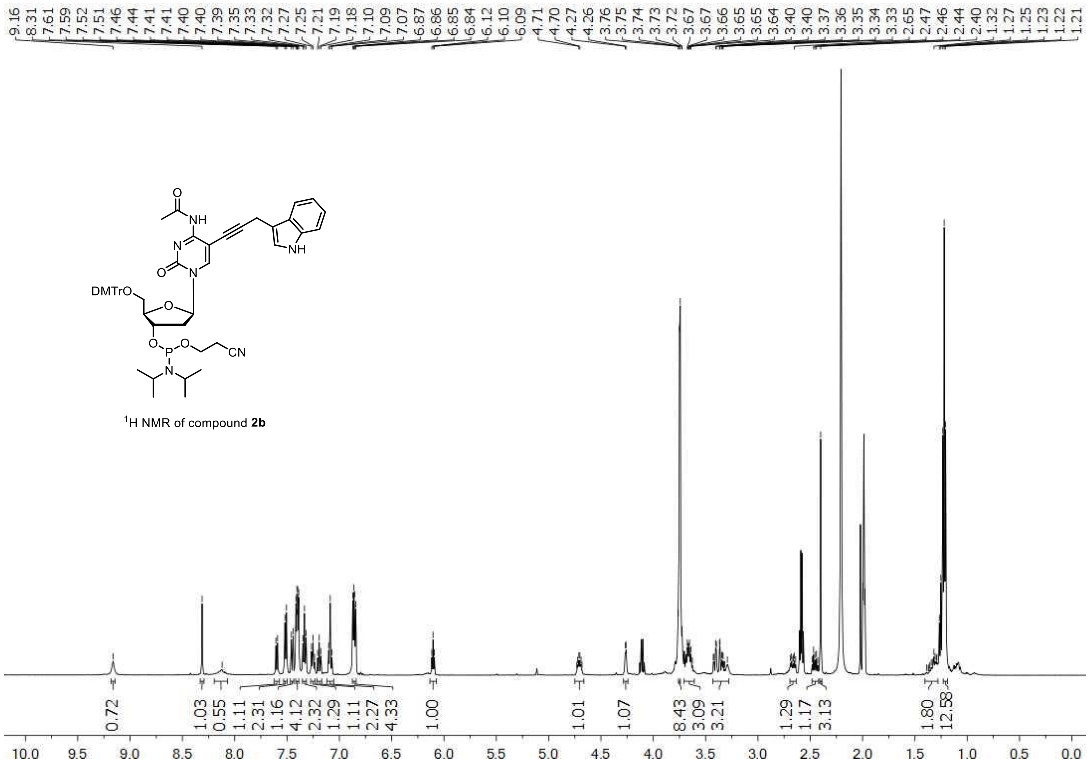

Figure S65 ^{1}H NMR spectrum of compound **2b** (CD_3CN)

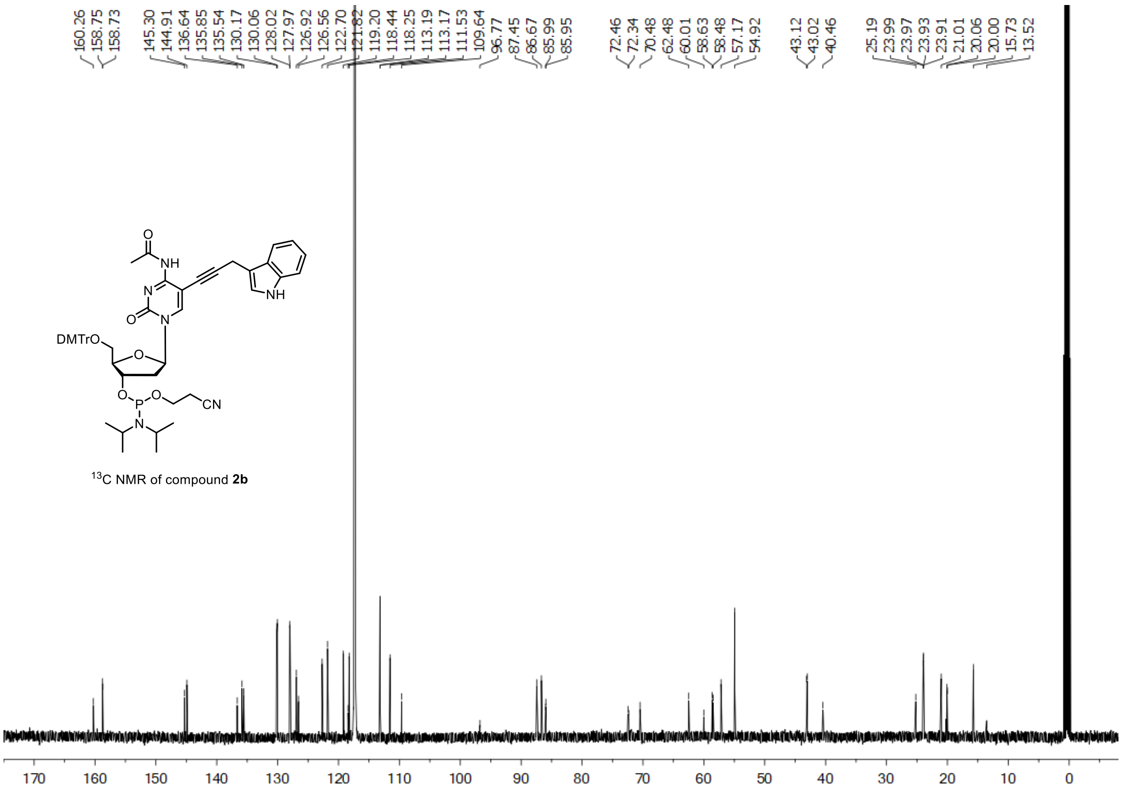

Figure S66 ^{13}C NMR spectrum of compound **2b** (CD_3CN)

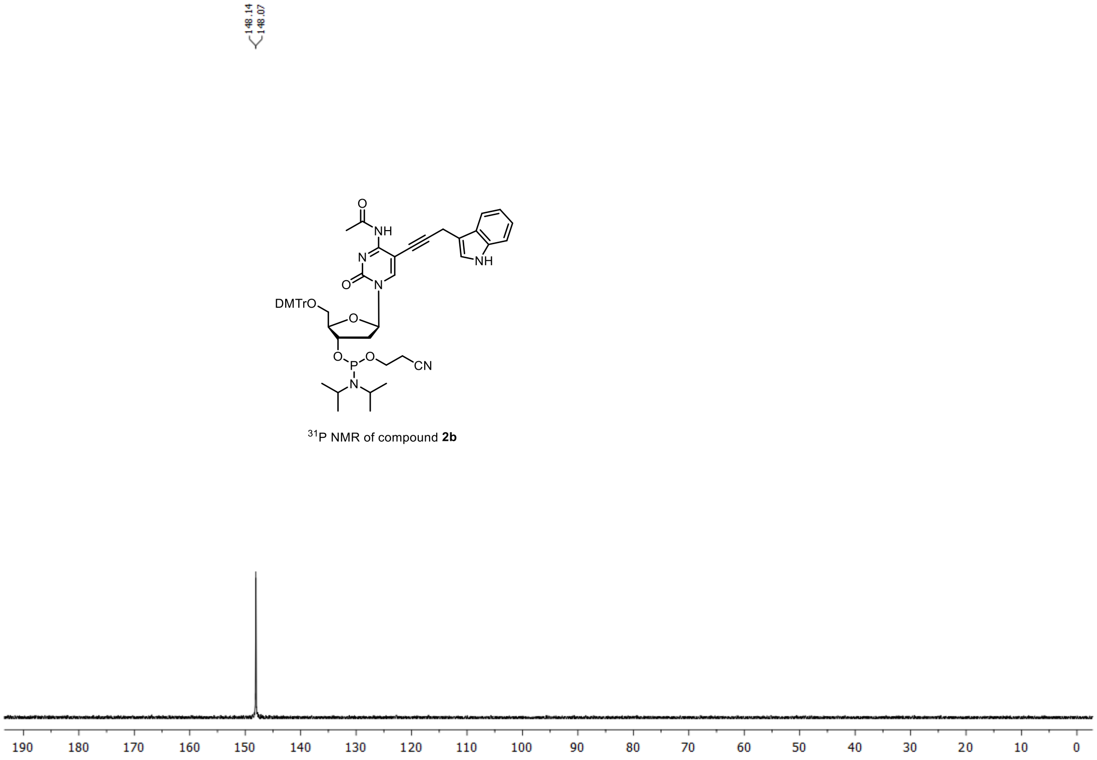

Figure S67 ^{31}P NMR spectrum of compound **2b** (CD_3CN)

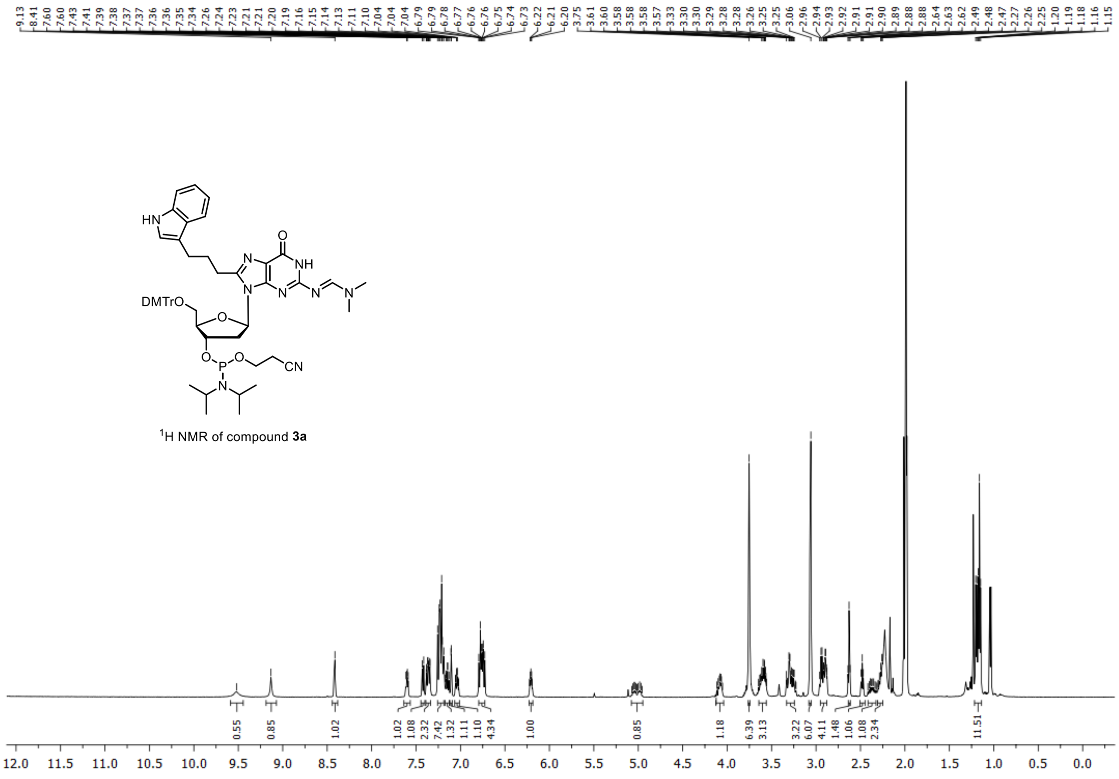

Figure S68 ^{1}H NMR spectrum of compound **3a** (CD_3CN)

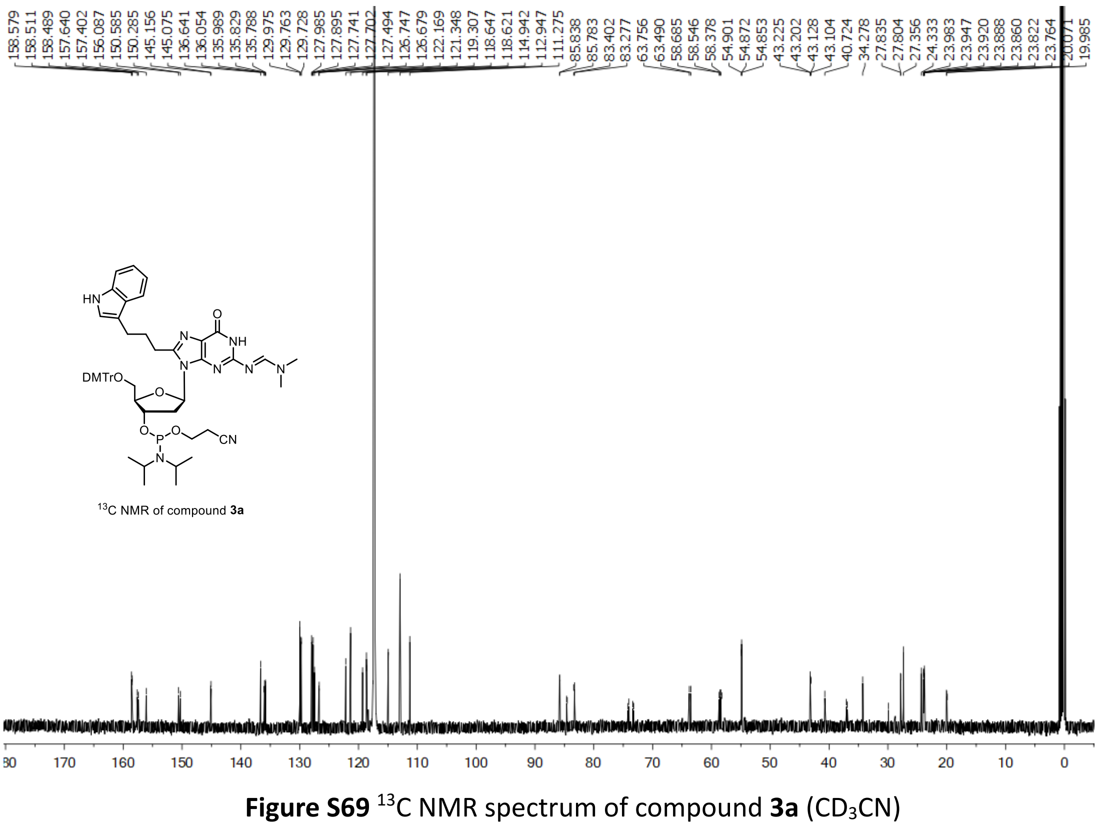

Figure S69 ^{13}C NMR spectrum of compound **3a** (CD_3CN)

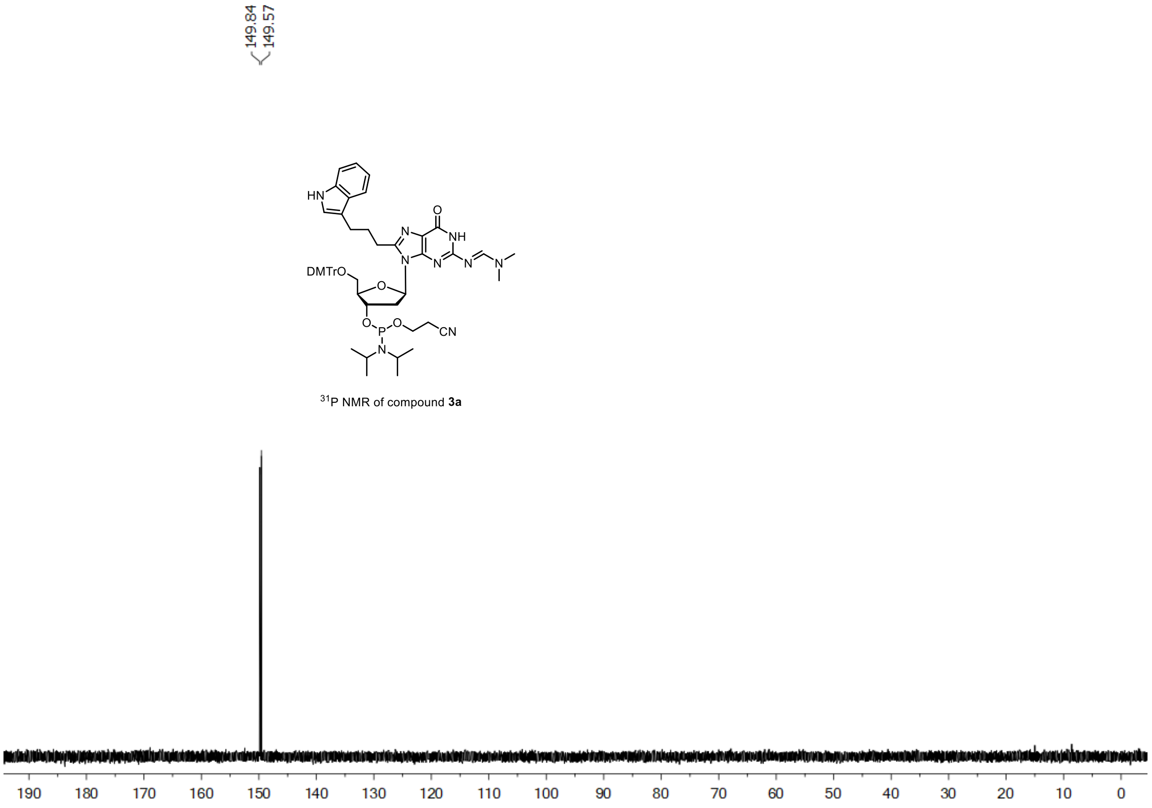

Figure S70 ^{31}P NMR spectrum of compound **3a** (CD_3CN)

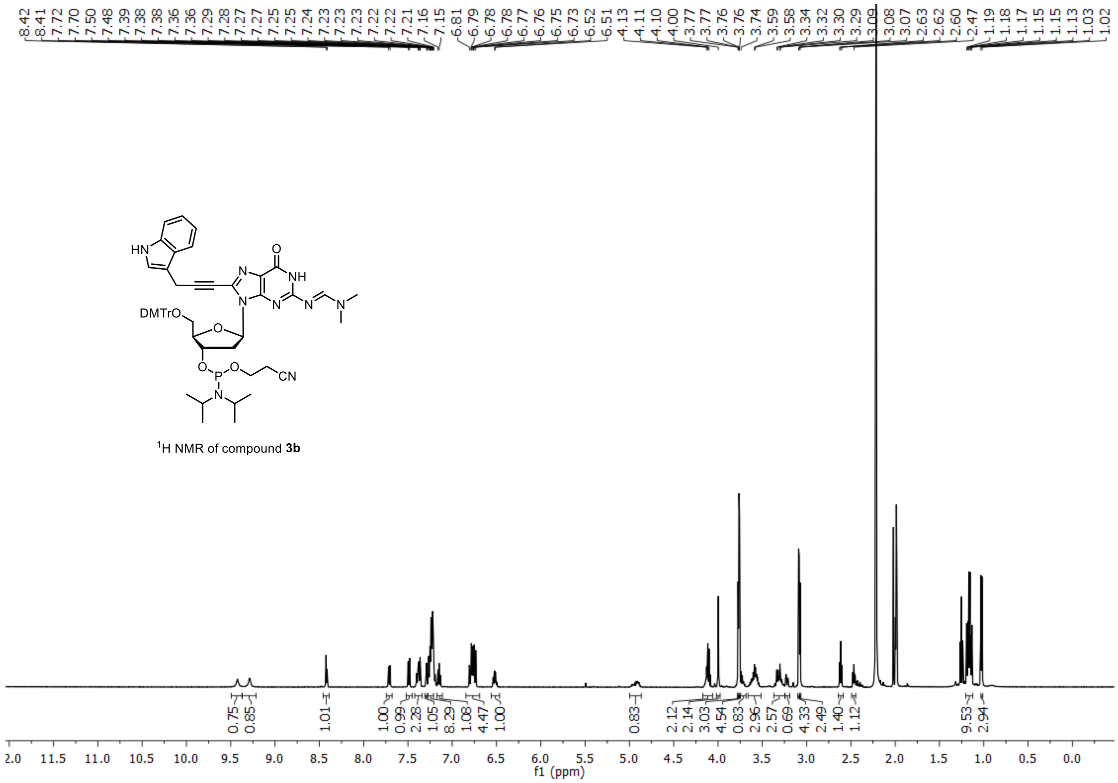

Figure S71 ^{1}H NMR spectrum of compound **3b** (CD_3CN)

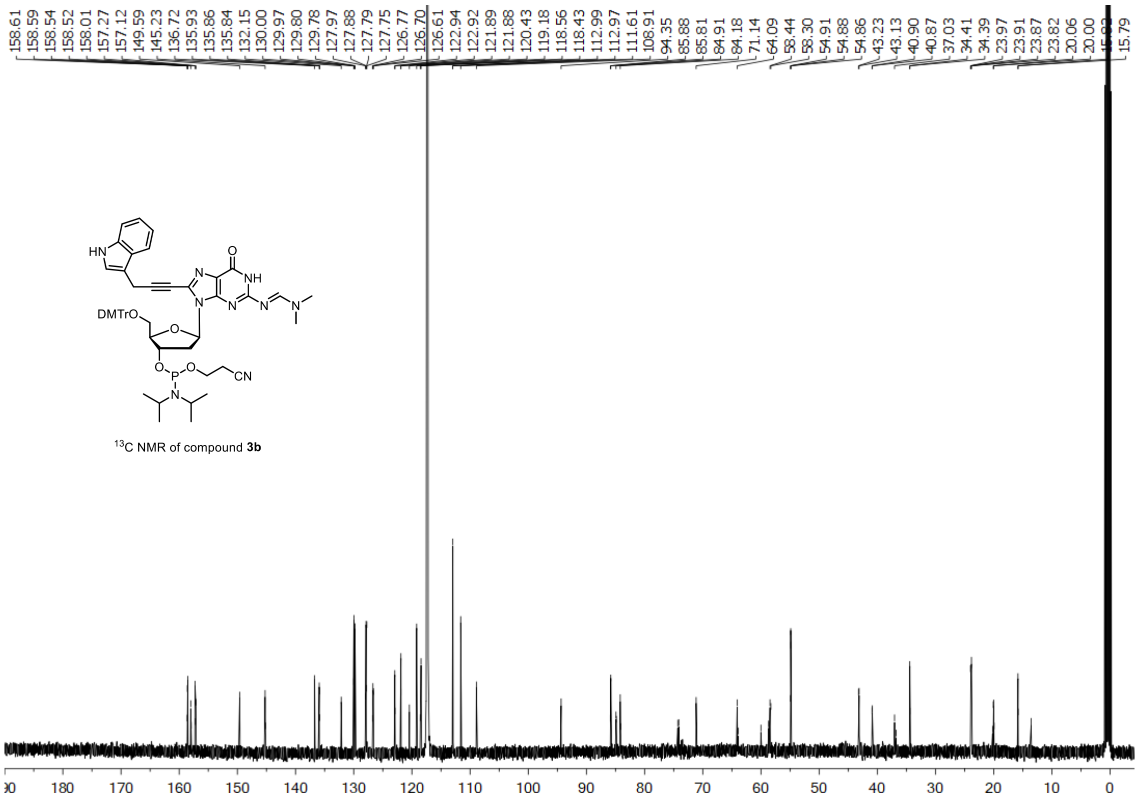

Figure S72 ^{13}C NMR spectrum of compound **3b** (CD_3CN)

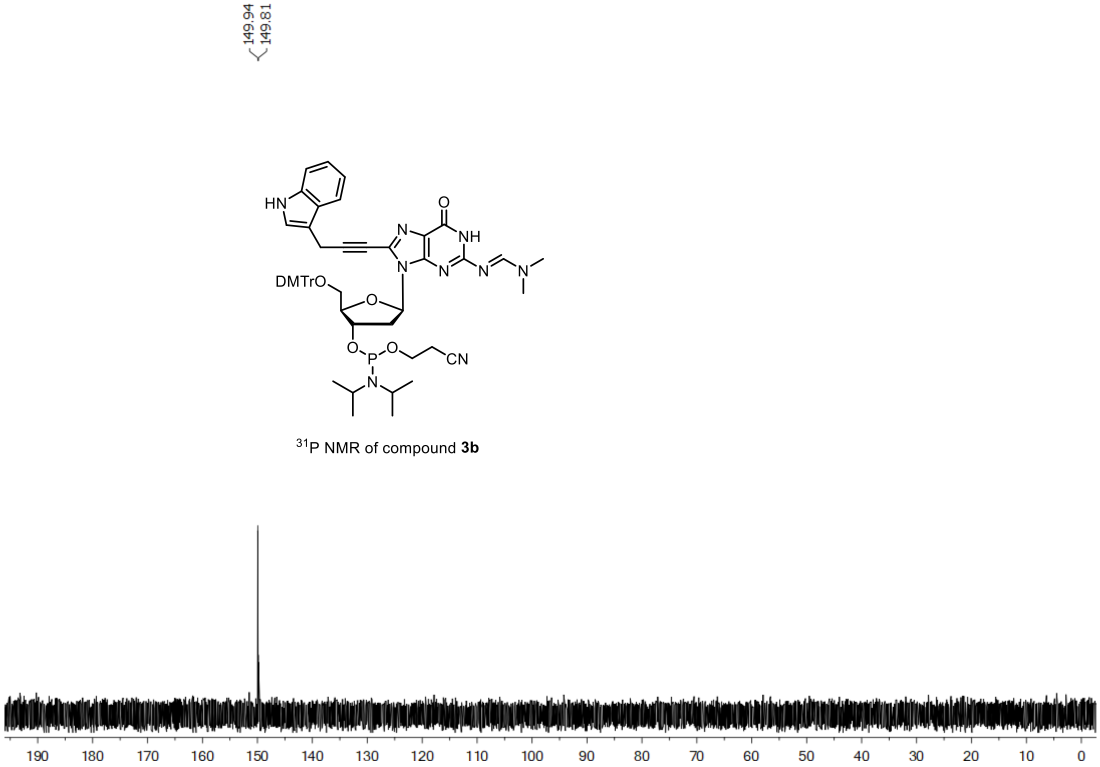

Figure S73 ^{31}P NMR spectrum of compound **3b** (CD_3CN)

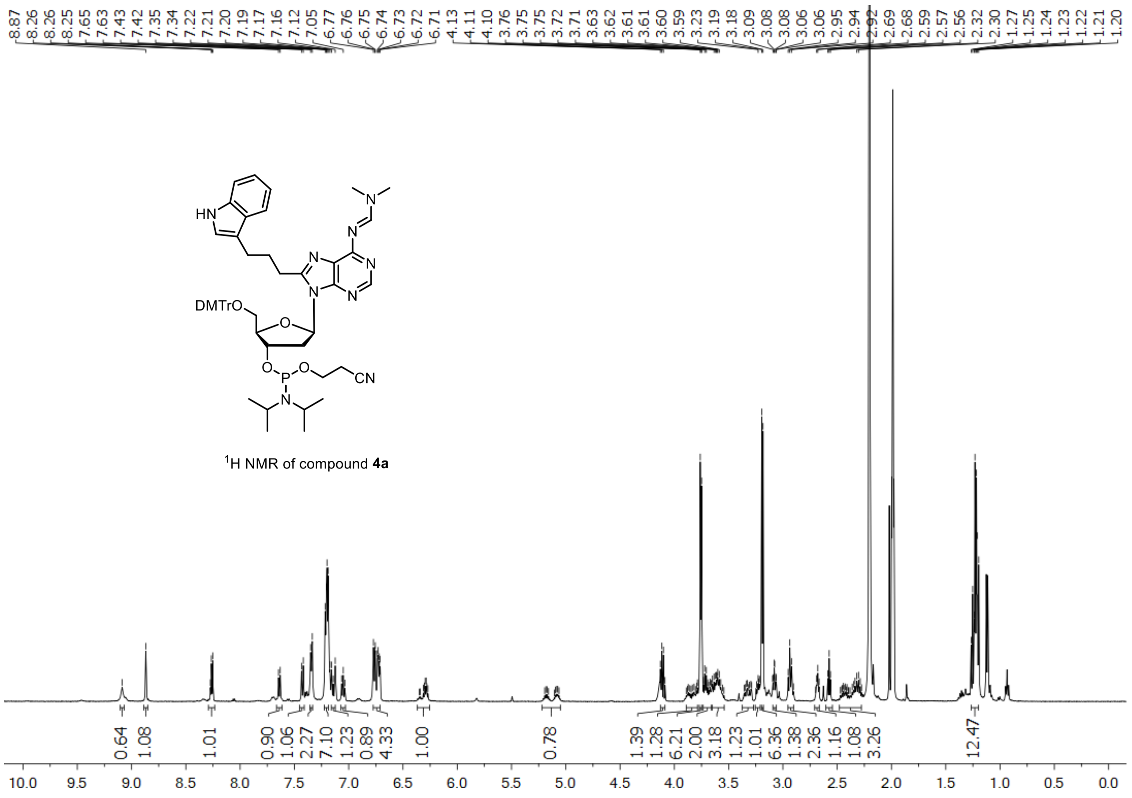

Figure S74 ^{1}H NMR spectrum of compound **4a** (CD_3CN)

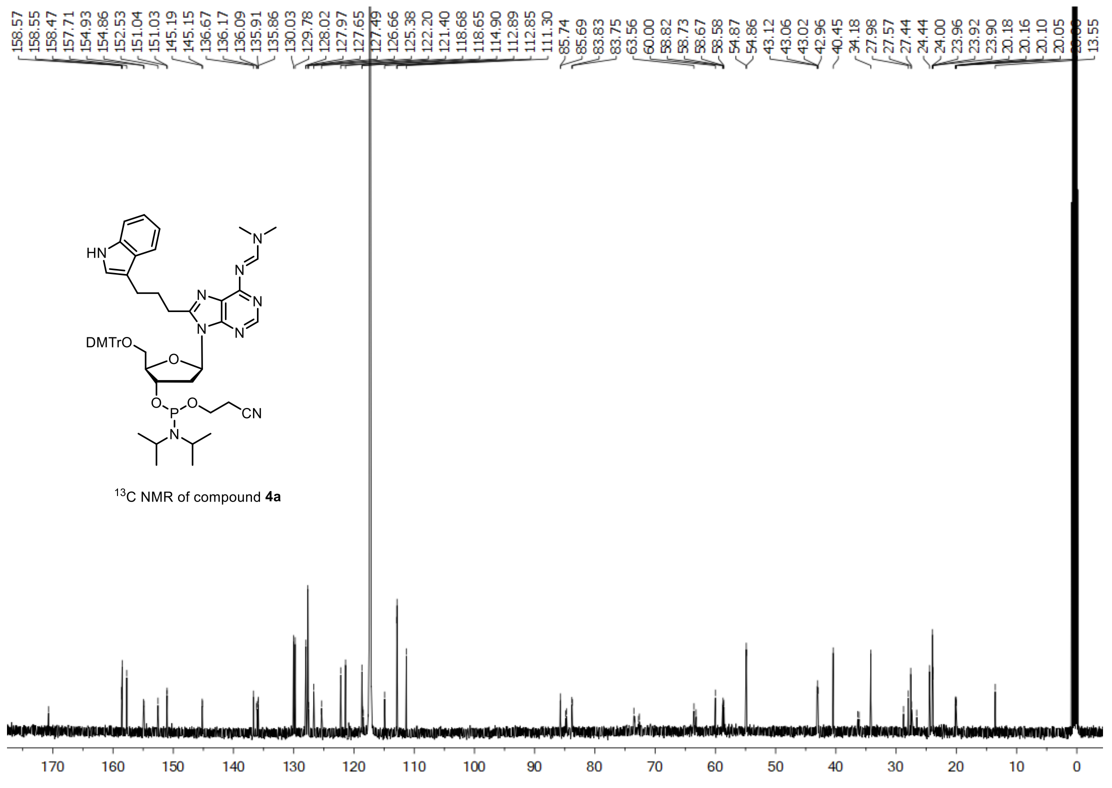

Figure S75 ^{13}C NMR spectrum of compound **4a** (CD_3CN)

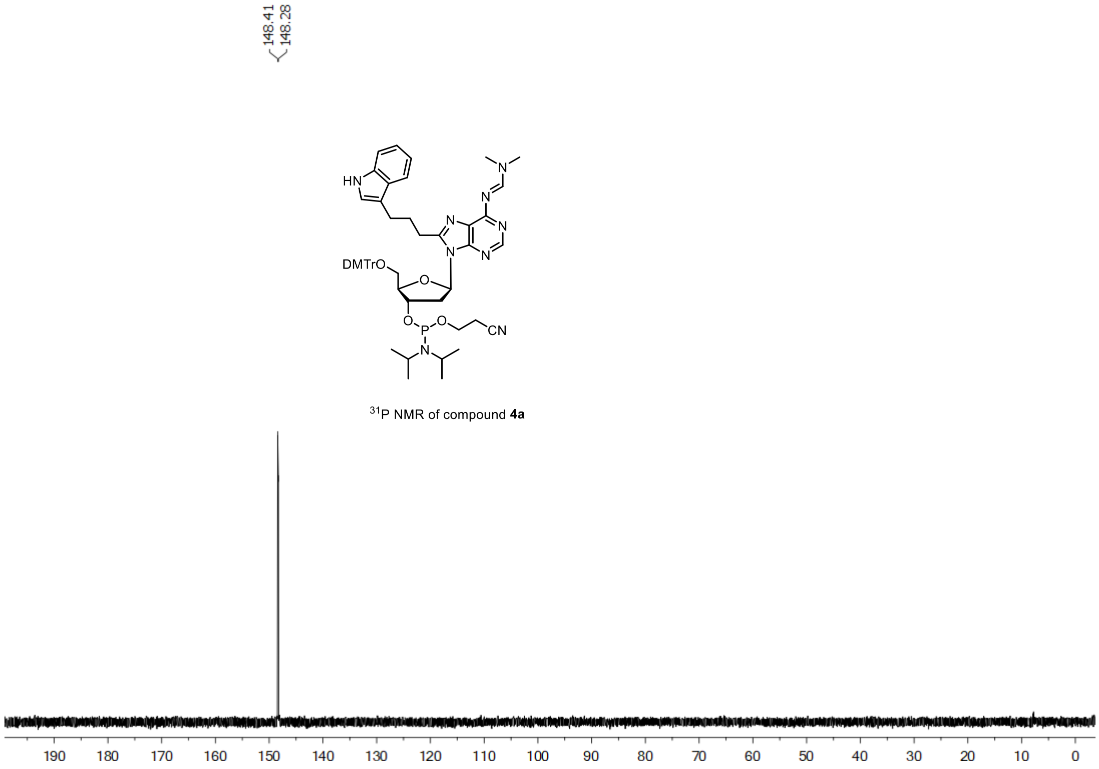

Figure S76 ^{31}P NMR spectrum of **4a** (CD_3CN)

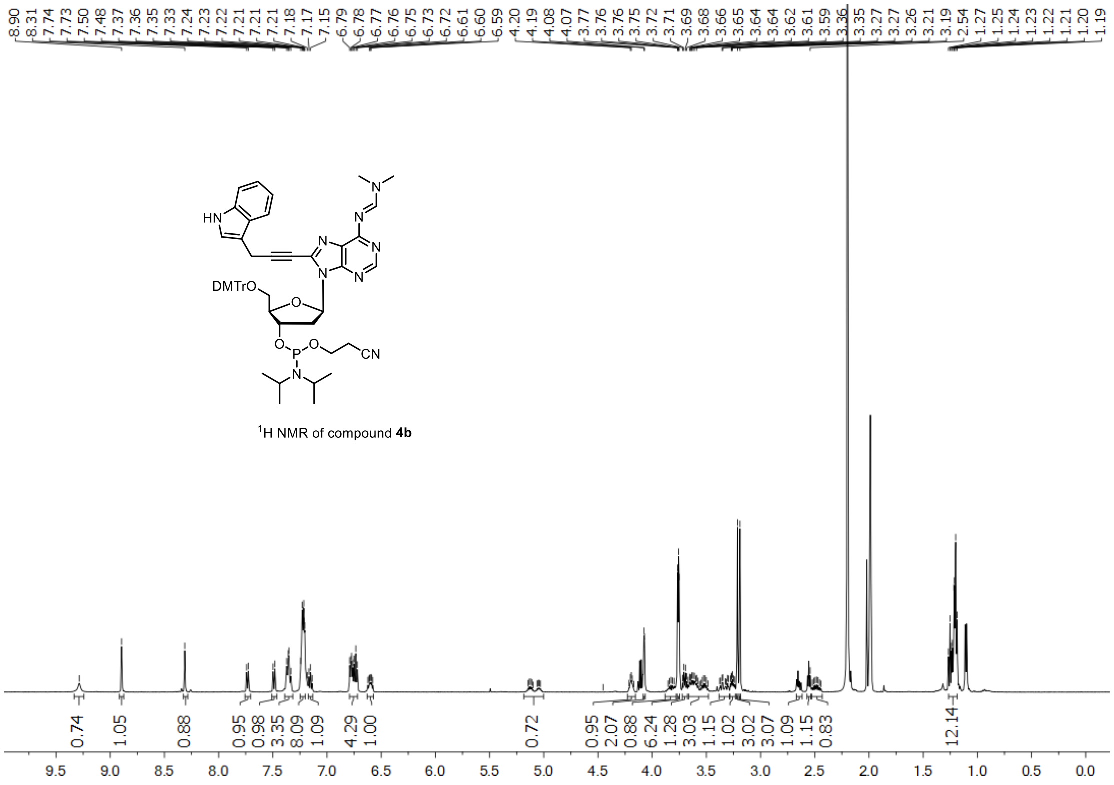

Figure S77 ^{1}H NMR spectrum of compound **4b** (CD_3CN)

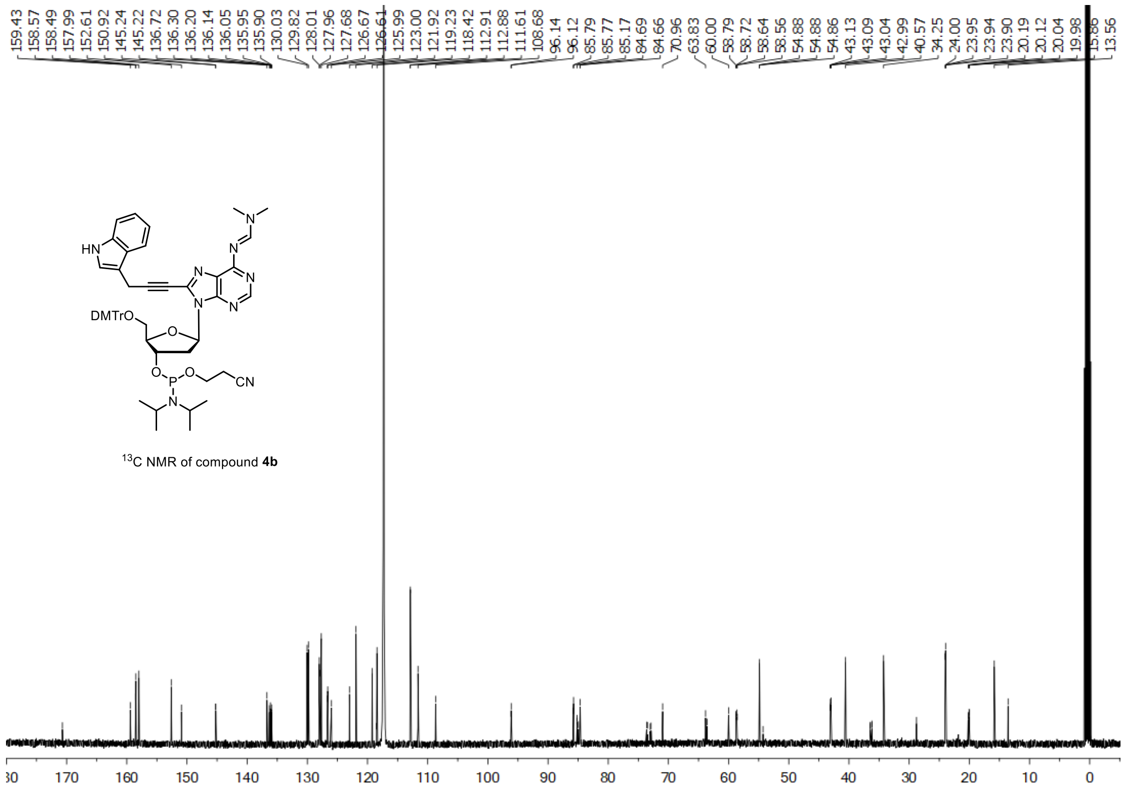

Figure S78 ^{13}C NMR spectrum of compound **4b** (CD_3CN)

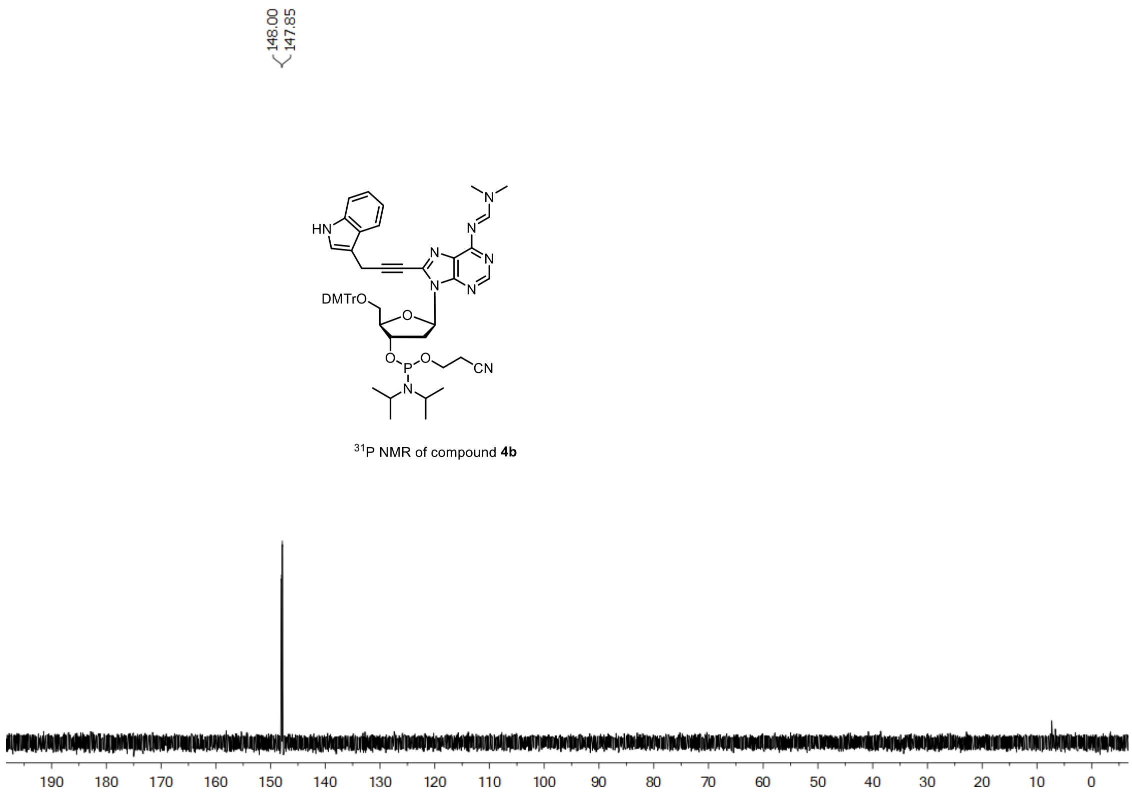

Figure S79 ^{31}P NMR spectrum of compound **4b** (CD_3CN)

6. Mass Spectra of DNA sequences

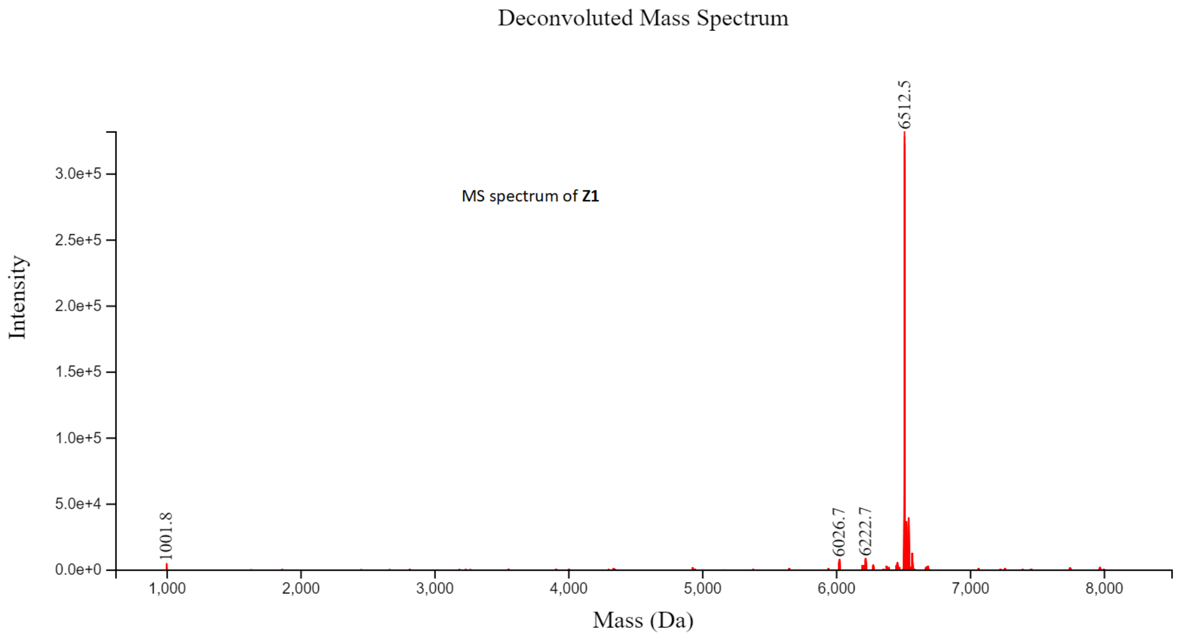

Figure S80 Deconvoluted MS spectrum of **Z1**

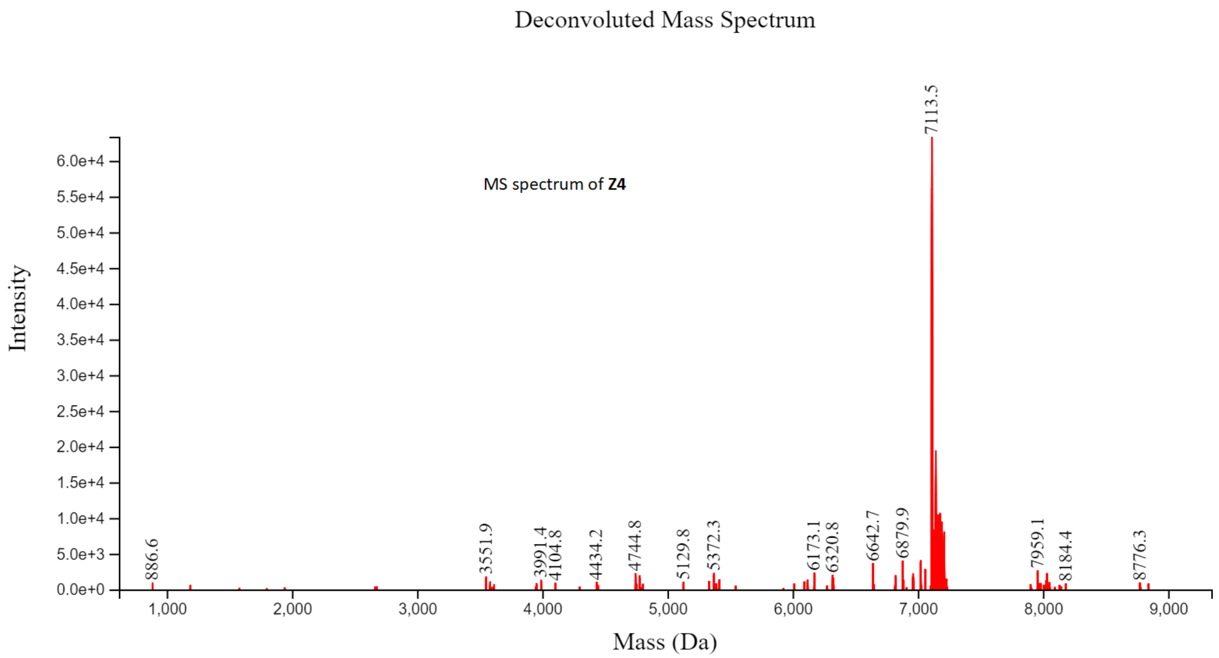

Figure S81 Deconvoluted MS spectrum of **Z4**

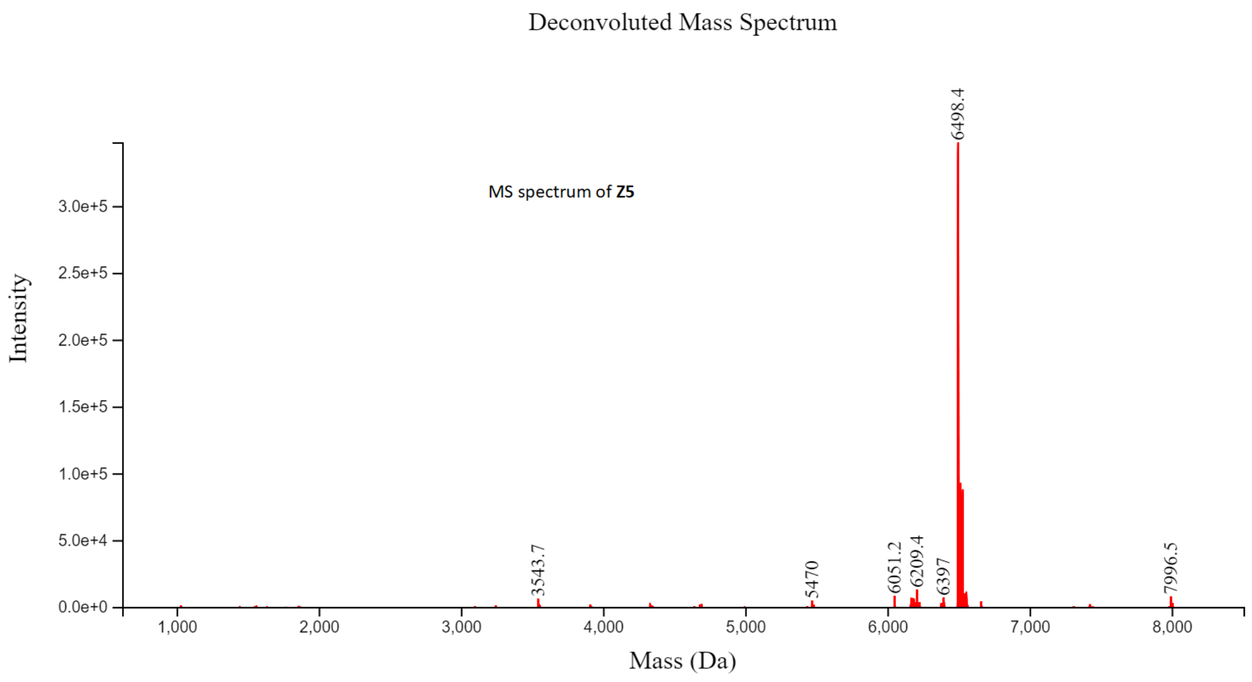

Figure S82 Deconvoluted MS spectrum of **Z5**

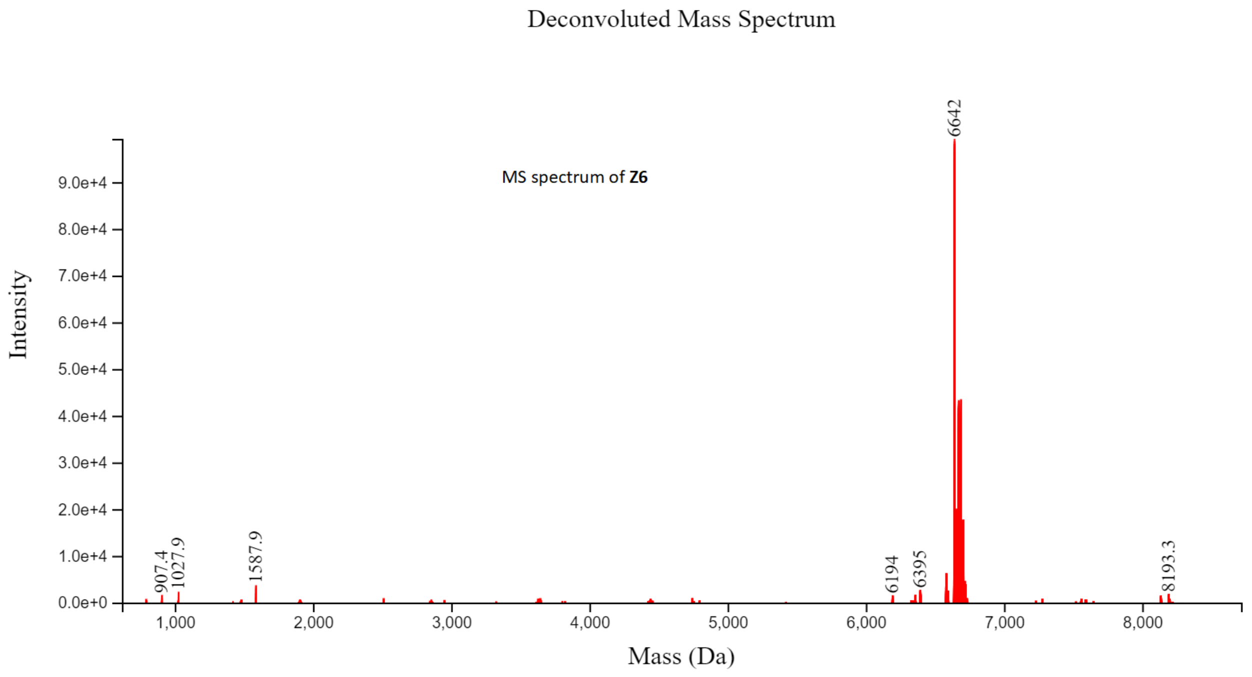

Figure S83 Deconvoluted MS spectrum of **Z6**

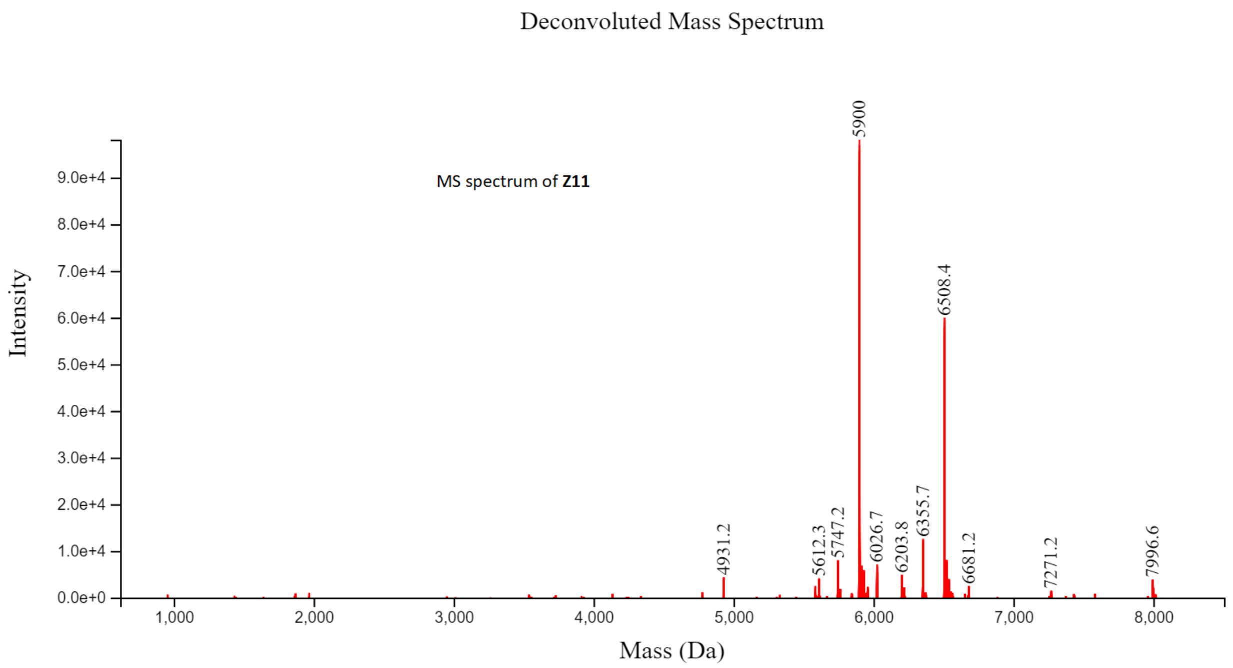

Figure S84 Deconvoluted MS spectrum of **Z11**

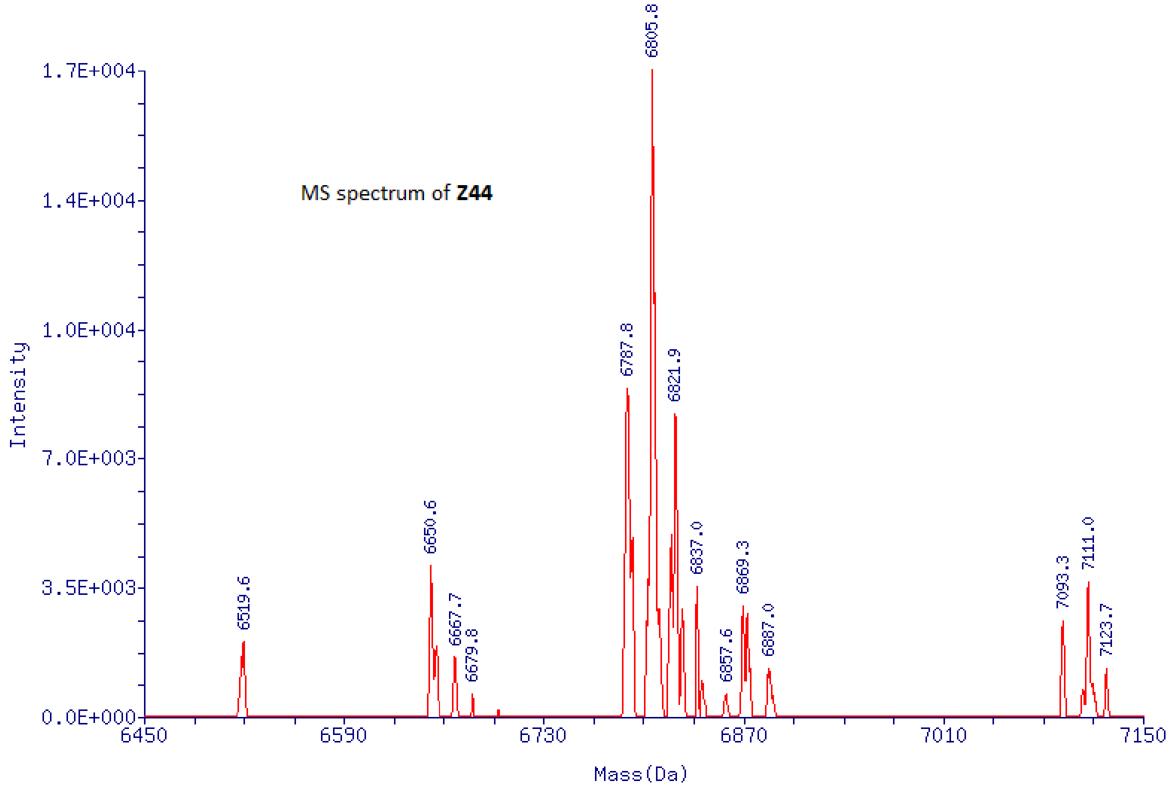

Figure S85 Deconvoluted MS spectrum of **Z44**

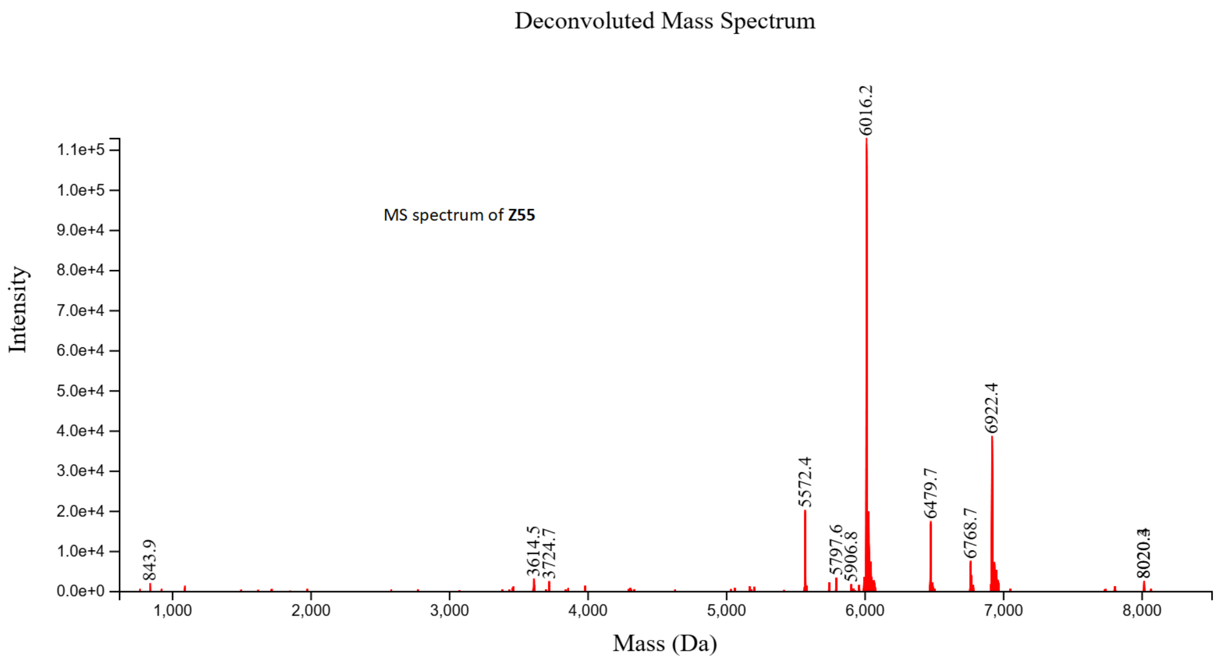

Figure S86 Deconvoluted MS spectrum of **Z55**

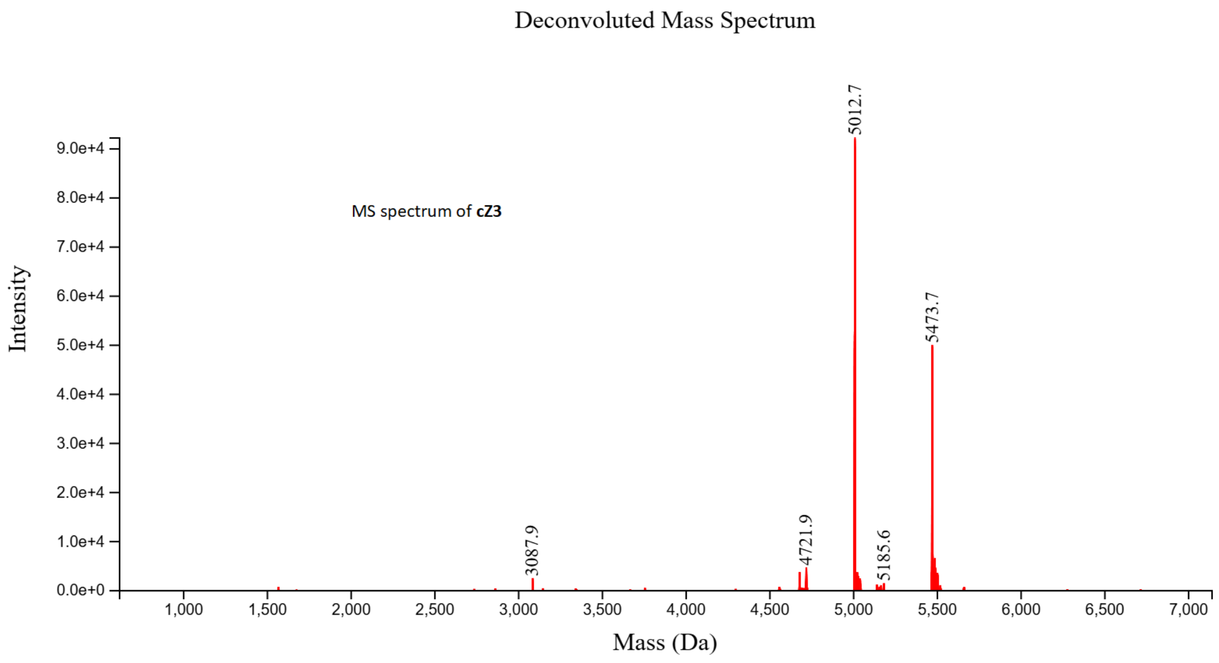

Figure S87 Deconvoluted MS spectrum of **cZ3**

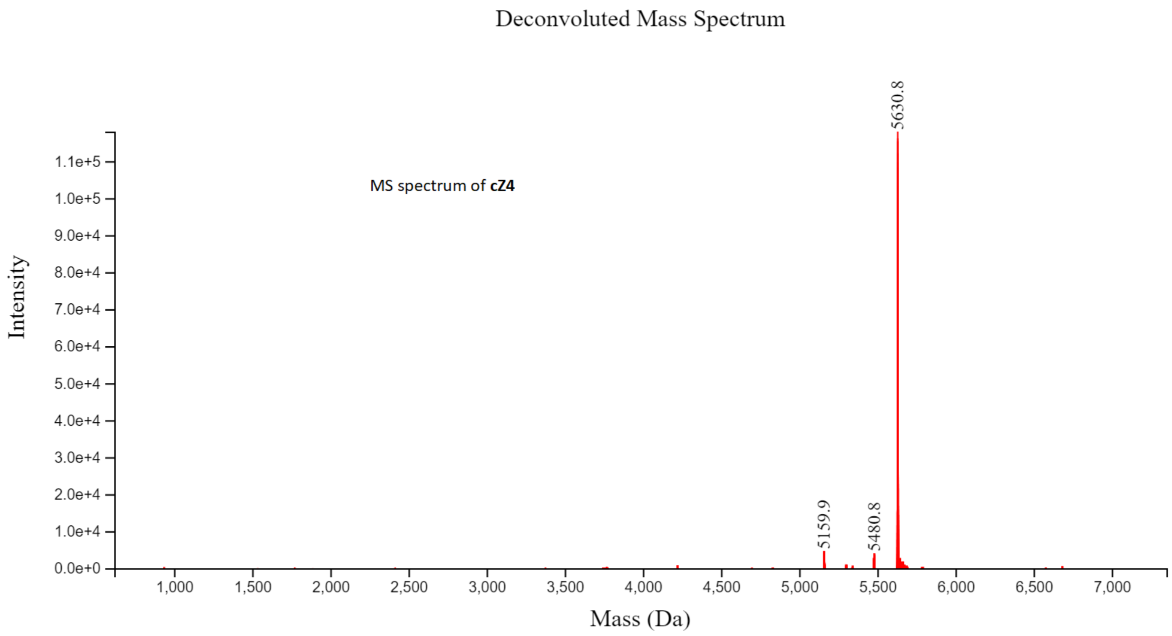

Figure S88 Deconvoluted MS spectrum of **cZ4**

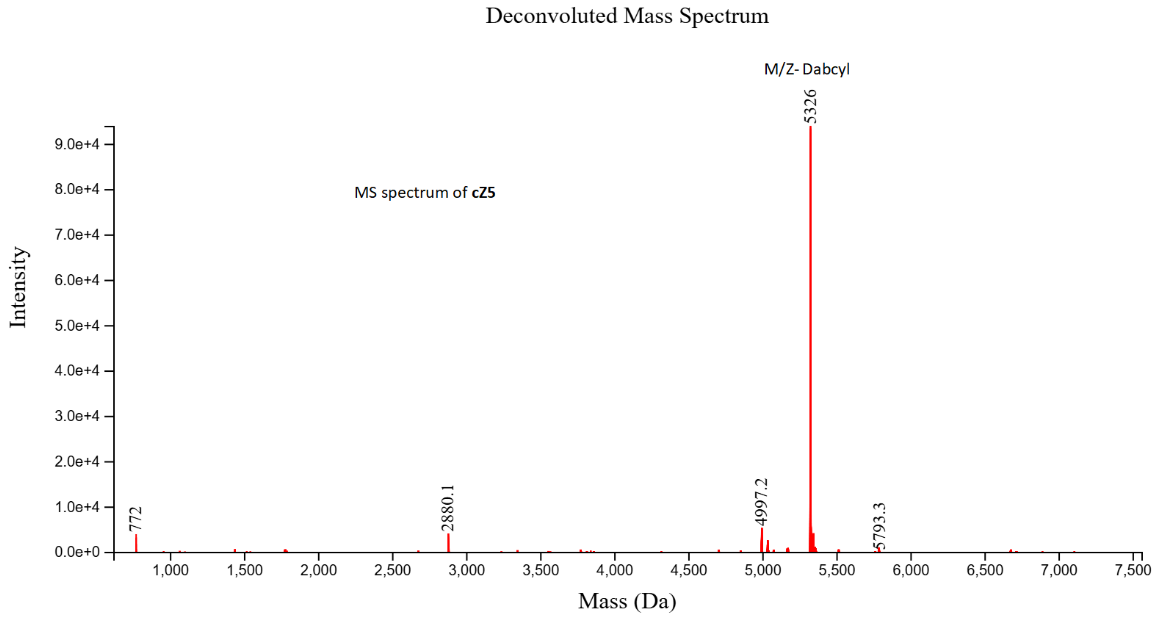

Figure S89 Deconvoluted MS spectrum of **cZ5**

6. Reference

1. Yu, R. T.; Friedman, R. K.; Rovis, T. Enantioselective Rhodium-Catalyzed [4+2+2] Cycloaddition of Dienyl Isocyanates for the Synthesis of Bicyclic Azocine Rings. *J. Am. Chem. Soc.* **2009**, *131* (37), 13250–13251. DOI: 10.1021/ja906641d.
